# A standardised framework to identify optimal animal models for efficacy assessment in drug development

**DOI:** 10.1371/journal.pone.0218014

**Published:** 2019-06-13

**Authors:** Guilherme S. Ferreira, Désirée H. Veening-Griffioen, Wouter P. C. Boon, Ellen H. M. Moors, Christine C. Gispen-de Wied, Huub Schellekens, Peter J. K. van Meer

**Affiliations:** 1 Department of Pharmaceutics, Utrecht Institute for Pharmaceutical Sciences, Utrecht University, Utrecht, The Netherlands; 2 Copernicus Institute of Sustainable Development, Innovation Studies, Utrecht University, Utrecht, The Netherlands; 3 Medicines Evaluation Board, Utrecht, The Netherlands; University of Edinburgh, UNITED KINGDOM

## Abstract

**Introduction:**

Poor translation of efficacy data derived from animal models can lead to clinical trials unlikely to benefit patients–or even put them at risk–and is a potential contributor to costly and unnecessary attrition in drug development.

**Objectives:**

To develop a tool to assess, validate and compare the clinical translatability of animal models used for the preliminary assessment of efficacy.

**Design and results:**

We performed a scoping review to identify the key aspects used to validate animal models. Eight domains (Epidemiology, Symptomatology and Natural History–SNH, Genetic, Biochemistry, Aetiology, Histology, Pharmacology and Endpoints) were identified. We drafted questions to evaluate the different facets of human disease simulation. We designed the Framework to Identify Models of Disease (FIMD) to include standardised instructions, a weighting and scoring system to compare models as well as factors to help interpret model similarity and evidence uncertainty. We also added a reporting quality and risk of bias assessment of drug intervention studies in the Pharmacological Validation domain. A web-based survey was conducted with experts from different stakeholders to gather input on the framework. We conducted a pilot study of the validation in two models for Type 2 Diabetes (T2D)–the ZDF rat and db/db mouse. Finally, we present a full validation and comparison of two animal models for Duchenne Muscular Dystrophy (DMD): the mdx mouse and GRMD dog. We show that there are significant differences between the mdx mouse and the GRMD dog, the latter mimicking the human epidemiological, SNH, and histological aspects to a greater extent than the mouse despite the overall lack of published data.

**Conclusions:**

FIMD facilitates drug development by serving as the basis to select the most relevant model that can provide meaningful data and is more likely to generate translatable results to progress drug candidates to the clinic.

## Introduction

The use of non-human animals to evaluate the safety of new drugs is an integral part of the regulatory research and development process [1,2]. Established at a time when laboratory animals were one of the most complex systems available, they are still considered as the gold standard. However, despite their apparent value as a drug testing system to predict safety and efficacy in humans, scientists are increasingly aware of their considerable drawbacks and limited predictivity [3–6].

While no apparent toxicity in poorly predictive animal models can lead to possible harm to patients, false toxic signals might prevent potentially safe drugs from reaching the market. At the same time, models which overestimate the efficacy of new drugs lead to clinical trials with drugs that have a modest effect at best or are entirely ineffective at worst–meaning we could be effectively putting patients at risk for no possible benefit [5,7].

We previously assessed the value of regulatory safety studies in a public-private research consortium which consisted of pharmaceutical company stakeholders, the Dutch regulatory agency and academia [8]. This partnership was unique in that it allowed proprietary data to be used for our primary analyses, which could then be presented in an aggregated, anonymised fashion to propose policy changes [9–13]. A key finding of these studies was that regardless of non-human primate (NHP) models having the closest biological resemblance to humans, their indiscriminate use in the safety testing of new biotechnology products (e.g. mAbs) as well as to demonstrate the similarity of biosimilar to reference products often adds limited value to the preclinical package. When taken all together, these results suggest the mandatory use of non-human animal safety testing according to current guidelines should be reconsidered.

Contrary to safety assessment, the evaluation of efficacy is not subject to formalised guidance or regulations since each new drug warrants a tailor-made approach based on its mechanism of action and indication [14,15]. Consequently, predefining which assays or models to be used to test new drugs’ efficacy, as done for safety, could jeopardise innovative companies’ ability to develop such drug-specific strategies.

Nevertheless, most late-stage clinical trials, which are frequently based on efficacy data from non-human animal studies fail due to the lack of efficacy [16–20]. The low internal validity (i.e. the methodological qualities of an experiment, such as randomisation and blinding) of non-human animal research has been frequently suggested as a likely cause for such poor translation to the clinic [21–25]. For example, non-randomised and non-blinded studies are 3.4 and 3.2 times more likely to report positive results, respectively [21]. Other factors, like housing and husbandry, can also significantly affect animal models’ behaviour and other phenotype parameters, such as neurotransmitter levels [26].

Several projects aimed at improving design and reporting standards of preclinical studies have been initiated in the past decade. The ARRIVE guidelines were the first harmonised guidelines to establish reporting standards in animal research [27]. In the same vein, the PREPARE guidelines provide extensive guidance on the key parameters necessary to design and conduct higher quality animal experiments [28]. With the growing popularity of systematic reviews and meta-analysis of non-human animal studies, the use of the GRADE approach to grade the quality of evidence and strength of the recommendations given has also been suggested [29]. The European Quality In Preclinical Research (EQIPD), funded by the Innovative Medicines Initiative (IMI), was introduced in 2017 aiming at improving the robustness of preclinical data, focusing mostly on neuroscience research [30]. All these initiatives now allow researchers to address these issues effectively.

The inadequate assessment of the external validity of efficacy models (i.e. how well non-human animal results are generalisable to the human situation) is also an important factor for poor translation [4]. Currently, drug developers frequently rely on the well-established criteria of face, construct and predictive validity to assess the external validity of animal models [31,32]. Face validity refers to how well an animal model reproduces the clinical symptoms and manifestations of a disease; construct validity refers to how close the underlying biology thought to be causing these symptoms in the human condition is replicated in the animal model; and predictive validity refers to how similar to a human an animal model responds to clinically effective drugs [32,33]. Because none of these criteria is integrated nor present a systematic way to assess the ability of an animal model to predict drug efficacy in humans, they are highly susceptible to user interpretation. The absence of standardisation leads to animal models being assessed by different disease parameters, which further complicates a scientifically relevant comparison between them.

A tool applicable to *in vitro* and *in vivo* settings was suggested by Sams-Dodd and further refined by Denayer et al. [34,35]. It scores five categories (species, disease simulation, face validity, complexity and predictivity) from 1 to 4, a score of 4 being the closest to the patient. Nonetheless, this tool fails to capture the nuances of most characteristics potentially relevant for the demonstration of efficacy (e.g. genes, biomarkers, histology) to make it informative and usable to evaluate the external validity of animal models.

## The Framework to Identify Models of Disease (FIMD)

The existing approaches for assessing external validity cannot be used by researchers to find what is the most relevant model to demonstrate the efficacy of a drug based on its mechanism of action and indication. Here, we present a method to assess the various aspects of the external validity of efficacy models in an integrated manner–the Framework to Identify Models of Disease (FIMD). A ‘model of disease’ is here used for any animal model that simulates a human condition for which a drug can be developed.

### Questionnaire draft

To answer our research question “what are the main parameters used to validate animal models of disease?”, we performed a scoping review using the search strategy "animal model"[tiab] AND validation[tiab] based on Medical Subjects Heading (MeSH) terms in PubMed on 03.06.2016. The search was not designed to be an exhaustive account of the available literature, but rather as a snapshot of the core aspects used by researchers to validate disease models. Articles were included if they were published in English and if they contained details about the validation of a new animal model or optimisation of an existing one aiming at drug screening. Articles were excluded if the aim of validation was behavioural tests or biomarkers; if the model aimed at evaluating a surgical technique; and/or if they were not available via the university’s online library. Titles and abstracts were screened, and publications which met the inclusion criteria were then read in full-text.

**Fig 1:**
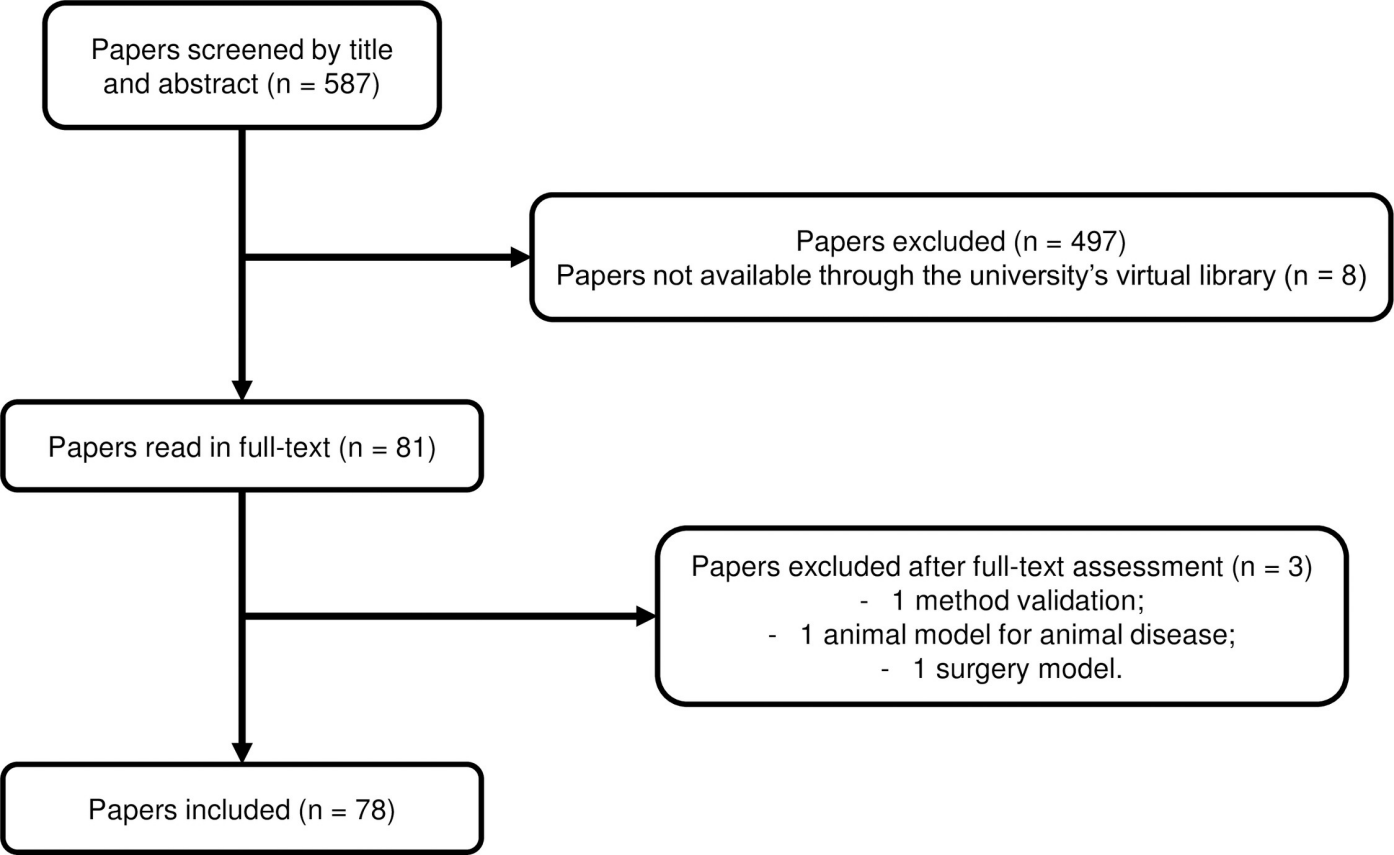
Literature search process. Identification, screening and inclusion of articles for the identification of validation parameters in the literature.

A total of 587 records were screened by title and abstract, of which eight were not available through the university’s virtual library. Eighty-one (81) articles were selected for full-text assessment. Of these, 78 records (59 of development or optimisation of animal models and 19 reviews) met the inclusion criteria. The literature search process is shown in Fig 1.

The thematic content analysis (TCA) method was used to identify the main validation parameters present in the literature [36]. Briefly, this method consists of collecting information on a field in a structured way, followed by extracting the data from the identified sources and, finally, grouping these parameters into more general, broader categories. In our study, we determined the parameters defined by the included articles’ authors as determinative to ‘validate’ the animal model. Identified parameters were then grouped into themes–based on the concepts of face, construct and predictive validity–and were given a generic, broader name. Eight domains were identified based on the validation parameters reported in the literature and are presented in Table 1.

**Table 1:** The eight domains identified and their frequency in the included articles.

Domain	Description	Frequency (n = 79)
Epidemiology	related to age and sex	12
Symptomatology and Natural History (SNH)	related to symptoms and natural history of disease	62
Genetics	related to genes, genetic alterations and expression	19
Biochemistry	related to biomarkers	43
Aetiology	related to the aetiology	26
Histology	related to the histopathological features	37
Pharmacological	related to the effect of effective and ineffective drugs	39
Endpoints	related to the endpoints and the methods to assess thereof chosen for the pharmacological studies	9

The eight identified domains were used to draft questions related to the different facets of disease simulation (Table 2).

**Table 2:** The Framework to Identify Models of Disease (FIMD). Questions per validation parameter, and weight of each question and domain according to the same weighting system, in which the total of 100 points is divided equally by the eight domains, and then equally by each question within a domain.

	Weight
1. EPIDEMIOLOGICAL VALIDATION	12,5
1.1 Is the model able to simulate the disease in the relevant sexes?	6,25
1.2 Is the model able to simulate the disease in the relevant age groups (e.g. juvenile, adult or ageing)?	6,25
2. SYMPTOMATOLOGY AND NATURAL HISTORY VALIDATION	12,5
2.1 Is the model able to replicate the symptoms and co-morbidities commonly present in this disease? If so, which ones?	2,5
2.2 Is the natural history of the disease similar to human’s regarding: 2.2.1 Time to onset	2,5
2.2.2 Disease progression	2,5
2.2.3 Duration of symptoms	2,5
2.2.4 Severity	2,5
3. GENETIC VALIDATION	12,5
3.1 Does this species also have orthologous genes and/or proteins involved in the human disease?	4,17
3.2 If so, are the relevant genetic mutations or alterations also present in the orthologous genes/proteins?	4,17
3.3 If so, is the expression of such orthologous genes and/or proteins similar to the human condition?	4,16
4. BIOCHEMICAL VALIDATION	12,5
4.1 If there are known pharmacodynamic (PD) biomarkers related to the pathophysiology of the disease, are they also present in the model?	3,125
4.2 Do these PD biomarkers behave similarly to humans’?	3,125
4.3 If there are known prognostic biomarkers related to the pathophysiology of the disease, are they also present in the model?	3,125
4.4 Do these prognostic biomarkers behave similarly to humans’?	3,125
5. AETIOLOGICAL VALIDATION	12,5
5.1 Is the aetiology of the disease similar to humans’?	12,5
6. HISTOLOGICAL VALIDATION	12,5
6.1 Do the histopathological structures in relevant tissues resemble the ones found in humans?	12,5
7. PHARMACOLOGICAL VALIDATION	12,5
7.1 Are effective drugs in humans also effective in this model?	4,17
7.2 Are ineffective drugs in humans also ineffective in this model?	4,17
7.3 Have drugs with different mechanisms of action and acting on different pathways been tested in this model? If so, which?	4,16
8. ENDPOINT VALIDATION	12,5
8.1 Are the endpoints used in preclinical studies the same or translatable to the clinical endpoints?	6,25
8.2 Are the methods used to assess preclinical endpoints comparable to the ones used to assess related clinical endpoints?	6,25

We designed FIMD to circumvent some significant limitations present in the current approaches, such as the absence of standardisation, integration and facility of model comparison. We also conducted a web-based survey with experts from academia, industry and regulatory agencies to validate our findings and identify opportunities for improvement (see S1 Supporting Information). To make the framework systematic, transparent and to minimise parameter discordance, instructions on how to complete the validation sheets as well as a template are provided (see S2 and S3 Supporting Information, respectively). Non-human animal studies of both effective and ineffective drugs are included in the Pharmacological Validation. This inclusion results in a better understanding of the pathways that are involved in the disease pathophysiology of a model when compared to humans. With this information, companies with extensive non-human animal data from failed projects can easily perform a read-across of their models to inform the choice of future programmes. Also, all interventional drug studies in the Pharmacological Validation have a reporting quality and risk of bias assessment adapted from the ARRIVE guidelines and the SYRCLE’s Risk of Bias Tool (see S2 Supporting Information) [27,37].

### Weighting and scoring system

To facilitate the comparison between animal models in the same indication and ultimately, the choice of the best fit for investigating a drug’s efficacy, we developed a weighting and scoring system. This system was developed based on a maximum score of 100 points. The 100 points were equally distributed across the eight domains (12.5 points per domain) and in each domain, evenly distributed per question–the same weight (SW) system. Table 2 shows the weighting per domain and question according to the same weighting system. Although an indication-specific weighting system could potentially improve the sensitivity of the score, there is not enough data to accurately determine weightings for each question based on disease characteristics. At this stage, the presence of such a feature is hardly justifiable given the complexity it would add to the overall framework. Thus, we opted not to add indication-specific weighting systems in this version. Examples of how to score the questions are provided in S2 Supporting Information.

Calculating the score from each domain allows easy visualisation in a radar plot, which is a key product of FIMD (Box 1). The final score is an indication of the degree to which a model simulates the human condition. While it is not validated at this point, it allows researchers to identify the strengths and weaknesses of an animal model at a glance. The underlying (qualitative) data will further determine which model is the most relevant to test a given drug. Hence, the best fit will not necessarily be the model with the highest score but rather the model that more closely mimics the pathways involved in the mechanism of action of a drug.

**Box 1. Validation domains overview with a radar plot**

**Fig 2:**
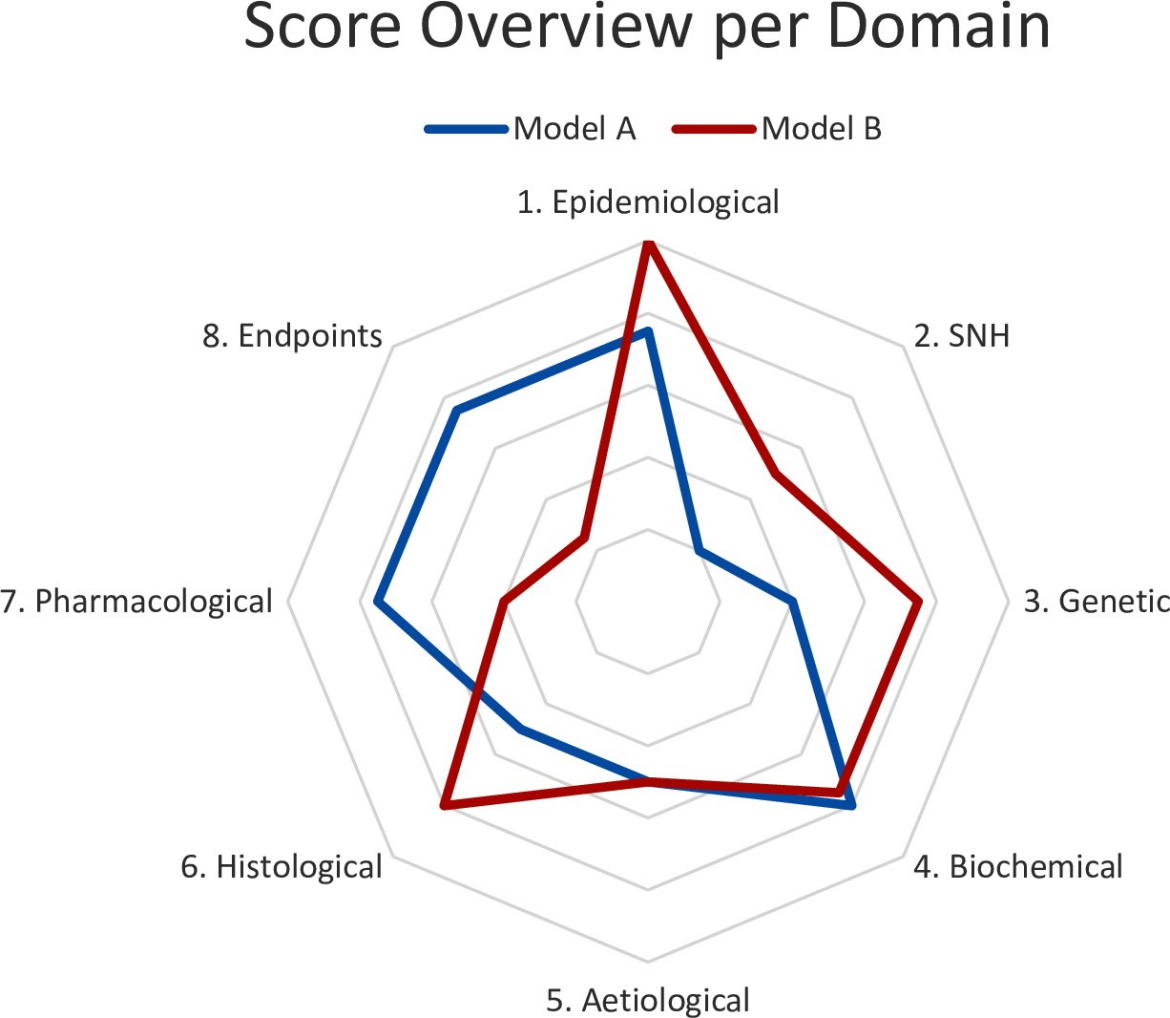
Hypothetical radar plot. Radar plot with hypothetical scores per parameter per model. The closer a parameter is to the edge, the better the model simulates that aspect of the human disease. SNH–Symptomatology and Natural History.

The radar plot is generated by using the ratio of each domain’s score (sum of each question within a domain) to the maximum possible score. For instance, if a model scores 6.25 points in the first domain, Epidemiological Validation (3.125 per question), of a total of 12.5 points, the ratio is 0.5 (6.25/12.5). These ratios are calculated for each domain and then aggregated in the radar plot, which is a relative demonstration of how close an animal model can simulate the human condition. The closer a domain is to the edge, the closer the model mimics that specific aspect of the human disease (ratio of 1). Radar plots can include one or more models. An example is given in Fig 2.

Next to the weighting and scoring system, two factors were created to contextualise the final score: the uncertainty and the similarity factors. Although these factors are not used in the score calculation, they help to explain the differences or absence thereof in the score between models. The uncertainty factor is used to inform how much information is missing in the validation of a model–how many questions were answered with ‘unclear’. ‘Unclear’ is used to answer questions for which no data is available or conflicting evidence is found. The similarity score is used when comparing two models, informing about how often questions received the same answer in both models. It indicates how comparable two models are. Further details on how to calculate and interpret the uncertainty and similarity factor are available in S2 Supporting Information, sections D and E.

### Validating efficacy models in FIMD

We addressed the lack of a clear definition of what the minimum requirements to validate an animal model are from previous approaches. There are several interpretations of what the validation of an assay or model should entail and how to assure its reproducibility. For the OECD and EURL-ECVAM, validation means establishing a statistically rigid range for several criteria to describe a test’s ability to be reproduced reliably [38]. This validation is frequently an expensive and lengthy process, mostly applicable to *in vitro* tests, which would not be practical or add much value to efficacy assessment in animal models [14]. A more applied approach is presented by ICHS5(R3), in which the definition of the context of use (i.e. the conditions in which the assay results can be relied upon) is the guiding factor for the validation of a test [39].

Our validation definition aims to provide the evidence for an efficacy model’s context of use (i.e. the intended indication for which drugs are being developed). Although animal models are not expected to mimic the human condition completely, identifying which aspects they can reproduce and to which extent is crucial to determining whether they are the most suitable choice. Therefore, we established four levels of confidence in the validation of animal models in their context of use based on the percentage of definite answers to the eight validation domains (see Table 3). A ‘definite answer’ is defined as any answer except for ‘unclear’.

**Table 3:** Different levels of validation according to the percentage of definite answers to the questionnaire.

(%) Definite Answers	Validation Level
0–40	Insufficiently validated
41–60	Slightly validated
61–80	Moderately validated
81–100	Highly validated

The product of FIMD is a validation sheet of an animal model for an indication, which provides the necessary information for its assessment as a potential model to demonstrate a drug’s efficacy. We have conducted a pilot study and a full validation in one indication each to test the main features of FIMD: Type 2 Diabetes (T2D) and Duchenne Muscular Dystrophy (DMD), respectively (see S4 and S5 Supporting Informations). We provide a stepwise approach to the use of FIMD in Box 2 with DMD as an example. The summarised results of the full validation are presented in Box 3. The results from the reporting quality and risk of bias assessments are provided in Box 4. The results per individual study are shown in S6 Supporting Information (PV Secondary Assessments–T2D) and S7 Supporting Information (PV Secondary Assessments–DMD). A score calculator is available in S8 Supporting Information.

**Box 2. A Stepwise guide to FIMD: The example of DMD**

We chose DMD because it is caused by mutations in a single gene (known aetiology) and of the limited availability of effective therapies [40]. After an initial literature search to identify reviews models of DMD using the string ‘(“animal model” OR “animal models”) AND (“Duchenne Muscular Dystrophy” OR DMD) AND review’, we selected the mdx mouse and the Golden Retriever Muscular Dystrophy (GRMD) dog. The mdx mouse was chosen because it is the most commonly used model and the GRMD dog for better replicating the symptoms and histological features of DMD [41,42]. Below we present a stepwise approach to complete FIMD, including examples from the validation of the DMD models.

1. **Use MeSH and Emtree to identify all terminology related to the model and indication of interest, including plurals**;
  In PubMed, there are no MeSH terms for either the mdx mouse nor the GRMD dog, so we opted for the use of ‘mdx’ and ‘GRMD’ as general, broad terms of the subsequent searches. In Embase, we used the terms from the Emtree (see S5 Supporting Information, questions 7.1 and 7.2).
2. **Organise an expert group to define the disease parameters. If that is not possible, search for reviews on the human disease and animal models for that condition. Whenever necessary, justify the choice of disease parameters**;
  We could not set up an expert group for the definitions of the disease parameters. We started by using the terms identified in the previous item to search for general reviews on the models. We found most of the effective and ineffective drugs in these reviews combined with searches in ClinitralTrials.gov and the free version of AdisInsight. Using reviews’ reference lists and forward citations, we identified most papers used in the validation. Since we could not find any prognostic biomarkers for DMD, we decided to exclude questions 4.3 and 4.4 (both related to prognostic biomarkers), adding a justification and the references on which we based our decision.
3. **Answer the questions in the validation sheet template (S3 Supporting Information) based on the information found during the search for papers. If, in the end, some questions are still unanswered, use specific search strings for these questions**;
  Often papers will have data that can be used to answer more than one question. Just by reading reviews, their references and using forward citation on Google Scholar, we could answer all questions from FIMD. We did not have to use any specific search strings.
4. **For the Pharmacological Validation, assess each paper based on the instructions for questions 7.1, 7.2; and S2 Supporting Information, sections F1. Reporting Quality and F2. Risk of Bias. Fill this information in a separate spreadsheet file (such as S7 Supporting Information)**;
  We assessed 32 articles for the Pharmacological Validation of the mdx mouse and 3 for the GRMD dog. A report per paper, as well as aggregated data, is provided in S7 Supporting Information. A summary of the reporting quality and risk of bias assessment was provided for each drug (see S5 Supporting Information).
5. **Fill in the Score Calculator (S8 Supporting Information) to get the total score and the radar plot**;
  All the answers were filled in the score calculator. We had to adjust the calculation of the final score to account for the exclusion of questions 4.3 and 4.4, resulting in a total max score of 93.75. We calculated the equivalent score to 100 ($\frac{score\times 100}{93.75}$), which for the mdx mouse was 67.78 and for GRMD dog was 65.41. The radar plot is automatically generated once all fields are filled out.
6. **Calculate the Uncertainty Factor according to section D**;
  More details on how to calculate the uncertainty factor are provided in S2 Supporting Information, section D The mdx mouse had an uncertainty factor of 6.1% while the GRMD dog had 18.2%. In both models, all ‘unclear’ answers were given to sections in the Pharmacological Validation, especially for the GRMD dog, which had only three studies with one of the five drugs identified.
7. **If another model has been validated for the same indication, calculate the Similarity Factor according to section E**;
  More details on how to calculate the similarity factor are provided in S2 Supporting Information, section E. The similarity factor was 48.5%, meaning that less than half of the questions were answered in the same way, which indicates there are considerable differences between the mdx mouse and the GRMD dog.
8. **Finalising the validation sheet**;
  Once the final score, the uncertainty and similarity factors are calculated, fill in this information in the first page of the validation sheet. Include the validation date (latest date of publication of the validation references). Determine the validation level as defined in Table 3. Describe the historical background of the model as described in S2 Supporting Information. Fill each question’s score.

**Box 3. Using FIMD to assess and compare efficacy models**

**Fig 3:**
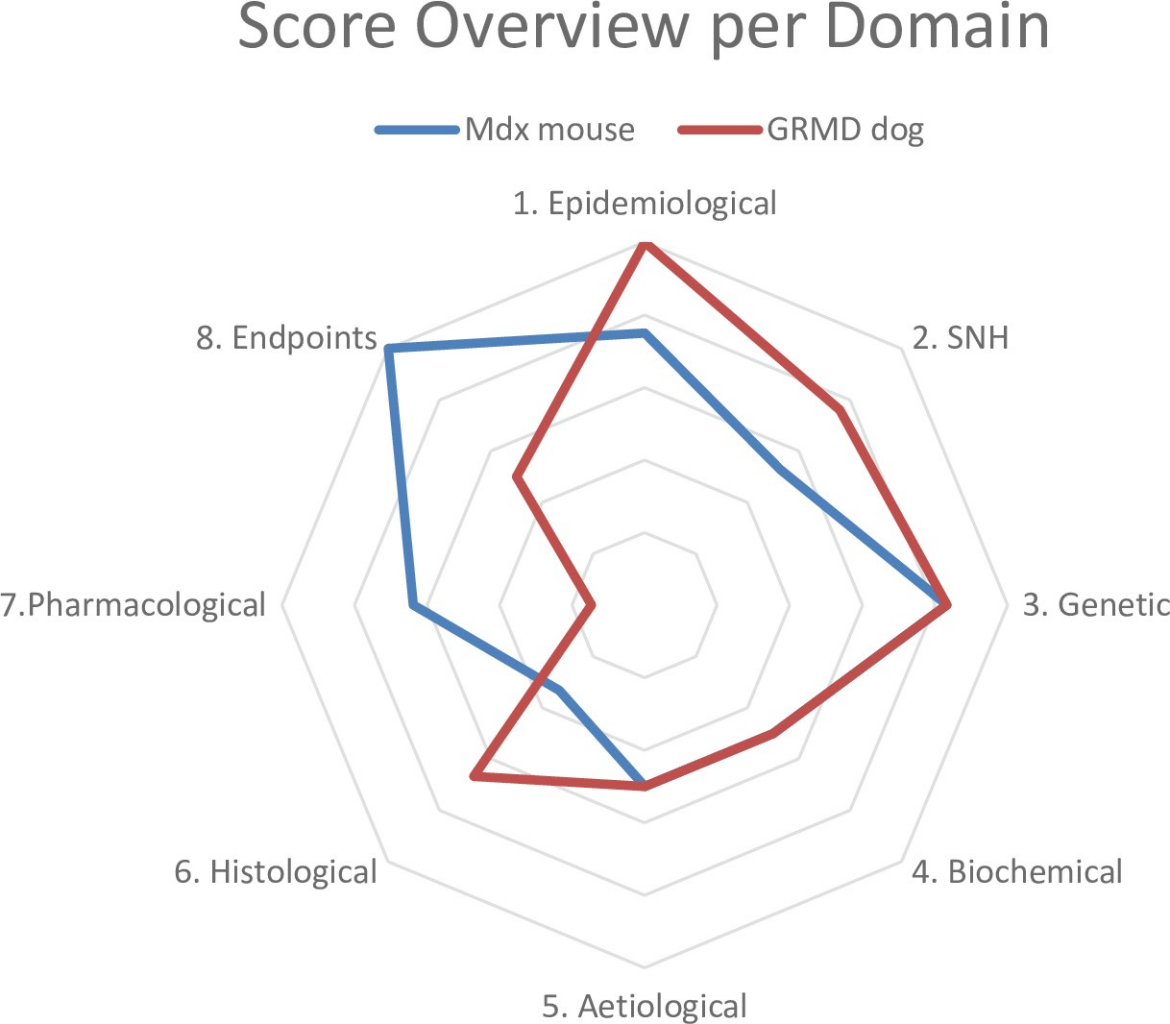
DMD models results. Radar plot with the scores per parameter per model of DMD. The closer a parameter is to the edge, the better the model simulates that aspect of the human disease.

A total of 58 articles were included for the mdx mouse and 41 for the GRMD dog. The final scores were 67.78 and 65.42 for the mdx mouse and GRMD dog, respectively. The relative scores per parameter are presented in Fig 3. The GRMD dog scores better in the epidemiological, SNH and histological domains while the mdx mouse does so in the pharmacological and endpoints domains. The GRMD dog mimics the natural history of the disease, symptoms (e.g. muscle wasting) and histopathological features (e.g. muscle regeneration) better than the mdx mouse. Especially for drugs which aim to slow down muscle degeneration or delay the disease onset, the GRMD dog is likely to generate more translatable data than the mdx mouse.

The difference in the pharmacological and endpoints domains stems mostly from the uncertainty factor, which is 18.2% and 6.1% for the GRMD dog and the mdx mouse respectively. Since most drug screening studies are done in mdx mice, there are more studies available for the Pharmacological Validation. However, there are no published studies in GRMD dogs for most drugs tested in humans. The only published study that assessed a functional outcome did so in sedated dogs, which reduced the score of the Endpoints Validation. Hence, a comparison between these models in these two domains is unlikely to be informative.

**Box 4. Reporting quality and internal validity**

A reporting quality and risk of bias assessment was included based on parameters adapted from the ARRIVE guidelines (see S2 Supporting Information, section F). Publications with experiments on two models were counted separately for each model. A total of 35 preclinical studies were included for the mdx mouse (32) and GRMD dog (3). Assessments of reporting quality and risk of bias per publication for the two models are provided in S7 Supporting Information. Table 4 shows the aggregated data for reporting quality and Table 5 for the risk of bias. Most preclinical studies did not report important measures to reduce the risk of bias, such as any mention of randomisation (68.6%) and blinding (57.1%). Even studies which did mention these measures to the design did not detail them enough to allow a proper evaluation of the risk of selection, performance and detection biases. The selective outcome reporting was the only parameter in which most studies were graded with a low risk of bias, indicating all outcomes defined in the methodology were reported in the results section. These findings are in line with previous literature, which puts the reliability of these experiments into question [4,5,7,21,23].

Male animals were more than two times more likely to be used than females while studies including both sexes accounted for almost one-third of the total (31.8%). Although in DMD this does not have a significant impact due to most patients with DMD being males, this trend is consistent with our findings for T2D which shown an overrepresentation of males in non-human animal studies (see S4 and S8 Supporting Informations files).

**Table 4:** Percentage of studies complying with the reporting quality parameters adapted from the ARRIVE guidelines per parameter per model.

Parameter	Mdx Mouse (N = 32)	GRMD dog (N = 3)	Total (N = 35)	Parameter	Mdx Mouse (N = 32)	GRMD dog (N = 3)	Total (N = 35)
	Y (%)				Y (%)		
Type of Facility	6.3	0.0	5.7	Environmental Enrichment	0.0	0.0	0.0
Type of Cage or Housing	12.5	0.0	11.4	Any Blinding	43.8	33.3	42.9
Bedding Material	0.0	0.0	0.0	Any Randomisation	34.4	0.0	31.4
Number of Cage Companions	12.5	0.0	11.4	Sample Size	84.4	100.0	85.7
Breeding Programme	53.1	100.0	57.1	Sample Size Calculation	6.3	33.3	8.6
Light/Dark Cycle	25.0	0.0	22.9	Acclimatisation	18.8	0.0	17.1
Temperature and Humidity	3.1	0.0	2.9	Sex Disclosed	46.9	33.3	45.7
Quality of the Water (fish)	-	-	-	Male/Female/Both (N = 15/1/16)	53.3/20.0/26.7	0/0/100	50.0/18.8/31.3
Type of Food	6.3	0.0	5.7	Background Control	-	-	-
Access to Food and Water	34.4	0.0	31.4	Background Model	-	-	-

**Table 5:** Percentage of studies with low (Yes) or unclear (Unclear) risk of bias per parameter per model.

Risk of Bias	Mdx Mouse (N = 32)	GRMD dog (N = 3)	Total (N = 35)
	Yes/Unclear (%)		
Allocation Concealment	0/100	0/100	0/100
Blinded Outcome Assessment	3.1/96.8	0/100	2.9/97.1
Blinded Operations	0/100	0/100	0/100
Random Cage Allocation	0/100	0/100	0/100
Random Outcome Assessment	0/100	0/100	0/100
Sequence Generation	0/96.8	0/100	0/97.1
Baseline Characteristics	3.1/96.8	0/100	2.9/97.1
Incomplete Outcome Data	18.8/67.7	0/100	17.1/71.4
Selective Outcome Reporting	96.9/3.2	100/0	97.1/2.9
Other	71.9/6.5	33.3/0.0	68.6/5.7

## Limitations

In FIMD, the validation of an animal model relies on the definition of the disease parameters and therefore, it depends on how well-understood the aetiology and pathophysiology are. Poorly understood diseases (e.g. Alzheimer’s) represent a major challenge for the definition of these parameters and thus to the validation of animal models in these indications. However, FIMD provides researchers with a platform to discuss both the more established aspects of a disease as well as the ones in which there is no consensus in a structured and standardised manner (e.g. aetiology, genetic, biochemical). This way, an animal model’s ability to simulate the different aspects of disease according to various theories or hypotheses (whenever available) can be discussed and assessed in one place by the same parameters.

All in all, FIMD provides researchers with the information necessary for them to decide on whether a model is the most relevant to test the efficacy of a new drug. Additionally, the definition of the disease parameters themselves might prove controversial, as there is currently no standardised and objective way of defining them. The recommendation to organise a task force with experts in the field to achieve consensus on which parameters to include could preclude the introduction of most biases, although it will frequently be unfeasible.

The weighting system provided here aims to roughly indicate in quantitative terms how well a model can simulate the human disease, but it could not be validated yet. Ideally, the weight of each question should be set based on a statistical model that correlates the likelihood of an animal model predicting human response with its assessment through FIMD. This approach would first require that a considerable number of models are assessed through FIMD; followed by an evaluation of the likelihood each model has of predicting efficacy in humans for each drug that has been tested on it. Then, it would be possible to correlate the likelihood of a model predicting human response with each type of answer to the validation questions. This statistical model would give a weighting for each question and also allow the setting of the weights specific to each indication, which would provide a more accurate and scientifically sound weighting system. Such an approach will become feasible as more animal models are validated using FIMD. Furthermore, the use of partial grades (50% of the score) for models which only partially mimic a particular aspect of the disease considerably decreases sensitivity, leading to the same score for models that simulate the same aspect to different extents.

Another limitation is that FIMD only includes publicly available information, which means it inherently incorporates the publication bias often reported in preclinical and clinical research [4,43,44]. However, there is a growing demand for the pre-registration of preclinical studies and the publication of their results [45–47]. With the establishment of registries like PreclinicalTrials.eu, the overall publication bias is expected to be reduced and therefore, so will be its impact on FIMD [48]. This factor is especially relevant for the Pharmacological Validation questions 7.1 and 7.2, which include drug intervention studies and might lead to skewed results. Also, due to time and resource constraints, we did not investigate the inter-rater reliability to verify the reproducibility of the results. However, we provide detailed instructions in S2 Supporting Information which are likely to reduce variation between users.

## Applications and final considerations

Based on a drug’s mechanism of action, models can be first discriminated by assessing whether the correlation between non-human animal and human drug studies of relevant pathways is available in the Pharmacological Validation domain. The other domains provide additional information, such as the presence of relevant genes and biomarkers. Finally, the validation level is an index of the reliability of a model’s overall ability to mimic the human condition, serving as another layer to further differentiate potentially more useful from less useful models. The combination of all these features allows researchers to select, among a plethora of models, the model most likely to correctly predict the efficacy of a drug in humans.

FIMD allows the comparison between animal models from a purely scientific perspective. In practice, other factors may influence the choice of one model over another, such as personnel expertise, available facilities and cost. Larger models (e.g. GRMD dog) are considerably more expensive than rodents (e.g. mdx mouse) and, despite possibly mimicking the human disease to a greater extent, might still not be the model of choice in many occasions. These factors are not taken into account in FIMD, as they vary not only between but also within institutions. However, by providing the scientific background on which to base the selection of a model, we hope to substantiate discussions within ethics committees and funding bodies on how these non-scientific factors can affect model selection. This discussion can provide researchers with arguments to obtain more funding to conduct projects on more expensive models which also have a higher likelihood of predicting the efficacy of new drugs.

An essential application for FIMD is on the approval of non-human animal studies by Institutional Review Boards (IRBs). IRBs often base their decisions on unpublished non-human animal studies with low internal validity [47]. FIMD presents an opportunity to assess the validity of a specific model for efficacy assessment while also providing insights from earlier research. By using the validation sheet of models used to support the first-in-human trials, IRBs can, for the first time, tackle all these issues at once. FIMD provides the background for the choice of the model(s), allowing IRBs to accurately assess whether the data generated is likely to be translatable to the clinic.

Although it is not our goal to address the problems with internal validity of animal studies with FIMD, we included a reporting quality and risk of bias assessment in the Pharmacological Validation. We aimed to confront researchers with the often poor quality of studies routinely used to base grant and marketing authorisation decisions, and subsequent research, forcing them to think about their own study designs. For this reason, we did not provide guidance on how to interpret these results as the relevance of each quality aspect will depend on the research question and indication.

The collection of validation sheets of models of efficacy will allow the development of an open database of validated models in which users can, based on their drug’s characteristics (e.g. mechanism of action or intended indication), find the optimal model to evaluate the efficacy of a drug before planning an animal experiment.

FIMD can simplify the interaction between companies and regulatory agencies as it permits a more objective and science-based discussion on the selection of an animal model. It can potentially prevent efficacy studies on less relevant models, effectively contributing to the reduction of the use of animal models in the context of the 3R’s. By assessing the degree to which a model mimics a human condition, FIMD facilitates the choice of an appropriate model for efficacy assessment and promotes the conduct of efficacy studies whose results are more likely to translate to the clinical situation.

## Supporting information

S1 Supporting Information Survey. (DOCX)

S2 Supporting Information Instructions. (DOCX)

S3 Supporting Information FIMD template. (DOCX)

S4 Supporting Information Pilot Study T2D. (DOCX)

S5 Supporting Information Validation DMD. (DOCX)

S6 Supporting Information PV Secondary assessments–T2D. (XLSX)

S7 Supporting Information PV Secondary assessments–DMD. (XLSX)

S8 Supporting Information Score calculator. (XLSX)
