# Correction: A standardised framework to identify optimal animal models for efficacy assessment in drug development

**DOI:** 10.1371/journal.pone.0220325

**Published:** 2019-07-22

**Authors:** Guilherme S. Ferreira, Désirée H. Veening-Griffioen, Wouter P. C. Boon, Ellen H. M. Moors, Christine C. Gispen-de Wied, Huub Schellekens, Peter J. K. van Meer

The first author's initials appear incorrectly in the citation. The correct citation is: Ferreira GS, Veening-Griffioen DH, Boon WPC, Moors EHM, Gispen-de Wied CC, Schellekens H, et al. (2019) A standardised framework to identify optimal animal models for efficacy assessment in drug development. PLoS ONE 14(6): e0218014. https://doi.org/10.1371/journal.pone.0218014

## References

[1] S. Ferreira G, Veening-Griffioen DH, Boon WPC, Moors EHM, Gispen-de Wied CC, Schellekens H, et al (2019) A standardised framework to identify optimal animal models for efficacy assessment in drug development. PLoS ONE 14(6): e0218014 10.1371/journal.pone.0218014 31194784 PMC6563989

