# Supplementary material for: A standardised framework to identify optimal animal models for efficacy assessment in drug development
Source: PLoS One. 2019 Jun 13;14(6):e0218014. doi: 10.1371/journal.pone.0218014 (PMC6563989; doi:10.1371/journal.pone.0218014)
Supplement: S1 Supporting Information — (DOCX) [file pone.0218014.s001.docx]

# Supporting Information S1 – Survey

# A. Survey Results

A web-based survey was created to collect feedback regarding the use and implementation of FIMD (formerly Question-Based Validation Sheet - QBVS) from experts from different stakeholders (e.g. regulatory agencies, academia and pharmaceutical industry). The expert panel had access to the questionnaire, instructions on how to complete it and the results of the case studies. Their suggestions were processed, considered relevant and therefore, implemented in FIMD.

The anonymous survey could be accessed via an open link, which means any person with the link could access it. The link was initially sent by email to 46 experts, which were identified by convenience sample (i.e. use of authors’ network, contacting corresponding authors of studies included in the pilot study). The distribution of the survey link within the experts’ network was not only allowed but also encouraged. Nonetheless, due to the low number of responses, only descriptive statistics are presented.

Twelve (12) experts participated in the survey. The majority is based in Europe (83%), are academics (67%), but industry and regulatory agencies were also represented (25% and 8%, respectively). All experts had experience with designing animal studies, 92% have conducted animal experiments, 83% have assessed and reviewed animal studies, 67% have developed or optimised animal models and 25% have experience on evaluating the risk of bias and publication bias, drafting guidelines for safety aspects or regulatory aspects in general. Most respondents (75%) have at least ten years of experience in these functions, 17% have at least five and 8% between two and five years.

Most respondents support the use of FIMD as a tool to validate animal models of disease (92%). Around 83% responded it is only moderately likely for two independent researchers to arrive at the same results, despite the availability of instructions on how to fill in and score each question. The main reason reported was the difference in background and the individual skill of each researcher.

Regarding adoption, almost 42% of those surveyed answered that is ‘slightly likely’ for FIMD to be adopted in their organisation, followed by 33% answering it is ‘very likely’ while 27% thinks it is only ‘moderately likely’. The main reasons reported are the significant time investment for training and execution of the validation.

As for the score, most experts think it adds value to the validation process (83%), although 17% sees it also as a potential source of bias. More than half affirmed that the score should be defined only in the method (58%), not allowing researchers to set the weights themselves.

One respondent commented that, for some animal models, there are significant differences in pathophysiological parameters between colonies, which should be reported. One example is the GRMD dog, which has a different natural history of the disease in some colonies: in one American colony, dogs hardly ever lose ambulation while in the French colony, around 25% lose the ability to walk (1). If the phenotype differences are substantial enough, they shall be investigated beforehand, and, in such cases, each colony would be considered as a related, but an individual model. In this situation, only studies which included animals from that specific colony shall be included. These differences shall also be reported in the ‘Background Information’ section of FIMD for all related models.

Another respondent suggested that a parameter should be included to indicate how many articles support the answers in each section of FIMD. This parameter was called ‘strength of evidence’: the more well-established the evidence becomes, the higher the strength of evidence. We decided not to include it because to make it useful; we would have to apply it to all questions, unreasonably increasing the complexity of the framework and hampering its feasibility.

An updated to the weighting system was also suggested by the expert panel. Where FIMD initially supported a one-size-fits-all approach to defining the weighting, the development of a fit-for-purpose weighting system was recommended to improve the adoption of FIMD, especially in the industry. Such a strategy aimed at increasing the resolution of the scoring system. Nonetheless, since there are not enough data to determine the values accurately, we have not been able to test whether the different weighting systems, in fact, increase resolution. At this stage, the presence of such a feature is hardly justifiable, given the complexity it adds to the overall framework. Thus, we decided not to include the indication-specific weighting. We have maintained only the same weighting system, in which all domains (and questions within a domain) have the same weighting.

# B. Web-Survey Questions

**Q0 Which of the examples below is clearer in helping to understand how to answer the following question: 2.1.2 Is the model able to simulate the disease in the relevant age groups (juvenile, adult or ageing)?**

1. Specific Example

Yes, completely.

The ZDF rat develops diabetes around the same age as humans – middle-aged adults, progressing into old age and death.

Yes, partially.

Although the time to onset of diabetes has been decreasing in the past years, ZDF rat still develops diabetes somewhat earlier than humans. However, it progresses into adulthood and ageing phases, similarly to the human disease.

No.

The ZDF rat develops diabetes much earlier than humans already in their first two weeks. The disease progresses and leads to death before the beginning of adulthood.

2. Generic Example

Yes, completely.

The model X develops disease Y around the same age as humans – middle-aged adults, progressing into old age and death.

Yes, partially.

Although the time to onset of disease Y has been decreasing in the past years, the model X still develops disease Y somewhat earlier than humans. However, it progresses into adulthood and ageing phases, similarly to the human disease.

No.

The model X develops disease Y much earlier than humans already in their first two weeks. The disease progresses and leads to death before the beginning of adulthood.

**Q11 Based on the instructions provided and your own experience, would you be able to fill out the QBVS for a given animal model?**

1. Yes

2. No

3. I am not sure

**Q111 What would prevent you from being able to fill out the QBVS?**

1. Questions not clear enough

2. Instructions not clear enough

3. Instructions not objective enough

4. Other

**Q12 If different people fill out the QBVS for the same model, how likely is it that they would arrive at the same results?**

1. Not at all likely

2. Slightly likely

3. Moderately likely

4. Very likely

5. Completely likely

**Q121 Why do you think they would not arrive at the same results?**

Open text.

**Q13 Do you think the QBVS can be used as a tool to validate animal models?**

1. Yes

2. No

**Q131 Why do you think the QBVS cannot be used as a tool to validate animal models?**

Open text.

**Q14 Would you include, edit or exclude any questions?**

1. Yes

2. No

**Q141 Which changes would you suggest?**

Open text.

**Q21 Do you think having a score included in the QBVS...**

1. adds value to it?

2. introduces bias?

3. Other

**Q211 How would you determine the weight for each question?**

Open text.

**Q212 For what reason(s) would it not add value?**

Open text.

**Q213 For what reason(s) would it introduce bias?**

Open text.

**Q22 Having a score included in the QBVS would affect my opinion of it…**

1. negatively

2. positively

3. neither negatively nor positively

**Q23 If the QBVS were to have a score included, do you think the weighting system(s)...**

1. should be defined only in the QBVS method

2. should be defined only by the person filling out the QBVS

3. should be defined in the QBVS method and also allow the person filling out the QBVS to set its own

4. I do not think QBVS should have a score embedded

5. none of the above

**Q31 From the presented weighting methods, which represents the best way to weight different aspects of disease simulation in animal models?**

1. SW

2. W1

3. W3

4. None of the above.

5. I do not know.

**Q32 Would you make any changes to the weights of specific questions?**

Open text.

**Q41 What is the likelihood of the QBVS tool being adopted by you and your colleagues?**

1. Not at all likely

2. Slightly likely

3. Moderately likely

4. Very likely

5. Completely likely

**Q411 Why is it unlikely you and your colleagues would adopt the QBVS?**

1. the time investment is too big

2. the instructions are not clear enough

3. the method is not objective enough

4. too much training required

5. other

**Q412 What changes should be made for you to adopt the QBVS?**

Open text.

**Q51 Do you have any additional remarks not covered by any of the previous questions?**

Open text.

**Q52 Would you know anyone who could be also interested in participating in this consultation? If so, please leave their name and email for contact below.**

Open text.

**Q61 In which region are you located?**

1. USA and Canada

2. Latin America

3. Europe

4. Africa

5. Middle East

6. Asia

7. Australia

**Q62 What is your position at your institution?**

Open text.

**Q63 For what kind of institution do you work?**

1. Government Body

2. Industry (1-10 employees)

3. Industry (11-50 employees)

4. Industry (51-250 employees)

5. Industry (251+ employees)

6. Regulatory Agency

7. Research Institute (not within a University)

8. University

9. Other

**Q64 In which activities presented below are or have you been involved? (mark all that apply)**

1. Design of animal studies

2. Conduct of animal studies

3. Animal model development and/or optimisation

4. Assessment and review of animal studies (including due diligence)

5. Other

**Q65 How many years of experience do you have in these functions?**

1. Up to 2 years

2. 2+ to 5 years

3. 5+ to 10 years

4. 10+ years.
