# Supplementary material for: A standardised framework to identify optimal animal models for efficacy assessment in drug development
Source: PLoS One. 2019 Jun 13;14(6):e0218014. doi: 10.1371/journal.pone.0218014 (PMC6563989; doi:10.1371/journal.pone.0218014)
Supplement: S2 Supporting Information — (DOCX) [file pone.0218014.s002.docx]

# S2 Supporting Information - Instructions

# A. Getting Started

The definition of the validation parameters is the first step to validate an animal model in a specific indication. While we recommend a task force of experts in the field as the most objective and reliable alternative to define these parameters, we realise the difficulties to set one up will often prevent its implementation. In these cases, the disease parameters can be identified by searching for published reviews on the disease and its natural history. Only full-text articles in English and published in peer-reviewed journals shall be included.

To identify the correct terminology for both the indication and model of interest, we suggest the use of the Medical Subject Headings (MeSH) website^[[1]](#footnote-2)^ for PubMed; and the Emtree^[[2]](#footnote-3)^ for Embase. For animal models, it is important to include abbreviations and plurals as well (*"ZDF rat" OR "ZDF rats" OR "Zucker Diabetic Fatty rat" OR "Zucker Diabetic Fatty rats"*). Besides reviews published in the literature, typing the strain of the model (e.g. BKS.Cg-Dock7m +/+ Leprdb/J for the db/db mouse) on the supplier’s website (e.g. Jackson Laboratories, Charles River Laboratories) often provides extra information on the basic characterisation of the strain. Also, whenever different background strains can be used to breed the model, it shall be investigated whether there are significant differences between different strains. If so, separate validation sheets must be filled for each background strain of interest. If the background strain is not mentioned, it shall be reported as such.

Once relevant reviews are identified, the reference list and forward citation (e.g. Google Scholar, Web of Science, Scopus) shall be used to determine original research to answer the validation questions. Since completing the questionnaire is mostly an iterative process, no specific search string is needed to answer most questions, except for the Pharmacological Validation questions 7.1 and 7.2, for which further guidance is provided in their respective sections. In the case that after the general literature search some questions are still left unanswered, specific search strings can be used to identify relevant articles. For example, if no information was initially found about the up- or downregulation of a gene in a model, PubMed and Embase can be searched with a string such as ‘[model, including terminology variations] AND [gene]’. The use of MeSH and Emtree terms for each search component is encouraged.

The final score is based on the Same Weighting (SW) system. Each domain has the same weight in a total of 100 points (12.5 per domain). The 12.5 points are equally divided by the number of questions in each domain. Based on the answers inputted in the Score Calculator (see S8 Supporting Information), a radar plot will be automatically generated.

Once the final score, the uncertainty and similarity factors are calculated (sections D and E, respectively), fill in this information in the first page of the validation sheet. Include the validation date (latest date of publication of the validation references). Determine the validation level as defined in Table 3 from the main text. Describe the historical background of the model as described in section B. Fill each question’s score.

Below we present a quick stepwise approach to FIMD:

1. Use MeSH and Emtree to identify all terminology related to the model and indication of interest, including abbreviations and plurals;
2. Organise an expert group to define the disease parameters. If that is not possible, search for reviews on the human disease and animal models for that condition. Whenever necessary, justify the choice of disease parameters;
3. Answer the questions in the validation sheet template (S3 Supporting Information) based on the information found in the papers. If, in the end, some questions are still unanswered, use specific search strings for these questions;
4. For the Pharmacological Validation, assess each paper based on the instructions for questions 7.1, 7.2; and sections F1 Reporting Quality and F2 Risk of Bias. Fill this information in a separate spreadsheet file (such as S7 Supporting Information);
5. Fill in the Score Calculator (S8 Supporting Information) to get the total score and the radar plot;
6. Calculate the Uncertainty Factor according to section D;
7. If another model has been validated for the same indication, calculate the Similarity Factor according to section E;
8. Finalise the validation sheet (fill in the first page validation sheet, score per question).

# B. General Definitions

**Answers**

All questions (except for 7.1 and 7.2) shall be answered with ‘yes’, ‘yes, completely’, ‘yes, partially’, ‘no’ or ‘unclear’. Examples based on the results of the pilot study of Type 2 Diabetes (T2D) and the full validation of Duchenne Muscular Dystrophy (DMD) models are provided in the *How to score?* grey-lighted boxes to illustrate how the questions should be answered. Given the currently limited available dataset, it was not possible to provide data-driven examples for all possible answers to all questions. For each question, one answer was extracted as it is from S4 and S5 Supporting Information files and hypothetical answers were provided for all the other possible answers.

‘Yes’ and ‘Yes, completely’ have the same meaning and are equivalent. The difference in use depends on whether the question allows a gradation of response. For example, question 3.1 refers to the presence of orthologous genes and proteins in the model, for which the answer can only be ‘yes’, ‘unclear’ or ‘no’ since each gene is assessed individually. On the other hand, answering question 5.1 with a ‘yes’ is not enough as the question allows the gradation: animal models may only partially mimic the human condition’s aetiology. Questions answered with ‘Yes’ or ‘Yes, completely’ get 100% of the score.

‘Yes, partially’ is used to answer questions in which the model only recapitulates parts of the human disease parameter. For instance, if for question 1.1, a human condition can manifest in both males and females, but the animal model can only simulate the disease in males, the answer shall be ‘yes, partially’ as the model can only partially mimic that aspect. Questions answered with ‘Yes, partially’ get 50% of the maximum score.

‘No’ shall be used to answer questions when the model does not mimic that aspect of the human condition at all. For one question (1.1) we did not include a ‘no’ answer in the examples because such an answer would require an animal model to simulate the human condition in the opposite sex the condition manifest in humans, an unlikely situation. Questions answered with ‘No’ get 0% of the maximum score.

‘Unclear’ shall be used in two situations. The first is when no literature can be found even after the use of specific search strings. The second is to answer questions for which conflicting evidence is found. For example, if when answering question 3.3 two papers are found: one stating there is no change in a gene’s regulation while another shows upregulation of said gene, this characterises conflicting evidence. A brief discussion on the results available is warranted, which shall also include an assessment of whether one or another is more likely to be true if possible. Special attention is necessary in cases of old vs new studies, in which new evidence might be brought to light which contradicts previous research. Whenever possible, these differences must be investigated to evaluate whether there is a new consensus in the field that previous data is not considered accurate any longer. We encourage researchers to always search for recent papers to further corroborate old research findings (e.g. up to the 2000s). Questions answered with ‘Unclear’ get 10% of the maximum score.

**Well-established**

The term ‘well-established’ is used in some questions to indicate a scientific consensus on a topic. Its subjectivity is intentional, and it requires a scientifically sound and well-referenced justification.

# C. Fields Breakdown

**Model Name**

The name of the model shall be presented with as much detail as possible, including specific genetic alterations whenever relevant (e.g. Zucker Diabetic Fatty (ZDF)-*Lepr^fa^*/Crl rat). In case a model has a much commoner name, this shall be stated in parenthesis (e.g. C57Bl10scsn-Dmdmdx mouse (mdx mouse)).

**Indication**

The indication for which the model is being validated shall be stated. In case of symptoms of diseases, the disease in which the chosen symptom is manifested shall also be stated (e.g. food poisoning diarrhoea).

**Validation Date**

The validation date refers to the publication date of the last peer-reviewed reference included in any of the eight domains.

**Subsections**

Subsections can either be questions for which no further distinction is necessary (e.g. 5.1 on aetiology) or each section within a question (e.g. in question 7.1 on effective drugs, each drug is considered a subsection). For instance, a specific indication had its parameters defined as 5 symptoms, 4 genes, 2 pharmacodynamic and 2 prognostic biomarkers, 4 histopathological features, 1 approved drug class with 2 drugs approved and 2 failed drug classes with one drug each. The total number of subsections would be: 2 (epidemiological) + 9 (SNH) + 12 (genetic) + 8 (biochemical) + 1 (aetiological) + 4 (histological) + 5 (pharmacological) + 2 (endpoints), which is equal to 43. This number is the denominator used to calculate the uncertainty and similarity factors (sections D and E, respectively).

**Total Score**

The total score is the sum of each question score for all eight domains. It is calculated automatically by the Score Calculator (S8 Supporting Information).

**Validation Level**

The validation level refers to the level of confidence in the reliability of a model to simulate the human condition, and it is defined based on the percentage of definite answers in the validation sheet. A ‘definite answer’ is defined as any answer except for ‘unclear’, which is used to indicate the absence of evidence in the literature or conflicting results. The validation level can be insufficiently validated (up to 40% of definite answers); slightly validated (41-60%); moderately validated (61-80%); and highly validated (81-100%).

**Historical Background**

A brief historical background of the model shall be presented. It shall include the first publication to mention the model whenever possible as well as a brief history line of its characterisation. Known colony and supplier variations shall be stated at the end with the relevant references.

**1 Epidemiological Validation**

**1.1** **Is the model able to simulate the disease in the relevant sexes?**

Describe whether the model can reproduce the disease in both sexes with sex-specific characteristics in a similar fashion to how the disease is manifested in humans. Sex-specific information on the pathophysiology of the disease shall be included if present (e.g. protective effect of oestrogen in females for the development of diabetes).

**How to score?**

*Yes, completely.*

Both male and female db/db mice can develop diabetes. The diabetic condition is somewhat more pronounced in male than in female mice. This specific female resistance to the development of diabetes is in line with what is seen in humans, due to a possible protective effect of oestrogen on pancreatic beta-cells.

*Yes, partially.*

Only male db/db mice can develop diabetes. Female mice have slightly higher but non-significant glycaemia and do not develop overt diabetes at any point in life.

**1.2 Is the model able to simulate the disease in the relevant age groups (e.g. juvenile, adult or ageing)?**

Describe whether the model can reproduce (spontaneously or by intervention) the disease in the relevant age groups (e.g. juvenile, adult and ageing) in which the disease is commonly manifested in humans (clinical presentation of symptoms). It is important to state in which age group is the onset of the disease in the model when compared to humans and whether the disease develops totally, partially or not at all in the same development phases. Where possible, narrower age categories shall be reported.

**How to score?**

*Yes, completely.*

The ZDF rat develops diabetes around the same age as humans – middle-aged adults, progressing into old age and leading to death.

*Yes, partially.*

Although the time to onset of diabetes has been decreasing in the past years, the ZDF rat still develops diabetes somewhat earlier than humans. However, it progresses into adulthood and ageing phases, similarly to the human disease.

*No.*

The ZDF rat develops diabetes much earlier than humans already in their first two weeks. The disease progresses and leads to death before the beginning of adulthood.

**2 Symptomatology and Natural History (SNH) Validation**

**2.1 Is the model able to replicate the symptoms and co-morbidities commonly present in this disease? If so, which ones?**

Describe whether the hallmark symptoms and co-morbidities of the disease are present and how well (completely, partially or not at all) the model can simulate these symptoms/co-morbidities. The definition of which symptoms/co-morbidities to include shall ideally be done by a working group of experts in the field. Alternatively, a search on scientific literature databases such as PubMed and Embase can be performed to identify reviews on the human condition from which the characteristic symptoms and co-morbidities can be identified. A brief description of each symptom in the model, when compared to the human condition, shall be added. Sometimes, human symptoms cannot be directly translated to the animal situation or are a result of a more fundamental process that can be compared in this section instead of the nominal symptoms. For instance, in T2D, the symptoms presented by humans (e.g. polyphagia, polydipsia, polyuria, weight loss) are directly caused by hyperglycaemia, hyper and hypoinsulinemia, and dyslipidaemia. We considered the presence of the latter three together with obesity – which is an important co-morbidity – as more relevant parameters to validate the animal models for T2D. In such cases, the justification must be presented in this section before the comparison between animal and human parameters.

**How to score?**

The total score for this question is calculated based on the proportionality of symptoms modelled and partially modelled. For example, if there are three symptoms defined for a given disease and a model simulates one symptom completely (whole point), one partially (half a point) and the last one not at all, the score would be calculated by multiplying 1.5/3 by the weight of this question.

**Examples**

*Symptoms modelled.*

Muscle wasting: comparable to DMD boys across all main groups of muscles.

*Symptoms partially modelled.*

Muscle wasting: comparable to DMD boys only regarding diaphragm degeneration.

*Symptoms not modelled.*

Muscle wasting: the mdx mouse does not have severe muscle wasting in any muscle as seen in humans.

**2.2 Is the natural history of the disease similar to human’s regarding:**

**2.2.1 Time to onset;**

Describe whether the model manifests the disease at around the same phase of development a human would. It is necessary to reference literature that compares the human and the species’ development, which is available for the most prevalent animal model species, such as mice, rats and dogs.

**How to score?**

*Yes.*

Although GRMD dogs have a somewhat high rate of neonatal death (~25%) not seen in either mdx mice or humans, the first clinical signs appear at 6-9 weeks, analogous to humans at 2-4 years.

*No.*

The first clinical signs appear at 15-20 weeks, much later than humans at 2-4 years.

**2.2.2 Disease progression;**

Describe whether the disease progresses in the model at around the same rate and leading to similar outcomes as in humans. The natural history of the disease shall be described comparatively in humans and the animal model, including the time to onset of different symptoms and complications.

**How to score?**

*Yes, completely.*

In humans, first symptoms start around 2.5 years, with loss of ambulation around 10 to 15 years. In GRMD dogs, muscular atrophy along with joint involvement (including jaw mobility), abnormal gait, decreased respiratory function and cardiomyopathy appears around six months of age. Like DMD, there is a honeymoon phase between 6 and ten months wherein the disease is relatively stable. Loss of ambulation is frequent, and like humans, death is commonly caused by heart failure and/or cardiomyopathy.

*Yes, partially.*

In humans, first symptoms start around 2.5 years, with loss of ambulation around 10 to 15 years. In GRMD dogs, muscular atrophy along with joint involvement (including jaw mobility), abnormal gait, decreased respiratory function and cardiomyopathy appears around six months of age. Like DMD, there is a honeymoon phase between 6 and ten months wherein the disease is relatively stable. Loss of ambulation is infrequent, with only around 1/3 of GRMD dogs losing ambulation completely, although they do lose mobility considerably.

*No.*

In humans, first symptoms start around 2.5 years, with loss of ambulation around 10 to 15 years. In GRMD dogs, muscular atrophy along with joint involvement (including jaw mobility), abnormal gait, decreased respiratory function and cardiomyopathy appears later in life, at around two years of age. There is no honeymoon phase wherein the disease is relatively stable. Loss of ambulation is infrequent, with only around 1 in 20 GRMD dogs losing ambulation completely, with most dogs being mobile until death.

**2.2.3 Duration of symptoms;**

Describe whether the symptoms are manifested for the same duration as the ones in humans (e.g. lifelong or temporary).

**How to score?**

*Yes, completely.*

A: Symptoms of DMD are lifelong due to permanent muscle degeneration. In mdx mice, once symptoms get worse, there is also permanent damage that leads to reduced lifespan.

*Yes, partially.*

A: Symptoms of DMD are lifelong due to permanent muscle degeneration. In mdx mice, once symptoms get worse until around 10 to 15 weeks, in which muscles sustain significant damage but do not further degenerate allowing some retention of function.

*No.*

A: Symptoms of DMD are lifelong due to permanent muscle degeneration. In mdx mice, symptoms get worse until around 10 to 15 weeks, in which muscles start to recover and partially regain their function.

**2.2.4 Severity**.

Describe whether the severity of the symptoms manifested in the model is similar to the ones manifested in humans. This shall be done comparatively, with a brief description of both the human and animal situation.

**How to score?**

*Yes, completely.*

A: Mdx mice show the same level of muscle degeneration as seen in humans, including great variation in fibre size, architecture and continuous necrosis and proliferation of connective tissue.

*Yes, partially.*

A: Mdx mice do not show the same level of muscle degeneration as seen in humans, except for the diaphragm which shows progressive degeneration evident at six months of age with great variation in myofibre size, architecture and continuous necrosis and proliferation of connective tissue.

*No.*

A: Mdx mice do not show the same level of muscle degeneration as seen in humans in any muscle.

**3 Genetic Validation**

**3.1** **Does this species also have orthologous genes and/or proteins involved in the human disease?**

Describe whether the primary genes and proteins involved in the pathophysiology of the disease in humans are present in the animal model species. Identification of such genes shall ideally be made by a working group of experts in the field. Alternatively, it can be done by searching for reviews on the genetic background of the disease and genome-wide association studies (GWAS). Once identified, the reasoning for such definition shall be justified with scientific literature. Additionally, for each identified gene, a brief description of its function and role in the pathophysiology of the disease along with its GeneID and location shall be reported for both humans and model species. The GeneID and location can be found at PubMed’s database, available at <https://www.ncbi.nlm.nih.gov/gene>.

**How to score?**

For each relevant gene/protein, a subsection shall be created and individually analysed. The total score for this question shall be calculated by the sum of each subsection (which can be answered according to the pre-specified answers).

**Examples**

*Yes.*

3.1.1 Dystrophin: the human dystrophin gene (Gene ID: 1756) is the largest gene found in humans measuring a total of 2.4 Mb located at Xp21.2-p21.1. Dystrophin forms parts of the dystrophin-glycoprotein complex (DGC), responsible for connecting the inner cytoskeleton and the extracellular matrix. In dogs, the dystrophin gene (Gene ID: 606758) is located at chromosome X.

*No.*

3.1.1. The dystrophin gene together with its protein product is not present in GRMD dog muscles. The human dystrophin gene (Gene ID: 1756) is the largest gene found in humans measuring a total of 2.4 Mb located at Xp21.2-p21.1. Dystrophin forms parts of the dystrophin-glycoprotein complex (DGC), responsible for connecting the inner cytoskeleton and the extracellular matrix.

**3.2 If so, are the relevant genetic mutations or alterations also present in the orthologous genes/proteins?**

Describe the specific alterations (mutations, deletions etc.) in those genes and proteins present in humans and check whether they are also present in the model. Relevant genetic alterations (mutations, deletions, rearrangements etc.) shall be included for each gene identified in question 3.1. The procedure to identify such alterations follows the same principles, in which the identification of GWAS plays a more prominent role. A brief description of the effect of each alteration shall be included for each gene, contextualising it for both humans and the animal model.

**How to score?**

For each relevant gene/protein, a subsection shall be created and individually analysed. The total score for this question shall be calculated by the sum of each subsection (which can be answered according to the pre-specified answers).

**Examples**

*Yes, completely.*

A: DMD is caused by deletions, duplications, small mutations or other smaller rearrangements of the gene that codes for dystrophin in chromosome X. In that sense, DMD being an X-linked inherited disorder, the mdx mouse has the same aetiology (spontaneous mutations). This is caused by a nonsense point mutation (C-to-T transition) in exon 51 that aborts full-length dystrophin expression, which is the case 13% human patients and is the site of action of eteplirsen, an exon-skipping drug approved for the treatment of DMD.

*Yes, partially.*

A: DMD is caused by deletions, duplications, small mutations or other smaller rearrangements of the gene that codes for dystrophin in chromosome X. In that sense, DMD being an X-linked inherited disorder, the mdx mouse has the same aetiology (spontaneous mutations). Nevertheless, mdx mice have a nonsense point mutation (C-to-T transition) in exon 23 that aborts full-length dystrophin expression. This is not the case for human patients who have other alterations that lead to dystrophin deficiency.

*No.*

A: DMD is caused by deletions, duplications, small mutations or other smaller rearrangements of the gene that codes for dystrophin in chromosome X. The mdx mouse does not have the same mutation, as the lack of dystrophin in the muscles is caused by a deletion located at chromosome 2, completely different from the human situation.

**3.3 If so, is the expression of such orthologous genes and/or proteins similar to the human condition?**

Describe whether the expression (under-, normal or overexpression) levels of the relevant genes and proteins mentioned in the questions above and check whether the model has the same pattern of expression. If relevant, temporal (i.e. relevant only during a specific stage of development of disease phase) and spatial (i.e. present only in specific tissues) aspects of expression must also be considered.

**How to score?**

For each relevant gene/protein, a subsection shall be created and individually analysed. The total score for this question shall be calculated by the sum of each subsection (which can be answered according to the pre-specified answers).

**Examples**

*Yes.*

3.3.1 Dystrophin: Like DMD patients, mdx mice are dystrophin-deficient not expressing full-length dystrophin.

*No.*

3.3.1 Dystrophin: Mdx mice are not dystrophin-deficient, expressing fully functional dystrophin.

**4 Biochemical Validation**

**4.1 If there are known pharmacodynamic (PD) biomarkers related to the pathophysiology of the disease, are they also present in the model?**

Describe whether the known and well-established PD biomarkers relevant to the pathophysiology of the disease in humans are also present in the model. Pharmacodynamic biomarkers are defined as molecules which can describe the current state of a disease. Pharmacodynamic biomarkers can also be prognostic biomarkers. Identification of such biomarkers shall ideally be done by a working group of experts in the field. Alternatively, reviews published in the literature can be used to identify these biomarkers. In both cases, scientific literature shall be included to support their choice. If relevant, temporal (i.e. relevant only during a specific stage of development of disease phase) and spatial (i.e. present only in specific tissues) aspects must also be considered.

**How to score?**

If more than one biomarker is identified, a subsection shall be created for each relevant biomarker and individually analysed. The total score for this question shall be calculated by the sum of each subsection (which can be answered according to the pre-specified answers).

**Examples**

*Yes.*

A: High levels of creatine kinase (CK) – a marker of muscle damage, with the muscle-type variants being predominant in plasma are also present in mdx mice.

*No.*

A: Creatine kinase (CK) levels – a marker of muscle damage, with the muscle-type variants being predominant in plasma are not elevated in mdx mice.

**4.2 Do these PD biomarkers behave similarly to humans’?**

Describe whether the behaviour of such biomarkers (higher or lower levels) is similar to the one seen in humans. If relevant, temporal (i.e. relevant only during a specific stage of development of disease phase) and spatial (i.e. present only in specific tissues) aspects of expression must also be considered.

**How to score?**

If more than one biomarker is identified, a subsection shall be created for each relevant biomarker and individually analysed. The total score for this question shall be calculated by the sum of each subsection (which can be answered according to the pre-specified answers).

**Examples**

*Yes.*

Like humans, ZDF rats have increased levels of blood glucose.

*No.*

Unlike humans, ZDF rats have normal levels of blood glucose.

**4.3 If there are known prognostic biomarkers related to the pathophysiology of the disease, are they also present in the model?**

Describe whether the known and well-established prognostic biomarkers in humans are also present in the model. Prognostic biomarkers are defined as any measurement which can often predict the rate of progression of the disease and/or the likelihood of an outcome (e.g. recurrence). They can also be pharmacodynamic biomarkers. The identification of such biomarkers shall ideally be made by a working group of experts in the field. Alternatively, reviews published in the literature can be used to identify these biomarkers. In both cases, scientific literature shall be included to support their inclusion. If relevant, temporal (i.e. relevant only during a specific stage of development or disease phase) and spatial (i.e. present only in specific tissues) aspects must also be considered.

**How to score?**

If more than one biomarker is identified, a subsection shall be created for each relevant biomarker and individually analysed. The total score for this question shall be calculated by the sum of each subsection (which can be answered according to the pre-specified answers).

**Examples**

*Yes.*

A: Glycaemic markers are also prognostic markers as higher glycaemic levels can potentially lead to faster worsening of the diabetic condition. Glycaemic markers can also be measured in ZDF rats.

*No.*

A: Glycaemic markers are also prognostic markers as higher glycaemic levels can potentially lead to faster worsening of the diabetic condition. Unlike humans, no glycaemic markers can be measured in ZDF rats.

**4.4 Do these prognostic biomarkers behave similarly to humans’?**

Describe whether the behaviour of such biomarkers (higher or lower levels) is similar to the one seen in humans. If relevant, temporal (i.e. relevant only during a specific stage of development of disease phase) and spatial (i.e. present only in specific tissues) aspects of expression must also be considered.

**How to score?**

If more than one biomarker is identified, a subsection shall be created for each relevant biomarker and individually analysed. The total score for this question shall be calculated by the sum of each subsection (which can be answered according to the pre-specified answers).

**Examples**

*Yes.*

Like humans, ZDF rats have increased levels of blood glucose.

*No.*

Unlike humans, ZDF rats have normal levels of blood glucose.

**5. Aetiological Validation**

**5.1 Is the aetiology of the disease similar to humans’?**

Describe whether the aetiology of the disease in the model is similar to humans regarding both genetic and environmental (including lifestyle) factors. A brief review of what is known of the aetiology of the disease shall be included together with a comparative discussion on the animal model’s disease aetiology. Genetic factors shall be cited in reference to the Genetic Validation domain, and environmental factors shall also be described.

It can be the case that a disease’s aetiology is not yet known (idiopathic conditions) or there are many plausible causes (e.g. Alzheimer’s Disease). In these cases, a discussion must be provided on the human aetiology including all current theories and a comparison of the model to each of them.

**How to score?**

If the disease’s aetiology is not known or there are many concurrent hypotheses, since there is no ‘correct’ parameter to compare to, models which fit in one of the possible aetiologies shall be scored with a ‘Yes, partially’.

**Examples**

*Yes, completely.*

A: DMD is caused by deletions, duplications, small mutations or other smaller rearrangements of the gene that codes for dystrophin in chromosome X. In that sense, DMD is an X-linked inherited disorder, the mdx mouse has the same aetiology (spontaneous mutations). This is caused by a nonsense point mutation (C-to-T transition) in exon 51 that aborts full-length dystrophin expression, which is the case 13% human patients and is the site of action of eteplirsen, an exon-skipping drug approved for the treatment of DMD.

*Yes, partially.*

A: DMD is caused by deletions, duplications, small mutations or other smaller rearrangements of the gene that codes for dystrophin in chromosome X. In that sense, DMD is an X-linked inherited disorder, the mdx mouse has the same aetiology (spontaneous mutations). Nevertheless, mdx mice have a nonsense point mutation (C-to-T transition) in exon 23 that aborts full-length dystrophin expression, which is not necessarily the case for human patients who can have other mutations that may also lead to dystrophin deficiency.

*No.*

A: DMD is caused by deletions, duplications, small mutations or other smaller rearrangements of the gene that codes for dystrophin in chromosome X. The mdx mouse does not have the same mutation, as the lack of dystrophin in the muscles is caused by a deletion located at chromosome 2, completely different from the human situation.

**6. Histological Validation**

**6.1 Do the histopathological structures in relevant tissues resemble the ones found in humans?**

Describe whether the main histopathological features in target tissues in humans are also present in the model. Identification of such structures shall ideally be made by a working group of experts in the field. Alternatively, reviews describing the disease and the animal model commonly make a comparison between histopathological features and can be used to identify such structures. A brief description of the comparison between the human and the animal situation shall be included.

**How to score?**

The total score for this question is calculated based on the proportionality of histopathological features modelled and partially modelled. For example, if there are three histopathological features defined for a given disease and a model simulates one histopathological feature completely (whole point), one partially (half a point) and the last one not at all, the score would be calculated by multiplying 1.5/3 by the weight of this question.

**Examples**

*Histopathological feature modelled.*

Muscle regeneration: existent with similar intensity as humans, being unable to outpace the increasing muscle necrosis.

*Histopathological feature partially modelled.*

Muscle regeneration: existent but at a somewhat faster pace than in humans, considerably slowing down the increasing muscle necrosis.

*Histopathological feature not modelled.*

Muscle regeneration: existent but at a much faster pace than in humans, effectively countering muscle necrosis with almost no effect on function.

**7. Pharmacological Validation**

**7.1 Are effective drugs in humans also effective in this model?**

Describe whether drugs proven to be effective in humans are also effective in the model, which indicates that the underlying mechanisms of the disease present in humans are also present in the model. The objective of this domain is to compare the relevance of pathways knowingly involved in the human disease’s pathophysiology. Thus, different drug classes are used as examples to test the relevance of specific pathways (even if they overlap) in the model when compared to humans.

An effective drug is defined as a drug that has been determined to have a positive benefit-risk profile by the Committee for Medical Products for Human Use (CHMP), and thus received a Marketing Authorisation Application from the European Medicines Agency (EMA); or has been approved by the Food and Drug Administration (FDA). Drugs are grouped in classes by their mechanism of action and/or chemical similarity. Drug classes can be identified by searching for their Anatomical Therapeutic Chemical Classification System (ATC), publications of preclinical or clinical studies, reviews of management and/or therapy of the disease, consulting pharmacology textbooks and/or by accessing commercial drug databases such as Adis Insight and Pharmaprojects. All drugs approved for the treatment of a given indication shall be included. Inclusion of all drugs of each class allows a more systematic assessment of the pathway in the model for many reasons. The first is by preventing the introduction of bias by selectively including a drug which has positive or negative effects not shared by the rest of the class. Secondly, by skewing of results in case the model responds differently to a particular drug than to the rest of the class. Finally, by correcting for the fact that not all drugs of a class will be tested in all models.

The methodology (i.e. search string, database(s) searched, date of search and number of results found) to search for animal studies must be disclosed to allow reproducibility. Search strings for this question shall consist of the model’s name (including abbreviations and plurals) and a drug (including drug codes and alternative names), e.g. *("ZDF rat" OR "ZDF rats" OR "Zucker Diabetic Fatty rat" OR "Zucker Diabetic Fatty rats") AND (exenatide OR "AC 2993" OR "AC 2993 LAR" OR Bydureon OR Byetta OR "Ex4 Peptide" OR "Exendin 4" OR Exendin-4 OR "ITCA 650")*. In case too many hits are found, we advise to clean the results by adding [mesh], [tiab] to the model and drug search components.

Studies shall only be included if they include at least a control (placebo or an approved drug) and a monotherapy arm of the drug (combinations can be considered if they are the standard of care or if a combination drug is being developed). This does not apply to indications for which no approved treatment is available. Studies in which the treatment arm consists of plant extracts, phytotherapeutics and dietary supplements shall not be included unless they are extensively characterised. Studies shall be included only if the drug being tested is administered *in vivo* after the onset of the disease. Prevention studies (when a drug is administered before the onset of the disease) shall only be included if the indication aims to prevent the development of a condition. In case of Advanced Therapeutics Medicinal Products (ATMPs) or other drugs that are designed to interact specifically with human receptors or other structures, it is advised to also include in the search string a term for homologous drugs for that species. A summary of results shall also be included, highlighting the main effects of each drug primarily on the functional outcomes and secondarily on the other types of outcomes, according to the authors’ significance testing. Conflicting results shall be reported and briefly discussed.

Additionally, a secondary file shall be created to include the classification of each study according to the concepts of general outcome and outcome context as defined below (How to score? Questions 7.1 and 7.2). A reporting quality and risk of bias assessment shall also be performed according to the parameters defined in the Reporting Quality and Risk of Bias Assessment in section F. An example is provided in S7 Supporting Information.

**7.2 Are ineffective drugs in humans also ineffective in this model?**

This question aims to identify whether drugs proven to be ineffective in humans are also ineffective in the model, indicating that the underlying mechanisms by which these drugs act have either similar relevance or are not present neither in humans nor the model.

Ineffective drugs are defined as drugs that due to lack of efficacy have either failed in clinical trials – phase II or III or were withdrawn by either agency; and do not share a mechanism of action with any drug currently approved by either EMA or FDA for the same indication. They can be identified by searching clinicaltrials.gov for clinical trials for any given indication. With the list of drugs (and drug codes) currently in development, drugs that have failed at phase II or III due to lack of efficacy can be identified via literature search of clinical trials published and/or press reports from originator/licensee companies. Additionally, a literature search can also be conducted for the mechanism of actions of drugs identified this way, so that even if a drug does not have clinical trials registered at clinicaltrials.gov, it could still be identified through its mechanism of action. When using the mechanism of action in the string, it is advised to look for the specific medical subject headings (MeSH) in Pubmed or Emtree terms in Embase as they allow the search for all drugs already catalogued within that class. Alternatively, the use of commercial drug databases such as Adis Insight and Pharmaprojects, which offer specific filters for terminated projects, is also encouraged. Drugs which have been withdrawn by regulatory agencies due to lack of efficacy can easily be identified by searching EMA and FDA’s websites. Only drugs which have been tested in clinical trials that investigate efficacy shall be included.

This question follows the same reporting standards as question 7.1.

**How to score? Questions 7.1 and 7.2.**

The main findings of the included studies shall be reported in a brief description of each drug. The total score is calculated by the product of the weight of the pharmacological validation section by the sum of each subsection’s score. The latter is calculated by the product of the general outcome (G_O_), outcome context (O_c_) and subsection value (S_v_). All values shall be reported with four decimals. A formula for such calculation is provided below:

$$Total Score=W\sum_{Sj}^{Si} (\text{G}\text{0}\text{.O}\text{c}\text{.S}\text{v}\text{)}$$

The general outcome (G_O_) refers to whether the studies included are in line with what was seen in the clinic: effective drugs are approved, and ineffective drugs have their development terminated or are withdrawn. This shall be only considered for functional outcomes (category II) as defined below. Other findings (e.g. histopathological changes) shall not be considered. Each study’s outcome can be positive (Y), negative (N) or conflicting/partially in line (P). A study shall be classified as positive if it shows a statistically significant difference as reported by the authors in the functional outcome and negative if it did not. Studies which initially show a statistically significant difference and during the study lose such significance shall be classified as partially in line (P) unless such effect is characteristic of the drug/drug class being tested. Studies which report conflicting results in different functional outcomes analysed shall also be classified as (P). The score of each drug is calculated by the majority of studies, and it can be 0, 0.5 or 1. A score of 1 means that most studies are in line with the clinic, indicating the model could reliably predict human efficacy for that specific drug. A model which scores 1 in most drugs has most of the relevant pathways used for the treatment of a condition, meaning it is more likely to be able to predict human response to a drug involving those pathways.

Studies classified as category I (as defined below) are excluded as they, per definition, did not assess the functional outcomes used or comparable to the ones used in the clinical setting. In case a drug only has category I articles or has no published studies on the model being validated, the score shall be 10%, standing for ‘unclear’. For example, if a drug has five studies in the model being validated, of which three were positive, one conflicting or partially positive and one negative, this multiplier’s value is 1. If the number of studies with a positive and a negative assessment is the same or if most studies are conflicting, the section gets a multiplier with half of the points (0.5). If the majority is negative, the section gets a multiplier of 0.

The outcome context multiplier is calculated based on the weighted value of each study according to which category (I or II) a given study was classified. The classification in categories is based on the type of outcomes they report and is defined below:

**Category I:** Genetic/Biochemical/Histopathological (GBH): if an article only reports outcomes related to changes in gene expression, biochemical and/or histopathological marks of disease (e.g. inflammatory cell infiltration or blood levels of a commonly elevated enzyme), it shall be classified as category I. Articles in this category get a 0.5 multiplier for the outcome context score;

**Category II:** Functional: any article that reports at least one functional outcome shall be classified in category II. Functional outcomes are outcomes that can be considered ‘clinically relevant’ outcomes. These outcomes measure the function affected by the disease, offering the highest translational value for the clinical context. These outcomes are defined by the (mostly primary) outcomes used in pivotal trials (usually phases II/III) of approved drugs. If no drug is approved for a given indication, trials in the pipeline can be used as a reference. If there are no clinical trials for that indication, such outcomes can be defined in consultation with experts in the field. The justification for the selected outcomes must be provided (specifically in question 8.1). Measures of muscle strength in degenerative muscle diseases (e.g. grip strength of fore- and hindlimbs in Duchenne Muscular Dystrophy) are examples of functional outcomes. Well-established surrogate outcomes that would otherwise be classified as genetic/biochemical/histopathological outcomes – such as HIV viral load for HIV infection/AIDS, glycaemic parameters (e.g. blood glucose, HbA1c, oral glucose tolerance test (OGTT) and urinary glucose) and cholesterol levels for diabetes and atherosclerosis, respectively – shall also be considered functional outcomes. Articles in this category get a 1.0 multiplier for the outcome context. Thus, if of 3 studies, 2 are category II and one category I, the outcome context multiplier is 2.5 (two full points plus one half-point) divided by 3 (total number of studies), which equals 0.8333.

The last multiplier is the subsection value, which is a simple division of 1 by the number of identified drug classes. An indication that has two drug classes approved (7.1.1 and 7.1.2, for instance), has a section value of 0.5. Within a subsection (i.e. each drug class), the subsection value is divided equally among all drugs. The final score (product of all multipliers) is then multiplied by the section’s weight to achieve the final score. The same approach is taken for ineffective drugs.

**7.3 Have drugs with different mechanisms of action and acting on different pathways been tested in this model? If so, which?**

Describe whether the diversity of known mechanisms of action of drugs tested in humans was also tested in the model, providing a better characterisation of it.

**How to score?**

This question shall be answered by reporting the number of mechanisms of action of approved and failed drugs (in reference to questions 7.1 and 7.2) in comparison to the number of mechanisms of action assessed in the model being validated. The score of this question is calculated based on the proportionality of the mechanisms of action tested in the model. For instance, if there are five known mechanisms of action for which there are approved drugs and 3 for failed drugs, the total number of identified mechanisms of action is 8. If based on the answers in questions 7.1 and 7.2, 5 of these mechanisms have been tested on this model, the score is calculated by multiplying 5/8 by the weight of this question.

**8. Endpoint Validation**

**8.1 Are the endpoints used in preclinical studies the same or translatable to the clinical endpoints?**

Describe whether the endpoints used in at least one study included in the pharmacological validation are the same or can be translated to the clinical endpoints commonly used in clinical trials, indicating their feasibility in the model. Translatable endpoints can be defined as endpoints that measure the same or similar parameters but in a different context, often adapted to be performed in animals, such as muscle strength (e.g. fore- or hindlimbs grip strength on rotarod in comparison with the execution of specific tasks that measure muscle strength in humans). Well-established surrogate endpoints (e.g. blood pressure in hypertension, viral load in HIV infection or blood glucose in diabetes) shall also be considered surrogate endpoints. A discussion on the translatability of such endpoints the justification for their classification in category I or II, as defined in question 7.1, must be provided. In addition to scientific literature, guidelines by regulatory agencies such as the FDA and EMA should be used to substantiate this discussion whenever relevant.

**How to score?**

*Yes.*

All but one study performed in ZDF with agents to treat type 2 diabetes have used glycaemic parameters (e.g. glycaemia, HbA1c, OGTT) or other measures of insulin sensitivity. These measurements also represent the primary outcomes of trials testing new drugs for the treatment of type 2 diabetes, which aim to control glycaemia.

*No.*

Most studies performed in ZDF with agents to treat type 2 diabetes focus on histopathological improvement of the pancreas and gene expression. Glycaemic parameters (e.g. glycaemia, HbA1c, OGTT) or other measures of insulin sensitivity, which are often the primary outcomes of trials testing new drugs for the treatment of type 2 diabetes, are not included.

**8.2 Are the methods used to assess preclinical endpoints comparable to the ones used to assess related clinical endpoints?**

Describe whether the methods used to assess these endpoints (e.g. biochemical techniques) are comparable to the ones used to assess such endpoints in the clinical setting. This shall also include an assessment of the state of mind (e.g. wakefulness and/or willingness) if relevant.

**How to score?**

*Yes.*

The measure of strength for both extension and flexion was taken in awake dogs, based on tasks they had to perform and were previously drilled. This is similar to DMD boys, who have to run a certain distance or perform certain tasks (e.g. get up from the floor without using hands).

*No.*

The measure of strength for both extension and flexion was taken in anesthetised dogs, in which flexion or extension was caused by electrical stimulation of muscles. DMD boys have to run a certain distance or perform certain tasks (e.g. get up the floor without using hands) while being awake and fully aware of the tasks.

# D. Uncertainty Factor

The uncertainty factor is calculated as the percentage of the ratio of subsections answered with “Unclear” by the total number of subsections. This factor helps discriminate models which achieved a low final score because they are not well characterised. The number of subsections which had ‘unclear’ as an answer shall be divided by the total number of subsections.

If of 43 subsections, 15 were answered with ‘unclear’ either due to a lack of data or conflicting evidence, the uncertainty factor would be calculated as follows: 15/43*100 = 34.9%.

# E. Similarity Factor

The similarity factor is calculated as the percentage of the ratio of subsections answered the same for two or more animal models being compared by the total number of subsections. This factor helps contextualise differences or the absence thereof in the scores of different animal models. The number of subsections which have the same answer in both models shall be divided by the total number of subsections. Answers can be ‘Yes’; ‘Yes, completely’; ‘Yes, partially’; ‘No’; or ‘Unclear’.

For the SNH Validation question 2.1, it means having the same symptoms modelled, partially modelled or not modelled. The same applies to question 6.1 (Histological Validation). For the Pharmacological Validation questions 7.1 and 7.2, the same answer refers to the concordance with the clinical setting (General Outcome – G_o_). For instance, if in model A, drug 1 had 20 articles in line with the clinical findings and one not, its G_o_ is 1.0. If in model B, drug 1 had 6 articles in line with the clinical findings and 3 not, its G_o_ is also 1.0. These subsections would then be considered as ‘same answer’ and would be counted for the calculation of the similarity factor. For question 7.3, the number of tested mechanisms of action must be the same.

An example of how to calculate the similarity factor based on the validated models of DMD is presented in Table E1. Calculation of the similarity factor for the mdx mouse and GRMD dog (see S5 Supporting Information – Validation DMD). Subsections from questions 2.1 and 6.1 are presented in the order they appear in the mdx mouse validation. Question/subsections with different answers are presented in bold.

**Table E1.** Example of how to calculate the similarity factor per question. Parameters are presented in the same order they appear in the mdx mouse validation sheet (S5 Supporting Information – Validation DMD).

| **Question (Subsections = 33)** | **Mdx mouse** | **GRMD dog** |
| --- | --- | --- |
| 1.1 | Yes, completely | Yes, completely |
| 1.2 | **Yes, partially** | **Yes, completely** |
| 2.1.1 Reduce grown-up weight | Modelled | Modelled |
| 2.1.2 ECG abnormality | Modelled | Modelled |
| 2.1.3 Cardiomyopathy | Modelled | Modelled |
| 2.1.4 Reduced lifespan | **Partially modelled** | **Modelled** |
| 2.1.5 Muscle wasting | **Partially modelled** | **Modelled** |
| 2.1.6 Cognitive and CNS defects | **Partially modelled** | **Unclear** |
| 2.1.7 Loss of ambulation | **Not modelled** | **Partially modelled** |
| 2.2.1 | **No** | **Yes** |
| 2.2.2 | Yes, partially | Yes, partially |
| 2.2.3 | Yes, completely | Yes, completely |
| 2.2.4 | Yes, partially | Yes, partially |
| 3.1.1 Dystrophin | Yes | Yes |
| 3.1.2 Utrophin | Yes | Yes |
| 3.2 | Yes, partially | Yes, partially |
| 3.3.1 Dystrophin | Yes, completely | Yes, completely |
| 3.3.2 Utrophin | Yes, completely | Yes, completely |
| 4.1 | Yes, completely | Yes, completely |
| 4.2 | Yes, completely | Yes, completely |
| 5.1 | Yes, partially | Yes, partially |
| 6.1.2 Limb muscle fibrosis | **Modelled** | **Partially modelled** |
| 6.1.1 Muscle regeneration | **Not modelled** | **Modelled** |
| 6.1.3 Adipose tissue | **Not modelled** | **Partially modelled** |
| 7.1.1.1 Predinisone | **G_o_ = 1** | **G_o_ = 0.1** |
| 7.1.1.2 Deflazacort | **G_o_ = 1** | **G_o_ = 0.1** |
| 7.1.2 Ataluren | **G_o_ = 1** | **G_o_ = 0.1** |
| 7.1.3 Eterplirsen | **G_o_ = 1** | **G_o_ = 0.1** |
| 7.2.1 Bestatin | G_o_ = 0.1 | G_o_ = 0.1 |
| 7.2.2 Stamulumab | G_o_ = 0.1 | G_o_ = 0.1 |
| 7.3 | **5** | **1** |
| 8.1 | **Yes** | **Yes** |
| 8.2 | **Yes, completely** | **No** |
| Total of # subsections | 16 | |
| Similarity Factor | 16/33 = 48.5% | |

# F. Reporting Quality and Risk of Bias Assessment

A reporting quality and risk of bias assessment of all articles in domain *7. Pharmacological Validation* shall be included. The reporting quality and risk of bias assessment shall be based on an adaptation of the criteria defined by the ARRIVE guidelines and on SYRCLE’s Risk of Bias Assessment Tool [1,2]. A summary of the reporting quality and risk of bias assessments shall be provided per drug in the respective drug section (see S5 Supporting Information). When calculating the summary with all pharmacological studies, it is important to remove studies with more than one drug that has been administered to the same model to avoid duplication (see S7 Supporting Information).

While it is not our goal to focus on internal validity, we included the assessment of reporting quality and risk of bias to draw attention to the often poor internal validity of animal studies. It is up to the researchers to evaluate these assessments and their impact on the results of the studies of interest.

**F1. Reporting Quality**

It is important to note that the description of such parameters must be clear. For instance, ‘standard’ lab chow, housing or ‘controlled’ temperature and humidity are not considered as proper descriptions as well as ‘carrying the experiments according to the regulations of a given institution’. Answers can be a ‘Yes (Y)’ if the paper properly described the parameter or ‘No (N)’ if it did not. The criteria for reporting quality are housing, husbandry, sample size, sample size calculation, acclimatisation, sex, background strain, blinding and randomisation.

**Housing**

1) type of facility: specify whether it is a regular facility or not (e.g. specific pathogen-free [SPF]);

2) type of cage or housing: describe the cage or housing (tank for fish) shape (if relevant) and material etc.;

3) bedding material: describe the material used in the bedding, if applicable;

4) number of cage companions: describe whether animals were individually housed or in groups and, if the latter, how big the groups were;

**Husbandry**

5) breeding programme: describe whether the animals were bred in-house or were bought from another company;

6) light/dark cycle: describe how many hours of light and dark the animals were exposed to;

7) temperature and humidity: describe both what the temperature and humidity were in the room were animals were housed;

8) quality of water etc (for fish): describe the quality parameters of the water;

9) type of food: describe what kind of food was provided, including the provider if bought commercially. If prepared in-house, describe the composition of the food;

10) access to food and water: describe whether animals could eat and drink *ad libitum* and if not, what were the periods animals could access food and water, and how much they could eat or drink;

11) environmental enrichment: describe whether there was any environmental enrichment (such as treadmills). If no environmental enrichment was provided, whether this was clearly reported;

**Sample size**

12) specify the total number of animals used in each experiment, and the number of animals in each experimental group;

**Sample size calculation**

13) explain how the number of animals was arrived at. Provide details of any sample size calculation used;

**Any Blinding**

14) describe whether blinding at any level and detail was described in the methodology;

**Any Randomisation**

15) describe whether randomisation at any level and detail was described in the methodology;

**Acclimatisation**

16) describe how long animals had to adapt to their facilities and/or training for functional measures;

**Sex**

17) describe whether animal sex was disclosed and if so, whether it included male (M), female (F) or male and female animals (B) in the treatment groups;

**Background strain**

18) whenever relevant, describe whether the background strain for the control was disclosed and which strain it was;

19) whenever relevant, describe whether the background strain for the model was disclosed and which strain it was;

**F2. Risk of Bias (Signalling Question)**

We suggest users to read and use of the Risk of Bias tool published by SYRCLE, which provides more information on its development and usability [2]. Ideally, when assessing the risk of bias, users should take notes on the justification of their assessment. However, often the volume of studies will prevent the reporting of individual and summarised risks of bias at that level of detail. Thus, answers to each parameter follow the main signalling questions in which ‘Yes (Y)’ describes studies with a low risk of bias; ‘Unclear (U)’ studies in which there is not enough information to assess the risk of bias; and ‘No (N)’ studies with a high risk of bias. It is important to note that the additional signalling questions described in the original paper may help users conduct a proper risk of bias evaluation. The parameter referring to other risks of bias shall be reported with a brief explanation in the comments. The criteria for risk of bias are blinding, randomisation, sequence generation, baseline characteristics, incomplete outcome data, selective outcome reporting and ‘other’. All criteria are defined below:

**Blinding**

20) allocation concealment (Was the allocation adequately concealed?): describe the method used to conceal the allocation sequence in sufficient detail to determine whether intervention allocations could have been foreseen before or during enrolment;

21) blinded outcome assessment (Was the outcome assessor blinded?): describe all measures used, if any, to blind outcome assessors from knowing which intervention each animal received. Provide any information relating to whether the intended blinding was effective;

22) blinded operations (Were the caregivers and/or investigators blinded from knowledge which intervention each animal received during the experiment?): Describe all measures used, if any, to blind trial caregivers and researchers from knowing which intervention each animal received. Provide any information relating to whether the intended blinding was effective.

**Randomisation**

23) random cage allocation (Were the animals randomly housed during the experiment?): if animals are not housed individually, whether the assignment of a cage to a treatment was randomised and how this was achieved;

24) random outcome assessment (Were animals selected at random for outcome assessment?): Describe whether animals were selected at random for outcome assessment, and which methods to select the animals, if any, were used;

**Sequence Generation (Was the allocation sequence adequately generated and**

**applied?)**

25) describe the methods used, if any, to generate the allocation sequence in sufficient detail to allow an assessment whether it should produce comparable groups;

**Baseline Characteristics (Were the groups similar at baseline or were they adjusted for confounders in the analysis?)**

26) describe all the possible prognostic factors or animal characteristics, if any, that are compared in order to judge whether or not intervention and control groups were similar at the start of the experiment;

**Incomplete Outcome Data (Were incomplete outcome data adequately addressed?)**

27) Describe the completeness of outcome data for each main outcome, including attrition and exclusions from the analysis. State whether attrition and exclusions were reported, the numbers in each intervention group (compared with total randomised animals), reasons for attrition or exclusions, and any re-inclusions in analyses for the review;

**Selective Outcome Reporting (Are reports of the study free of selective outcome reporting?)**

28) State how selective outcome reporting was examined and what was found;

**Other (Was the study apparently free of other problems that could result in high risk of bias?)**

29) State any important concerns about bias not covered by other domains in the tool.

References

1. Kilkenny C, Browne WJ, Cuthill IC, Emerson M, Altman DG. Improving Bioscience Research Reporting: The ARRIVE Guidelines for Reporting Animal Research. PLOS Biol [Internet]. 2010 Jun 29;8(6):e1000412. Available from: https://doi.org/10.1371/journal.pbio.1000412

2. Hooijmans CR, Rovers MM, Vries RBM De, Leenaars M, Ritskes-hoitinga M, Langendam MW. SYRCLE’s risk of bias tool for animal studies. BMC Med Res Methodol [Internet]. 2014;14(1):1–9. Available from: BMC Medical Research Methodology

1. <https://meshb.nlm.nih.gov/search> [↑](#footnote-ref-2)
2. <https://www.embase.com/#emtreeSearch/default> [↑](#footnote-ref-3)
