# Supplementary material for: A standardised framework to identify optimal animal models for efficacy assessment in drug development
Source: PLoS One. 2019 Jun 13;14(6):e0218014. doi: 10.1371/journal.pone.0218014 (PMC6563989; doi:10.1371/journal.pone.0218014)
Supplement: S3 Supporting Information — (DOCX) [file pone.0218014.s003.docx]

# S3 Supporting Information – FIMD Template

| **MODEL NAME** |  |
| --- | --- |
| **INDICATION** |  |
| **VALIDATION DATE** |  |
| **TOTAL SUBSECTIONS** |  |
| **TOTAL SCORE** |  |
| **VALIDATION LEVEL (%)** |  |
| **UNCERTAINTY FACTOR (%)** |  |
| **HISTORICAL BACKGROUND** | |
|  | |

| **1. EPIDEMIOLOGICAL VALIDATION** | |
| --- | --- |
| 1.1 Is the model able to simulate the disease in the relevant sexes? | Score |
|  |  |
| 1.2 Is the model able to simulate the disease in the relevant age groups (juvenile, adult or ageing)? | Score |
|  |  |

| **2. SYMPTOMATOLOGY AND NATURAL HISTORY (SNH) VALIDATION** | |
| --- | --- |
| 2.1 Is the model able to replicate the symptoms commonly present in this disease? If so, which ones? | Score |
|  |  |
| 2.1.1 Symptoms modelled |  |
| Symptom 1 |  |
| Symptom 2 |  |
| 2.1.2 Symptoms partially modelled |  |
| Symptom 1 |  |
| Symptom 2 |  |
| 2.1.3 Symptoms not modelled |  |
| Symptom 1 |  |
| Symptom 2 |  |
| 2.2 Is the natural history of the disease similar to humans regarding: |  |
| 2.2.1 Time to onset; | Score |
|  |  |
| 2.2.2 Disease progression; | Score |
|  |  |
| 2.2.3 Duration of symptoms; | Score |
|  |  |
| 2.2.4 Severity. | Score |
|  |  |

| **3. GENETIC VALIDATION** | |
| --- | --- |
| 3.1 Does this species also have orthologous genes and/or proteins involved in the human disease? If so, which? | Score |
|  |  |
| 3.1.1 Gene 1 |  |
|  |  |
| 3.1.2 Gene 2 |  |
|  |  |
| 3.2 If so, are the relevant genetic mutations or alterations also present in the orthologous genes/proteins? | Score |
|  |  |
| 3.2.1 Gene 1 |  |
|  |  |
| 3.2.2 Gene 2 |  |
|  |  |
| 3.3 If so, is the expression of such orthologous genes and/or proteins similar to the human condition? | Score |
|  |  |
| 3.3.1 Gene 1 |  |
|  |  |
| 3.3.2 Gene 2 |  |
|  |  |

| **4. BIOCHEMICAL VALIDATION** | |
| --- | --- |
| 4.1 If there are known pharmacodynamic (PD) biomarkers related to the pathophysiology of the disease, are they also present in the model? | Score |
|  |  |
| 4.1.1 Biomarker 1 |  |
|  |  |
| 4.1.2 Biomarker 2 |  |
|  |  |
| 4.2 Do these PD biomarkers behave similarly to humans’? | Score |
|  |  |
| 4.2.1 Biomarker 1 |  |
|  |  |
| 4.2.2 Biomarker 2 |  |
|  |  |
| 4.3 If there are known prognostic biomarkers related to the pathophysiology of the disease, are they also present in the model? | Score |
|  |  |
| 4.3.1 Biomarker 1 |  |
|  |  |
| 4.3.2 Biomarker 2 |  |
|  |  |
| 4.4 Do these prognostic biomarkers behave similarly to humans’? | Score |
|  |  |
| 4.4.1 Biomarker 1 |  |
|  |  |
| 4.4.2 Biomarker 2 |  |
|  |  |

| **5. AETIOLOGICAL VALIDATION** | |
| --- | --- |
| 5.1 Is the aetiology of the disease similar to humans’? | Score |
|  |  |
|  |  |

| **6. HISTOLOGICAL VALIDATION** | |
| --- | --- |
| 6.1 Do the histopathological structures in relevant tissues resemble the ones found in humans? | Score |
|  |  |
| 6.1.1 Histopathological features modelled |  |
| Histopathological feature 1 |  |
| Histopathological feature 2 |  |
| 6.1.2 Histopathological features partially modelled |  |
| Histopathological feature 1 |  |
| Histopathological feature 2 |  |
| 6.1.3 Histopathological features not modelled |  |
| Histopathological feature 1 |  |
| Histopathological feature 2 |  |

| **7. PHARMACOLOGICAL VALIDATION** | |
| --- | --- |
| 7.1 Are effective drugs in humans also effective in this model? | Score |
|  |  |
| 7.1.1 Drug Class 1 |  |
| 7.1.1.1 Drug 1 |  |
| Methodology:  Results: |  |

| Reporting Quality and Risk of Bias Assessment | | | | | |
| --- | --- | --- | --- | --- | --- |
| Parameter (N = X) | Y (%) |  | Y (%) | Risk of Bias (N = X) | Y/U (%) |
| Type of Facility | 0.0 | Environmental Enrichment | 0.0 | Allocation Concealment | 0/0 |
| Type of Cage or Housing | 0.0 | Any Blinding | 0.0 | Blinded Outcome Assessment | 0/0 |
| Bedding Material | 0.0 | Any Randomisation | 0.0 | Blinded Operations | 0/0 |
| N Cage Companions | 0.0 | Sample Size | 0.0 | Random Cage Allocation | 0/0 |
| Breeding Programme | 0.0 | Sample Size Calculation | 0.0 | Random Outcome Assessment | 0/0 |
| Light/Dark Cycle | 0.0 | Acclimatisation | 0.0 | Sequence Generation | 0/0 |
| Temperature and Humidity | 0.0 | Sex Disclosed | 0.0 | Baseline Characteristics | 0/0 |
| Quality of the Water (fish) | - | Male/Female/Both  (N = X) | 0/0/0 | Incomplete Outcome Data | 0/0 |
| Type of Food | 0.0 | Background Control | - | Selective Outcome Reporting | 0/0 |
| Access to Food and Water | 0.0 | Background Model | - | Other | 0/0 |

|  |  |
| --- | --- |
| 7.1.1.2 Drug 2 |  |
| Methodology:  Results: |  |

| Reporting Quality and Risk of Bias Assessment | | | | | |
| --- | --- | --- | --- | --- | --- |
| Parameter (N = X) | Y (%) |  | Y (%) | Risk of Bias (N = X) | Y/U (%) |
| Type of Facility | 0.0 | Environmental Enrichment | 0.0 | Allocation Concealment | 0/0 |
| Type of Cage or Housing | 0.0 | Any Blinding | 0.0 | Blinded Outcome Assessment | 0/0 |
| Bedding Material | 0.0 | Any Randomisation | 0.0 | Blinded Operations | 0/0 |
| N Cage Companions | 0.0 | Sample Size | 0.0 | Random Cage Allocation | 0/0 |
| Breeding Programme | 0.0 | Sample Size Calculation | 0.0 | Random Outcome Assessment | 0/0 |
| Light/Dark Cycle | 0.0 | Acclimatisation | 0.0 | Sequence Generation | 0/0 |
| Temperature and Humidity | 0.0 | Sex Disclosed | 0.0 | Baseline Characteristics | 0/0 |
| Quality of the Water (fish) | - | Male/Female/Both  (N = X) | 0/0/0 | Incomplete Outcome Data | 0/0 |
| Type of Food | 0.0 | Background Control | - | Selective Outcome Reporting | 0/0 |
| Access to Food and Water | 0.0 | Background Model | - | Other | 0/0 |

| 7.1.2 Drug Class 2 |  |
| --- | --- |
| 7.1.2.1 Drug 1 |  |
| Methodology:  Results: |  |

| Reporting Quality and Risk of Bias Assessment | | | | | |
| --- | --- | --- | --- | --- | --- |
| Parameter (N = X) | Y (%) |  | Y (%) | Risk of Bias (N = X) | Y/U (%) |
| Type of Facility | 0.0 | Environmental Enrichment | 0.0 | Allocation Concealment | 0/0 |
| Type of Cage or Housing | 0.0 | Any Blinding | 0.0 | Blinded Outcome Assessment | 0/0 |
| Bedding Material | 0.0 | Any Randomisation | 0.0 | Blinded Operations | 0/0 |
| N Cage Companions | 0.0 | Sample Size | 0.0 | Random Cage Allocation | 0/0 |
| Breeding Programme | 0.0 | Sample Size Calculation | 0.0 | Random Outcome Assessment | 0/0 |
| Light/Dark Cycle | 0.0 | Acclimatisation | 0.0 | Sequence Generation | 0/0 |
| Temperature and Humidity | 0.0 | Sex Disclosed | 0.0 | Baseline Characteristics | 0/0 |
| Quality of the Water (fish) | - | Male/Female/Both  (N = X) | 0/0/0 | Incomplete Outcome Data | 0/0 |
| Type of Food | 0.0 | Background Control | - | Selective Outcome Reporting | 0/0 |
| Access to Food and Water | 0.0 | Background Model | - | Other | 0/0 |

| 7.2 Are ineffective drugs in humans also ineffective in this model? | Score |
| --- | --- |
|  |  |
| 7.2.1 Drug Class 1 |  |
| 7.2.1.1 Drug 1 |  |
| Methodology:  Results: |  |

| Reporting Quality and Risk of Bias Assessment | | | | | |
| --- | --- | --- | --- | --- | --- |
| Parameter (N = X) | Y (%) |  | Y (%) | Risk of Bias (N = X) | Y/U (%) |
| Type of Facility | 0.0 | Environmental Enrichment | 0.0 | Allocation Concealment | 0/0 |
| Type of Cage or Housing | 0.0 | Any Blinding | 0.0 | Blinded Outcome Assessment | 0/0 |
| Bedding Material | 0.0 | Any Randomisation | 0.0 | Blinded Operations | 0/0 |
| N Cage Companions | 0.0 | Sample Size | 0.0 | Random Cage Allocation | 0/0 |
| Breeding Programme | 0.0 | Sample Size Calculation | 0.0 | Random Outcome Assessment | 0/0 |
| Light/Dark Cycle | 0.0 | Acclimatisation | 0.0 | Sequence Generation | 0/0 |
| Temperature and Humidity | 0.0 | Sex Disclosed | 0.0 | Baseline Characteristics | 0/0 |
| Quality of the Water (fish) | - | Male/Female/Both  (N = X) | 0/0/0 | Incomplete Outcome Data | 0/0 |
| Type of Food | 0.0 | Background Control | - | Selective Outcome Reporting | 0/0 |
| Access to Food and Water | 0.0 | Background Model | - | Other | 0/0 |

| 7.2.1.2 Drug 2 |  |
| --- | --- |
| Methodology:  Results: |  |

| Reporting Quality and Risk of Bias Assessment | | | | | |
| --- | --- | --- | --- | --- | --- |
| Parameter (N = X) | Y (%) |  | Y (%) | Risk of Bias (N = X) | Y/U (%) |
| Type of Facility | 0.0 | Environmental Enrichment | 0.0 | Allocation Concealment | 0/0 |
| Type of Cage or Housing | 0.0 | Any Blinding | 0.0 | Blinded Outcome Assessment | 0/0 |
| Bedding Material | 0.0 | Any Randomisation | 0.0 | Blinded Operations | 0/0 |
| N Cage Companions | 0.0 | Sample Size | 0.0 | Random Cage Allocation | 0/0 |
| Breeding Programme | 0.0 | Sample Size Calculation | 0.0 | Random Outcome Assessment | 0/0 |
| Light/Dark Cycle | 0.0 | Acclimatisation | 0.0 | Sequence Generation | 0/0 |
| Temperature and Humidity | 0.0 | Sex Disclosed | 0.0 | Baseline Characteristics | 0/0 |
| Quality of the Water (fish) | - | Male/Female/Both  (N = X) | 0/0/0 | Incomplete Outcome Data | 0/0 |
| Type of Food | 0.0 | Background Control | - | Selective Outcome Reporting | 0/0 |
| Access to Food and Water | 0.0 | Background Model | - | Other | 0/0 |

| 7.2.2 Drug Class 2 |  |
| --- | --- |
| 7.2.2.1 Drug 1 |  |
| Methodology:  Results: |  |

| Reporting Quality and Risk of Bias Assessment | | | | | |
| --- | --- | --- | --- | --- | --- |
| Parameter (N = X) | Y (%) |  | Y (%) | Risk of Bias (N = X) | Y/U (%) |
| Type of Facility | 0.0 | Environmental Enrichment | 0.0 | Allocation Concealment | 0/0 |
| Type of Cage or Housing | 0.0 | Any Blinding | 0.0 | Blinded Outcome Assessment | 0/0 |
| Bedding Material | 0.0 | Any Randomisation | 0.0 | Blinded Operations | 0/0 |
| N Cage Companions | 0.0 | Sample Size | 0.0 | Random Cage Allocation | 0/0 |
| Breeding Programme | 0.0 | Sample Size Calculation | 0.0 | Random Outcome Assessment | 0/0 |
| Light/Dark Cycle | 0.0 | Acclimatisation | 0.0 | Sequence Generation | 0/0 |
| Temperature and Humidity | 0.0 | Sex Disclosed | 0.0 | Baseline Characteristics | 0/0 |
| Quality of the Water (fish) | - | Male/Female/Both  (N = X) | 0/0/0 | Incomplete Outcome Data | 0/0 |
| Type of Food | 0.0 | Background Control | - | Selective Outcome Reporting | 0/0 |
| Access to Food and Water | 0.0 | Background Model | - | Other | 0/0 |

| 7.3 Have drugs with different mechanisms of action and acting on different pathways been tested in this model? If so, which? | Score |
| --- | --- |
|  |  |

| **8. ENDPOINT VALIDATION** | |
| --- | --- |
| 8.1 Are the endpoints used in preclinical studies the same or translatable to the clinical endpoints? | Score |
|  |  |
| 8.2 Are the methods used to assess preclinical endpoints comparable to the ones used to assess related clinical endpoints? | Score |
|  |  |
