# Supplementary material for: A standardised framework to identify optimal animal models for efficacy assessment in drug development
Source: PLoS One. 2019 Jun 13;14(6):e0218014. doi: 10.1371/journal.pone.0218014 (PMC6563989; doi:10.1371/journal.pone.0218014)
Supplement: S4 Supporting Information — (DOCX) [file pone.0218014.s004.docx]

# S4 Supporting Information - Pilot Study – Type 2 Diabetes (T2D)

T2D was chosen as a disease which has complex pathophysiology and for which an extensive set of therapies is available [1]. We used a simplified version of FIMD to test its applicability in a pilot study aiming at identifying potential shortcomings of the framework before getting input from the expert panel. Therefore, there are some differences in the method used in the pilot study to the one described in S2 Supporting Information. Given the large number of studies in T2D, in question 7.1 (Pharmacological Validation), only one approved drug from each class was included. The preference was for first-in-class drugs which are still in the market (either USA or EU). If the first-in-class had been withdrawn, the second-in-class was then preferred (and so on, if necessary). Including every first-in-class drug allowed an easy and clear standardisation as there can be only one first-in-class that does not change over time (unlike best- or last-in-class, which may be subjective and/or change over time). Additionally, it is likely that older drugs would have more studies published. Moreover, the search was conducted only on PubMed. These simplifications allowed us to conduct the pilot study in a shorter period while still testing the main features of FIMD.

The Zucker Diabetic Fatty (ZDF) rat and the db/db mouse were both chosen for being routinely used in drug screening for antidiabetic drugs. A total of 195 publications was included for the ZDF rat and 282 for the db/db mouse. The relative scores per parameter are presented in Fig A.

**Fig A. T2D models results.** Radar plot with the scores per parameter per model of T2D. The closer a parameter is to the edge, the better the model simulates that aspect of the human disease. SNH – Symptomatology and Natural History.

Compared to the DMD models, there are only minor differences since the similarity factor between the two models is almost 90%. Both T2D models have uncertainty factors of over 20% mostly due to the lack of studies in the pharmacological validation.

A reporting quality assessment was included based on parameters adapted from the ARRIVE guidelines (see S2 Supporting Information, section F). Publications with experiments on two models were counted separately for each model while studies with more than one arm of an included drug were counted only once. We used a shortened list of reporting quality parameters according to the ARRIVE guidelines: instead of specifically assessing whether the animals were randomised into the treatment groups, the randomisation method was mentioned, and cage randomisation was performed, we assessed whether the authors provided any information on randomisation at all. The same was applied to blinding. For other multifaceted parameters (e.g. housing, husbandry), any facets mentioned warranted a positive evaluation. Table A shows the aggregated data from all drugs and Table B provides more information on the use of different sexes in the studies that disclosed this information.

**Table A**: percentage of studies which comply totally or partially with reporting quality parameters adapted from the ARRIVE guidelines per model.

| Parameter | ZDF rat  (n = 96) | Db/db mouse  (n = 169) | Total  (n = 251) |
| --- | --- | --- | --- |
|  | (%) | | |
| Housing | 36.3 | 34.2 | 35.1 |
| Husbandry | 84.4 | 78.3 | 80.9 |
| Sample Size | 91.2 | 93.2 | 92.8 |
| Sample Size Calculation | 0.0 | 0.0 | 0.0 |
| Blinding | 0.0 | 1.2 | 0.8 |
| Randomisation | 35.2 | 34.2 | 33.7 |
| Acclimatisation | 48.4 | 33.5 | 39.0 |
| Sex | 91.2 | 88.8 | 90.0 |
| Background Strain | - | 78.3 | 87.2 |

**Table B:** percentage of studies which included only male, female or both sexes per model.

| Sex | ZDF rat  (n = 83) | Db/db mouse  (n = 143) | Total  (n = 226) |
| --- | --- | --- | --- |
|  | (%) | | |
| Male | 91.6 | 84.6 | 87.2 |
| Female | 2.4 | 12.6 | 8.8 |
| Both | 6.0 | 2.8 | 4.0 |

Most studies did not provide any information on the randomisation (66.3%) and blinding (99.2%). No publication explained how they arrived at the number of animals for each experiment. Also, male animals are almost ten times more likely to be used than females while the use of both sexes is sporadic, making up only 4% of all studies. There percentages of compliance of studies with the ZDF rat and the db/db mouse are comparable. The findings from the quality reporting assessment are in line with the literature and with results from the full validation [2–6].

| **MODEL NAME** | Zucker Diabetic Fatty (ZDF)-*Lepr^fa^*/Crl rat |
| --- | --- |
| **INDICATION** | Type 2 Diabetes Mellitus (T2D) |
| **VALIDATION DATE** | 18.4.2017 |
| **TOTAL SUBSECTIONS** | 59 |
| **TOTAL SCORE** | 68.36 |
| **UNCERTAINTY FACTOR (%)** | 20.3 |
| **HISTORICAL BACKGROUND** | |
| The Zucker Fatty (ZF) rat was identified after crossing Merck M-strain with Sherman rats in 1961 [7,8]. In these mice, the leptin receptor is mutated (the *fatty* or *fa* mutation). Another mutation – an autosomal recessive defect in pancreatic β-cells transcription inherited independently from the leptin mutation – occurred in a colony of outbred ZF rats, leading to the characterisation of a substrain with a diabetogenic phenotype called Zucker Diabetic Fatty (ZDF) rat [9]. Further establishment of the line has been done by inbreeding rats with diabetic lineage [9]. | |

| **1. EPIDEMIOLOGICAL VALIDATION** | |
| --- | --- |
| 1.1 Is the model able to simulate the disease in the relevant sexes? | Score |
| Yes, completely.  Remarks: There are reports of difficulties in being able to properly simulate diabetes in female ZDF rats, which are commonly used as controls to males in preclinical studies [10,11]. However, both Charles River Laboratory (the main supplier of this strain) and Corsetti et al have successfully induced the diabetic state in females by using specific diets (DI2468 and 48% high-fat diet, respectively) [9,11,12]. Furthermore, this specific female resistance to the development of diabetes is in line with what is seen in humans, due to a possible protective effect of oestrogen on pancreatic beta-cells [13,14]. | 2 |
| 1.2 Is the model able to simulate the disease in the relevant age groups (juvenile, adult or ageing)? | Score |
| Yes, partially.  Remarks: Although the time to onset of diabetes has been decreasing in the past years, ZDF rat still develops diabetes somewhat earlier than humans [15]. However, it progresses into adulthood and ageing phases, similarly to the human disease [12,16–20]. | 1 |

| **2. SYMPTOMATOLOGY AND NATURAL HISTORY (SNH) VALIDATION** | |
| --- | --- |
| 2.1 Is the model able to replicate the symptoms commonly present in this disease? If so, which ones? | Score |
| Yes, completely.  The most common symptoms of diabetes type 2 are polyphagia, polydipsia, polyuria, weight loss, fatigue, healing impairment and obesity [21]. The latter may not be considered a symptom per se, being better classified as a co-morbidity. However, due to the high prevalence of this condition in T2D patients and likely association to the pathophysiology, it was also included in this section.  Most of these symptoms are directly connected to the hyperglycaemia and dyslipidaemia and therefore they were translated into the following parameters: hyperglycaemia, hyperinsulinemia followed by hypoinsulinemia, dyslipidaemia (divided into hypercholesterolemia and hypertriglyceridemia according to the common human pathological profile) and obesity. | 13 |

| 2.1.1 Symptoms modelled |  |
| --- | --- |
| Hyperglycaemia: At 5-7 weeks, fasting blood glucose is already higher than lean littermates and with a further increase at 10-12 weeks and stabilising at a few-fold more than lean rats [17,20,22–29]. |  |
| Hyperinsulinemia followed by hypoinsulinemia: insulin levels in ZDF rats are initially significantly higher than their lean littermates, however, they decrease steadily with age due to β-cell insufficiency [12,17,23,27]. |  |
| 2.1.1 Symptoms modelled |  |
| Hypercholesterolemia: high cholesterol levels are found in ZDF rats as well as humans, which eventually leads to atherosclerosis [23,27,28]. |  |
| Hypertriglyceridemia: triglyceride and free fatty acids (FFA) levels in ZDF rats are significantly higher than in lean littermates [17,20,23,25,27,28]. |  |
| Obesity: ZDF rats are significantly heavier than their lean littermates at a young age, but tend to significantly lose weight after overt diabetes, which is consistent with the sudden weight loss seen in humans [17,22,24–27,29,30]. |  |
| 2.2 Is the natural history of the disease similar to humans regarding: |  |
| 2.2.1 Time to onset; | Score |
| No.  Remarks: type 2 diabetes is usually diagnosed around 40 years, although age at diagnosis has been decreasing [15]. The onset of diabetes in male ZDF rats may vary from 6 to 12 weeks, but commonly happening between 7 and 8 weeks [12,16–20].This happens at quite an early stage of life when compared to humans: rats 24 weeks of age are in a similar stage of development as humans around 18 years [31]. | 0 |
| 2.2.2 Disease progression; | Score |
| Yes, partially.  Remarks: Diabetes progression in ZDF rats follows much of the human disease. The increase in sugar/fat intake in diets leads to an increase in insulin secretion, reducing the ability to activate the TK receptor (between 3 and 8 weeks of age). Consequently, this then leads to an increase in glycaemia and β-cell degeneration, finally leading to β-cell apoptosis and glucose intolerance (between 6 and 12 weeks of age) [12,16–19]. The whole process, however, happens at quite an early stage of life when compared to humans: rats 6 months of age are in a similar stage of development as humans around 18 years [31]. Although as mentioned before, the age of onset of diabetes has been decreasing in the past years [15].  As for common diabetic complications, such as nephropathy, retinopathy and neuropathy, ZDF rats can partially reproduce them. They are able to develop renal lesions (such as albuminuria, glomerulosclerosis, tubulointerstitial scarring and inflammation) progressively with age, but these are confounded by natural ageing lesions and hydronephrosis [32–34]. ZDF rats can also develop ocular lesions typical of diabetes such as cataract formation and apoptotic death of lens cells but lack other lesions commonly present in humans, such as pericyte degeneration, microaneurysms, and acellular capillaries [35–37]. Likewise, ZDF rats can partially develop neurological complications, such as decreased caudal motor nerve conduction velocity (MNCV), impairment of acetylcholine-mediated vascular extension of epineural arterioles of the sciatic nerve, but do not show sympathetic neuronal dystrophy [30,38,39]. | 1 |
| 2.2.3 Duration of symptoms; | Score |
| Yes, completely.  Remarks: like humans, once insulin resistance and glucose intolerance are set, β-cell apoptosis follows, worsening the diabetic condition progressively and leading to overt diabetes and diabetic complications [22,40]. | 2 |

| 2.2.4 Severity. | Score |
| --- | --- |
| Yes, partially.  Remarks: the biochemical parameters in ZDF rats are similar to humans in severity, being considerably increased (please see section 2.1). However, the complications that follow after the onset of overt diabetes are somewhat milder in ZDF rats (lack of pericyte degeneration in the retinopathy or sympathetic neuronal dystrophy in nephropathy). | 1 |

| **3. GENETIC VALIDATION** | |
| --- | --- |
| 3.1 Does this species also have orthologous genes and/or proteins involved in the human disease? If so, which? | Score |
| Yes, completely.  Although is widely accepted that diabetes type 2 has genetic factors involved, reliable tracking and identification of such genes remain a challenge [41–48]. This is often referred to as the ‘missing heritability’ [42]. In some cases, T2D can be caused by a single gene (such as mutations on HNF4A for Maturity-Onset-Diabetes of the Young type 1 – MODY1 – or PTF1A for neonatal diabetes) [41,43]. Nonetheless, in most cases it is a polygenic and heterogeneous disease, meaning multiple genes might be involved and different combinations of polymorphisms can lead to the pathological phenotype, which makes the genetic validation of animal models at best extremely challenging [41,43,45–48].  With the advent of Genome-Wide Association Studies (GWAS), many genes have been associated with an increased risk of developing type 2 diabetes, shedding light on the complex genetic architecture of the disease [41,49,50]. However, three of them have been consistently established in GWAS as likely genetic factors: TCF7L2, KCNJ11 and PPARG [41,44,51]. Therefore, these were the only ones included in this section.  All three genes are present in ZDF rats [52–54]. | 8 |
| 3.1.1 TCF7L2 |  |
| TCF7L2 is a gene (also known as also known as TCF-4 or β-catenin interacting protein) located at 10q25.2-q25.3 in humans and at 1q55 in rats that codes a transcription factor involved in the WNT signalling pathway [52,55–58]. This is the strongest and most well-replicated genetic factor associated to T2D [41,44,49,50,59,60]. It acts as a nuclear receptor for β-catenin, which is involved in the secretion of GLP-1 in gut endocrine cells and various other genes [56,57,61]. There are also reports that indicate a possible role in the incretin axis, adipocyte function and glucose production by the liver, despite some of the results being conflicting [58,62–66]. |  |
| 3.1.2 KCNJ11 |  |
| KCNJ11 is a gene (also known as KIR6.2) located at 11p15.1 in humans and at 1q22 in rats that has been associated with an increased risk of developing T2D [49–51,54,67]. It codes for a major subunit of the ATP-sensitive K^+^ channel, an inward-rectifier potassium ion channel present in the pancreatic islets with direct influence over insulin secretion [68,69]. Mutations in this gene are connected to neonatal diabetes [41]. |  |
| 3.1.3 PPARG |  |
| PPARG is a gene that codes for the transcription factor peroxisome proliferator-activated receptor-γ (PPARγ) located at 3p25.2 and at 4q42 in rats in humans [53,70]. The PPARG is involved in adipocyte regulation, fat accumulation and glucose metabolism and it is stimulated by insulin in a ligand-dependent manner [71–74]. Two isoforms exist in humans: PPARγ1 and PPARγ2, the first being expressed in most tissues and the second one mostly in the liver and adipose tissue [75]. The isoform 2 is also the target of thiazolidinediones, such as rosiglitazone. |  |

| 3.2 If so, are the relevant genetic mutations or alterations also present in the orthologous genes/proteins? |  |
| --- | --- |
| Unclear. | 0.5 |
| 3.2.1 TCF7L2 |  |
| Unclear.  In humans, the rs12255372 and rs7903146 alleles are the most strongly associated variants with carriers of such type having reduced insulin secretion but not increased insulin resistance [76]. Being homozygous for the high-risk allele doubles the chance of developing T2D [41]. No studies investigating specific genetic alterations in this gene could be found in PubMed in ZDF rats using the string ("ZDF rat" OR "ZDF rats" OR "Zucker Diabetic Fatty rat" OR "Zucker Diabetic Fatty rats") AND TCF7L2 on 13/04/2017. |  |
| 3.2.2 KCNJ11 |  |
| Unclear.  In humans, the commonest polymorphism is a glutamate to lysine substitution at position 23 (E23K or rs5219) [77–80]. This mutation is linked to a reduction in channel sensitivity, increasing the signal threshold for the release of insulin and impairing serum insulin response [68,77,78,81]. Homozygous carriers of this mutation (KK) have an almost double risk of developing T2D when compared to non-carriers [79].  No studies investigating specific genetic alterations in this gene could be found in PubMed in ZDF rats using the string ("ZDF rat" OR "ZDF rats" OR "Zucker Diabetic Fatty rat" OR "Zucker Diabetic Fatty rats") AND (KCNJ11 OR KIR6.2 OR “KIR 6.2”) on 13/04/2017. |  |
| 3.2.3 PPARG |  |
| Unclear.  In humans, the SNP rs1801282 consists of a proline substitution for alanine in position 12 (Pro12Ala or P12A) of PPARγ2 (the other isoform is not affected), the proline allele is associated with an increased risk of developing T2D, higher BMI and decreased insulin sensitivity while the alanine one confers resistance [49–51,79,80,82–84]. Even though the mechanism is still unclear, the Ala variant has a lower transactivation  Efficiency, reducing stimulation of PPARG target genes and therefore also reducing the levels of adipose tissue mass accumulation [82]. Nonetheless, there are also reports of increased risk of cardiovascular disease in carriers of this polymorphism [85,86].  No studies investigating specific genetic alterations in this gene could be found in PubMed in ZDF rats using the string (PPARgamma) AND ("ZDF rat" OR "ZDF rats" OR "zucker diabetic fatty rat" OR "zucker diabetic fatty rats") on 18/04/2017. |  |
| 3.3 If so, is the expression of such orthologous genes and/or proteins similar to the human condition? | Score |
| Yes, partially. | 1.07 |
| 3.3.1 TCF7L2 |  |
| Unclear.  Two studies found the TCF7L2 gene is overexpressed in carriers of the risk genotype in pancreatic islets of T2D patients, but no changes in TCF7L2 expression were found by study of the Diabetes Genome Anatomy Project (DGAP) [41,62,87]. One study by Shu and colleagues showed a possible protective effect on the islets against glucose- and cytokine-induced apoptosis and function impairment. This study was later retracted due to image duplication and concerns over data reliability [88]. One study in ZDF rats has shown overexpression of TCF7L2 in the islets, unaffected by the blockade of SREBP-1c [89]. |  |
| 3.3.2 KCNJ11 |  |
| Yes, completely.  KCNJ11 is underexpressed in human islets and ZDF rats islets and hypothalamus, which is consistent with impaired β-cell insulin release [90–93]. |  |

| 3.3.3 PPARG |  |
| --- | --- |
| Yes, partially.  PPARG is overexpressed in adipose tissue in diabetic humans and ZDF rats, but expression levels in the liver are conflicting: underexpression being reported in humans and overexpression in ZDF rats [41,93]. |  |

| **4. BIOCHEMICAL VALIDATION** | |
| --- | --- |
| 4.1 If there are known pharmacodynamic (PD) biomarkers related to the pathophysiology of the disease, are they also present in the model? | Score |
| Yes, completely.  Remarks: Glycaemic markers (blood glucose and HbA1c), cholesterol and triglycerides levels are directly related to the development of diabetes and diabetic complications, being used as PD biomarkers. | 3 |
| 4.1.1 Glycaemic markers (blood glucose, HbA1c) |  |
| All glycaemic markers can be measured in ZDF rats. |  |
| 4.1.2 Cholesterol |  |
| Cholesterol levels can be measured in ZDF rats. |  |
| 4.1.3 Triglycerides |  |
| Triglycerides levels can be measured in ZDF rats. |  |
| 4.2 Do these PD biomarkers behave similarly to humans’? | Score |
| Yes, completely. | 2 |
| 4.2.1 Glycaemic markers (blood glucose, HbA1c) |  |
| All glycaemic markers are increased in ZDF rats (see section 2.1.1). |  |
| 4.2.2 Cholesterol |  |
| Cholesterol levels are increased in ZDF rats (see section 2.1.1). |  |
| 4.2.3 Triglycerides |  |
| Triglycerides levels are increased in ZDF rats (see question 2.1.1). |  |
| 4.3 If there are known prognostic biomarkers related to the pathophysiology of the disease, are they also present in the model? | Score |
| Yes, completely.  Remarks: Glycaemic markers are also prognostic markers as higher glycaemic levels can potentially lead to faster worsening of the diabetic condition [94–96]. The same for cholesterol and triglycerides, which are directly involved in cardiovascular damage and diabetic complications [97,98]. | 3 |
| 4.3.1 Glycaemic markers (blood glucose, HbA1c) |  |
| All glycaemic markers can be measured in ZDF rats. |  |
| 4.3.2 Cholesterol |  |
| Cholesterol levels can be measured in ZDF rats. |  |
| 4.3.3 Triglycerides |  |
| Triglycerides levels can be measured in ZDF rats. |  |

| 4.4 Do these prognostic biomarkers behave similarly to humans’? | Score |
| --- | --- |
| Yes, completely.  Remarks: Like humans, ZDF rats have increased levels of blood glucose, cholesterol and triglycerides. | 2 |
| 4.4.1 Glycaemic markers (blood glucose, HbA1c) |  |
| All glycaemic markers are increased in ZDF rats (see section 2.1.1). |  |
| 4.4.2 Cholesterol |  |
| Cholesterol levels are increased in ZDF rats (see section 2.1.1). |  |
| 4.4.3 Triglycerides |  |
| Triglycerides levels are increased in ZDF rats (see question 2.1.1). |  |

| **5. AETIOLOGICAL VALIDATION** | |
| --- | --- |
| 5.1 Is the aetiology of the disease similar to humans’? | Score |
| Yes, partially.  Remarks: diabetes type 2 is strongly associated with obesity, high-fat/carbs diet and sedentary lifestyle [15,99]. Lifestyle changes such as a healthful diet, BMI control and reduction of smoking/alcohol intake could prevent as much as 90% of type diabetes cases [99,100].  In ZDF rats, diabetes is caused by the concomitant presence of two different genetic factors. The first is a mutation in the extracellular domain of the leptin receptor (*lepr*) gene. Leptin is a hormone that can be produced by various tissues, but mostly by mature adipocytes in white adipose tissue. It has an important role in satiety when it is taken up by the brain from the circulation [40]. The mutation in the leptin gene is called ‘fa’ because it was originally identified in the Zucker Fatty rats, from which the ZDF rat strain was derived [40]. ZDF rats are homozygous for this missense mutation in the leptin receptor from A to C at position 880, which causes a Gln to Pro change in all identified isoforms of the Ob-R protein, while lean littermates do not have it [40,101]. The second factor is an autosomal recessive defect in β-cell transcription, which is inherited independently from the *lepr* mutation and contributes directly to the manifestation of overt diabetes [102].  In humans, although leptin has a similar function, it seems to be less critical for the regulation of energy expenditure as inactivating mutations in its receptor have milder effects than in rodents [103]. Several genome-wide association studies (GWAS) have conflicting results regarding the association of polymorphisms in the *lepr* gene and risk of diabetes [104–106]. Nevertheless, the existence of genetic factors is widely accepted, although they probably result from the product of several small to moderate gene effects [41–48].  For overt diabetes to develop in humans, it is necessary to have a genetic predisposition, β-cell dysfunction and insulin resistance (usually acquired due to obesity). In that sense, ZDF rats are similar [102]. Male ZDF rats develop diabetes even on low-fat diets while females need high-fat diets to do so, the latter being closer to the common human aetiology [12]. This fact is also in line with the specific female resistance to the development of diabetes seen in humans, due to a possible protective effect of oestrogen on pancreatic beta-cells [13,14]. | 7 |

| **6. HISTOLOGICAL VALIDATION** | |
| --- | --- |
| 6.1 Do the histopathological structures in relevant tissues resemble the ones found in humans? | Score |
| Yes, partially.  There is some evidence of β-cell mass reduction and pathogenic role of amyloid deposits in human patients, even though conflicting results to these notions are also present [107–110]. Nonetheless, they were included to better characterise the models in this sheet. The pancreas was the only tissue selected for the histological validation because it primarily affects the pancreatic islets, in which long-term damage then leads to diabetic complications. | 5.5 |

| 6.1.1 Histopathological features modelled |  |
| --- | --- |
| Β-cell mass: as seen in diabetic patients, the β-cell mass in ZDF rats islets is severely diminished with the development of overt diabetes [22,111]. |  |
| 6.1.2 Histopathological features partially modelled |  |
| Islet morphology: islets become irregularly delineated with the advancement of diabetes, also increasing in size and presenting vacuolations between the mantles. Even though the basic morphology is different than the human pancreas, the presence of fibrosis and fat infiltration is similar. Vacuolations commonly present in ZDF rat pancreas tissue are present in humans only due to tissue processing errors [18,22,108,112–114]. |  |
| 6.1.3 Histopathological features not modelled |  |
| Amyloidosis: although there is evidence of hypersecretion of islet amyloid polypeptide (IAPP) or amylin in ZDF rats’ pancreas, there is no report of formation of the characteristic amyloid plaques as seen in humans [26,114,115]. |  |

| **7. PHARMACOLOGICAL VALIDATION** | |
| --- | --- |
| 7.1 Are effective drugs in humans also effective in this model? | Score |
| Yes, partially.  Remarks: one drug from each class approved by FDA and/or EMA for the treatment of type 2 diabetes was included. | 4.02 |
| 7.1.1 Amylin Agonist: Pramlintide |  |
| Methodology: studies were searched on PubMed with the string ‘("ZDF rat" OR "ZDF rats" OR "zucker diabetic fatty rat" OR "zucker diabetic fatty rats") AND pramlintide’ on 27/02/2017, yielding no results. |  |
| 7.1.2 Biguanide: Metformin [116–137] |  |
| Methodology: studies were searched on PubMed with the string ‘("ZDF rat" OR "ZDF rats" OR "zucker diabetic fatty rat" OR "zucker diabetic fatty rats") AND metformin’ on 27/02/2017, yielding 26 results which were screened by title and abstract. A study retrieved through the search string for rosiglitazone with a metformin monotherapy arm was also screened. Of these, twenty-two (22) articles were included in this section.  Results: most studies show a reduction of glycaemia measures, such as plasma glucose, HbA1c, OGTT; and triglycerides and free fatty acids. Studies which did not report a reduction in glycaemic parameters often cite dose as the probable reason for this finding. Metformin improves islet histology and increases β-cell total mass (increases expression of anti-apoptotic genes while decreasing pro-apoptotic ones) when compared to controls. It has also been shown to have a moderate effect on liver steatosis and to improve nephropathy by reducing genomic damage. However, it caused no improvement in the endothelial function. All but one article were classified as category II. Of the 21 studies classified as category II, fourteen (14) are in line with clinical findings.  Quality assessment: according to the pre-specified criteria and n = 22, 41% have information on housing; 95% on husbandry, 91% on sample size, none on sample size calculation nor blinding, 50% on randomisation; 59% on acclimatisation; and 95% on sex (19 used only male animals, one used only female animals and one used both). |  |

| 7.1.3 Bile Acid Sequestrant: Colesevelam [138] |  |
| --- | --- |
| Methodology: studies were searched on PubMed with the string ‘("ZDF rat" OR "ZDF rats" OR "zucker diabetic fatty rat" OR "zucker diabetic fatty rats") AND colesevelam’ on 27/02/2017, yielding 1 result which was screened by title and abstract. This article was included in this section.  Results: the only study shows a positive effect of colesevelam on reducing common glycaemic parameters such as fasting blood glucose and oral glucose tolerance test (OGTT). Colesevelam treatment improved had no effect on body weight nor on food intake, suggesting its action is exerted through increased levels of active GLP-1. It was also effective at reducing β-cell islets degeneration when compared to the control. This article was classified as category II. This study is in line with clinical findings.  Quality assessment: according to the pre-specified criteria and n = 1, the article has no information on housing nor husbandry, has information on sample size, has no information on sample size calculation, blinding, randomisation nor acclimatisation and has information on sex (all animals were male). |  |
| 7.1.4 Dopamine-2 Receptor Agonist: Bromocriptine |  |
| Methodology: studies were searched on PubMed with the string ‘("ZDF rat" OR "ZDF rats" OR "zucker diabetic fatty rat" OR "zucker diabetic fatty rats") AND bromocriptine’ on 27/02/2017, yielding no results. |  |
| 7.1.5 DPP-IV inhibitor: Sitagliptin [120,133,138–144] |  |
| Methodology: studies were searched on PubMed with the string ‘("ZDF rat" OR "ZDF rats" OR "zucker diabetic fatty rat" OR "zucker diabetic fatty rats") AND sitagliptin’ on 27/02/2017, yielding 12 results which were screened by title and abstract. Of these, ten (10) articles were included in this section.  Results: most studies show a positive effect on glycaemic control with sitagliptin significantly lowering plasma glucose, HbA1c and increasing insulin secretion. Two studies did not find any significant change in these outcomes. They also show an increase in aGLP-1 levels and a decrease in triglycerides. An increase in general β-cell function is also reported by the glucose/insulin ration and the HOMA-beta index. This is supported by no evidence of induction of pancreatitis and general improvement of islet histology (reduction of β-cell vacuolation, inflammation, fibrosis and apoptosis). Additionally, a reduction in the expression of IL-1β and genes involved in apoptosis induction was also reported. All articles were classified as category II. Eight (8) studies are in line with clinical findings.  Quality assessment: according to the pre-specified criteria and n = 10, 20% have information on housing; 80% on husbandry, 100% on sample size, none on sample size calculation nor blinding, 20% on randomisation; 40% on acclimatisation; and 90% on sex (all animals were male). |  |
| 7.1.6 GLP-1 agonist: Exenatide [112,145–151] |  |
| Methodology: studies were searched on PubMed with the string ‘("ZDF rat" OR "ZDF rats" OR "zucker diabetic fatty rat" OR "zucker diabetic fatty rats") AND exenatide’ on 27/02/2017, yielding 14 results which were screened by title and abstract. Of these, eight (8) articles were included in this section.  Results: all studies show a positive effect of exenatide on blood glucose and/or HbA1c. Exenatide was also able to increase insulin levels after feeding, indicating increased β-cell activity. Additionally, it reduced triglyceride levels in female ZDF rats and improved islet histology (e.g. reduced β-cell vacuolation). All articles were classified as category II. All studies are in line with clinical findings.  Quality assessment: according to the pre-specified criteria and n = 8, 25% have information on housing; 88% on husbandry, 100% on sample size, none on sample size calculation nor blinding, 25% on randomisation; 50% on acclimatisation; and 88% on sex (6 used only male animals and one used both). |  |
| 7.1.7 Metiglinide: Repaglinide |  |
| Methodology: studies were searched on PubMed with the string ‘("ZDF rat" OR "ZDF rats" OR "zucker diabetic fatty rat" OR "zucker diabetic fatty rats") AND repaglinide’ on 27/02/2017, yielding no results. The same string was used to search for studies replacing repaglinide by nateglinide, however, this search also returned no results. |  |

| 7.1.8 PPAR-γ agonist: Rosiglitazone [111,113,124,137,152–186] |  |
| --- | --- |
| Methodology: studies were searched on PubMed with the string ‘("ZDF rat" OR "ZDF rats" OR "zucker diabetic fatty rat" OR "zucker diabetic fatty rats") AND rosiglitazone’ on 27/02/2017, yielding 47 results which were screened by title and abstract. Of these, thirty-nine (39) articles were included in this section.  Results: most studies show a significant reduction in glycaemic (plasma glucose, HbA1c, OGTT) and lipidemic parameters (LDL, free fatty acids (FFA), total cholesterol and triglycerides). Some studies in which no effect on glycaemic control was achieved were performed in older animals, in which β-cell degeneration is already at an advanced stage and this reason was cited as a possible cause for the lack of efficacy. Rosiglitazone reduced insulin levels during the hyperinsulinemic phase and increased them during the hypoinsulinemic phase. It also increased HOMA-IR and insulin sensitivity indexes, improved islet morphology (increased islet area and insulin content). Many studies showed a considerable reduction of food intake with an increase in body weight. Furthermore, rosiglitazone could reverse the metabolic phenotype of the diabetic heart, reduce the number of apoptotic cardiomyocytes and infarction size. It can normalise gene expression in the liver and decrease hepatic fat. It has also been showed to improve nephropathy by decreasing kidney weight, hypertrophy and urinary glucose. Although according to one study it can restore endothelium-dependent vasorelaxation, it had no effect on endothelium’s mechanical properties. All articles but one were classified as category II. Of the 38 category II studies, thirty-two (32) are in line with clinical findings.  Quality assessment: according to the pre-specified criteria and n = 39, 46% have information on housing; 82% on husbandry, 90% on sample size, none on sample size calculation nor blinding, 41% on randomisation; 46% on acclimatisation; and 90% on sex (32 used only male animals, one used only female animals and 2 used both). |  |
| 7.1.9 SGLT-2 inhibitor: Canagliflozin [139,187–192] |  |
| Methodology: studies were searched on PubMed with the string ‘("ZDF rat" OR "ZDF rats" OR "zucker diabetic fatty rat" OR "zucker diabetic fatty rats") AND canagliflozin’ on 27/02/2017, yielding 7 results which were screened by title and abstract. Of these, six (6) articles were included in this section.  Results: all studies show a positive effect of canagliflozin on plasma glucose, HbA1c and glucose AUC in OGTT tests. Some studies also report an increase in plasma insulin levels and aGLP-1, although there are conflicting results regarding aGLP-1. Canagliflozin was able to restore insulin immunoreactivity in ZDF rat islets back to similar levels as the lean controls. Furthermore, it reduced β-cell vacuolation and increase β-cell general activity measured by glucose/insulin ratio following an OGTT. All articles were classified as category II. All studies are in line with clinical findings.  Quality assessment: according to the pre-specified criteria and n = 6, 17% have information on housing; 83% on husbandry, 100% on sample size, none on sample size calculation, blinding nor randomisation; 50% on acclimatisation; and 100% on sex (all animals were male). |  |
| 7.1.10 Sulphonylurea: Glibenclamide [117,193] |  |
| Methodology: studies were searched on PubMed with the string ‘("ZDF rat" OR "ZDF rats" OR "zucker diabetic fatty rat" OR "zucker diabetic fatty rats") AND (glibenclamide OR glyburide)’ on 27/02/2017, yielding six (6) results which were screened by title and abstract. Of these, two (2) articles were included in this section.  Results: one study showed a positive effect of glibenclamide on OGTT, lowering the glucose ^AUC^_0-4h_ when compared to control. However, the other study showed no effect on blood glucose, insulin or HbA1c levels besides no improvement on islet histology. Both articles were classified as category II. Only one study is in line with clinical findings.  Quality assessment: according to the pre-specified criteria and n = 2, 50% have information on housing, 100% on husbandry, 50% sample size, none on sample size calculation nor blinding, 50% on randomisation, none on acclimatisation; and 100% sex (all animal were male). |  |

| 7.1.11 α-glucosidase Inhibitor: Acarbose [37,194–196] |  |
| --- | --- |
| Methodology: studies were searched on PubMed with the string ‘("ZDF rat" OR "ZDF rats" OR "zucker diabetic fatty rat" OR "zucker diabetic fatty rats") AND acarbose’ on 27/02/2017, yielding 4 results which were screened by title and abstract. All four articles were included in this section.  Results: all studies show a positive effect of acarbose on reducing blood glucose and HbA1c. Acarbose has also been shown to reduce basement membrane thickening with a modest effect on cell density in ZDF retinopathy. It can also reduce free fatty-acid levels in the blood as well as cholesterol. All articles were classified as category II. All studies are in line with clinical findings.  Quality assessment: according to the pre-specified criteria and n = 4, 25% have information on housing, 75% on husbandry, 75% on sample size, none on sample size calculation nor blinding, 25% on randomisation, 25% on acclimatisation; and 75% on sex (all animal were male). |  |
| 7.2 Are ineffective drugs in humans also ineffective in this model? | Score |
| Of the six identified classes of ineffective drugs, only two have been tested in ZDF rats, none being in line with the clinical findings. The efficacy of the other five classes in this model remains unclear. | 0.10 |
| 7.2.1 11-Beta hydroxysteroid dehydrogenase inhibitors |  |
| Methodology: studies were searched on PubMed with the string ‘("ZDF rat" OR "ZDF rats" OR "zucker diabetic fatty rat" OR "zucker diabetic fatty rats") AND ("11-beta hydroxysteroid dehydrogenase inhibitor" OR "11-beta hydroxysteroid dehydrogenase inhibitors" OR "11 beta-HSD" OR "11βHSD")’ on 27/02/2017, yielding no results. |  |
| 7.2.2 Adenosine A1 receptor agonists |  |
| Methodology: studies were searched on PubMed with the string ‘("ZDF rat" OR "ZDF rats" OR "zucker diabetic fatty rat" OR "zucker diabetic fatty rats") AND "Adenosine A1 receptor agonist" OR "Adenosine A1 receptor agonists" OR AA1RA’ on 27/02/2017, yielding 1 result which was screened by title and abstract. This article was included in this section. |  |
| 7.2.2.1 CVT-3619 [197] |  |
| Results: the only study in ZDF rats showed CVT-3619 reduced FFA levels and inhibited lipolysis. This article was classified as category I.  Quality Assessment: according to the pre-specified criteria and n = 1, the article has information on housing, on husbandry, on sample size, no information on sample size calculation, blinding, randomisation nor acclimatisation; and has information on sex (all animals were male). |  |
| 7.2.3 Nicotinic α-7 receptor agonists |  |
| Methodology: studies were searched on PubMed with the string ‘("ZDF rat" OR "ZDF rats" OR "zucker diabetic fatty rat" OR "zucker diabetic fatty rats") AND "nicotinic α-7 receptor" OR "Nicotinic α-7 receptor agonist" OR "α7NR"’ on 27/02/2017, yielding no results. |  |
| 7.2.4 TGR5 receptor agonists |  |
| Methodology: studies were searched on PubMed with the string ‘("ZDF rat" OR "ZDF rats" OR "zucker diabetic fatty rat" OR "zucker diabetic fatty rats") AND ("G protein-coupled bile acid receptor 1 agonist" OR GPBAR1) ("TGR5 receptor" OR Gpbar1 OR M-BAR OR GPR131 OR BG37 OR Axor109)’ on 27/02/2017, yielding 1 result which was screened by title and abstract. This article was not included in this section. |  |
| 7.2.5 Protein tyrosine phosphatase 1B inhibitors |  |
| Methodology: studies were searched on PubMed with the string ‘("ZDF rat" OR "ZDF rats" OR "zucker diabetic fatty rat" OR "zucker diabetic fatty rats") AND (PTP1B OR "protein tyrosine phosphatase 1B inhibitor" OR "protein tyrosine phosphatase 1B inhibitors")’ on 27/02/2017, yielding 1 result which was screened by title and abstract. This article was not included in this section. |  |

| 7.2.6 Fructose-1,6-bisphosphatase inhibitors |  |
| --- | --- |
| Methodology: studies were searched on PubMed with the string ‘("ZDF rat" OR "ZDF rats" OR "zucker diabetic fatty rat" OR "zucker diabetic fatty rats") AND ("fructose-1,6-bisphosphatase inhibitor" OR "fructose-1,6-bisphosphatase inhibitors" OR "FBPase inhibitor" OR "FBPase inhibitors")’ on 27/02/2017, yielding 5 results which were screened by title and abstract. Of these, three (3) articles were included in this section. |  |
| 7.2.6.1 CS-917 [198–200] |  |
| Results: all three studies showed a significant reduction in plasma glucose levels and inhibition of gluconeogenesis. All articles were classified as category II. None of these studies is in line with the clinical findings.  Quality Assessment: according to the pre-specified criteria and n = 3, no articles have information on housing, 67% have information on husbandry, 100% on sample size, none on sample size calculation nor blinding, 33% on randomisation, 33% on acclimatisation; and 100% on sex (2 used only male animals and one used both sexes). |  |
| 7.3 Have drugs with different mechanisms of action and acting on different pathways been tested in this model? If so, which? | Score |
| Yes, partially.  Remarks: out of the 17 identified drug classes tested and/or used to treat type 2 diabetes, 10 were tested in ZDF rats. | 1.18 |

| **8. ENDPOINT VALIDATION** | |
| --- | --- |
| 8.1 Are the endpoints used in preclinical studies the same or translatable to the clinical endpoints? | Score |
| Yes.  Remarks: most studies performed in ZDF rats with agents to treat type 2 diabetes have used glycaemic parameters (e.g. glycaemia, HbA1c, OGTT) or other measures of insulin sensitivity. These measurements also often represent the primary outcomes of trials testing new drugs for the treatment of type 2 diabetes, which aim to control glycaemia. | 8 |
| 8.2 Are the methods used to assess preclinical endpoints comparable to the ones used to assess related clinical endpoints? | Score |
| Yes.  Remarks: the biochemical methods used in the preclinical studies are the same or similar to the ones used to measure glycaemic parameters in humans. | 3 |

| **MODEL NAME** | db/db mouse (BKS.Cg-Dock7^m^ +/+ Lepr *db*/J*)* |
| --- | --- |
| **INDICATION** | Type 2 Diabetes Mellitus (T2D) |
| **VALIDATION DATE** | 10.07.2017 |
| **TOTAL SUBSECTIONS** | 59 |
| **TOTAL SCORE** | 67.36 |
| **UNCERTAINTY FACTOR (%)** | 23.7 |
| **HISTORICAL BACKGROUND** | |
| In 1966 at Jackson Laboratories, a mutation in the inbred mouse strain C57BL/Ks occurred which caused a metabolic syndrome similar to the human diabetes mellitus [201]. The mice manifested hyperglycaemia, hyper- then hypoinsulinemia and histological changes in the pancreatic islets. This is an autosomal recessive mutation and the strain was called db/db mouse (recessive diabetic) in a similar fashion to ob/ob (recessive obese) mouse [201]. Both strains have mutations in the leptin pathway, the db/db affecting the leptin receptor while the ob/ob affects leptin itself. This nomenclature replaced the old ‘Ob-protein and Ob-receptor’ used before to differentiate both strains. The misty mutation (*Dock7^m^*) was introduced to maintain the diabetes gene as homozygotes for the Lepr^db^ mutation are infertile besides helping in the identification of homo- and heterozygotes by colour changes in the fur [202]. The db/db mouse is commercialised with two different backgrounds: C57BL/6 (resistant to the development of diabetes) and C57BL/Ks (susceptible), which also known as C57BL/KsJ [202–206]. | |

| **1. EPIDEMIOLOGICAL VALIDATION** | |
| --- | --- |
| 1.1 Is the model able to simulate the disease in the relevant sexes? | Score |
| Yes, completely.  Remarks: both male and female db/db mice can develop diabetes. The diabetic condition is somewhat more pronounced in male than in female mice [201,207]. This specific female resistance to the development of diabetes is in line with what is seen in humans, due to a possible protective effect of oestrogen on pancreatic beta-cells [13,14]. | 2 |
| 1.2 Is the model able to simulate the disease in the relevant age groups (juvenile, adult or ageing)? | Score |
| Yes, partially.  Remarks: Although the time to onset of diabetes has been decreasing in the past years, the db/db mouse still develops diabetes somewhat earlier than humans [15,202]. However, it progresses into adulthood and ageing phases, similarly to the human disease [208–212]. | 1 |

| **2. SYMPTOMATOLOGY AND NATURAL HISTORY (SNH) VALIDATION** | |
| --- | --- |
| 2.1 Is the model able to replicate the symptoms commonly present in this disease? If so, which ones? | Score |
| Yes, completely.  The most common symptoms of diabetes type 2 are polyphagia, polydipsia, polyuria, weight loss, fatigue, healing impairment and obesity [21]. The latter may not be considered a symptom per se, is better classified as a co-morbidity. However, due to the high prevalence of this condition in T2D patients and likely association to the pathophysiology, it was also included in this section.  Most of these symptoms are directly connected to the glycaemia and dyslipidaemia and therefore they were translated into the following parameters: hyperglycaemia, hyperinsulinemia followed by hypoinsulinemia, dyslipidaemia (divided into hypercholesterolemia and hypertriglyceridemia according to the common human pathological profile) and obesity. | 13 |

| 2.1.1 Symptoms modelled |  |
| --- | --- |
| Hyperglycaemia: db/db mice have increased blood glucose levels of up to 500% normal levels [201,203–205,208,209,211,213,214]. |  |
| Hyperinsulinemia followed by hypoinsulinemia: insulin levels are increased in db/db mice as early as 10 days of age. Over time, with extenuation of β-cells, insulin levels drop to normal or below normal [203,206–209,211,214,215]. |  |
| Hypercholesterolemia: cholesterol levels are significantly increased in db/db mice starting at 5 weeks of age [212,216]. |  |
| Hypertriglyceridemia: triglycerides levels are higher in db/db mice than in controls of the same background, starting with a moderate increase at 5 weeks and progressively increasing with age up to 19 weeks [212,216]. |  |
| Obesity: db/db mice are significantly heavier than their lean littermates at a young age, but tend to significantly lose weight after overt diabetes, which is consistent with the sudden weight loss sometimes seen in humans [201,205,208,209,214]. |  |
| 2.2 Is the natural history of the disease similar to humans regarding: |  |
| 2.2.1 Time to onset; | Score |
| No.  Remarks: type 2 diabetes is usually diagnosed around 40 years, although age at diagnosis has been decreasing [15]. The onset of diabetes in db/db mice may vary from 4 to 8 weeks [202]. This happens at quite an early stage of life when compared to humans: mice 4 weeks of age are in a similar stage of development as humans between 6 months and 10 years old while mice at 8 weeks correspond to a human at around 20 years [217,218]. | 0 |
| 2.2.2 Disease progression; | Score |
| Yes, partially.  Remarks: Diabetes progression in db/db mice follows much of the human disease. The increase in sugar/fat intake in diets leads to an increase in insulin secretion, reducing the ability to activate the TK receptor (between 4 and 8 weeks of age). Consequently, this then leads to an increase in glycaemia and β-cell degeneration, finally leading to β-cell apoptosis and glucose intolerance [208–212]. The whole process, however, happens at quite an early stage of life when compared to humans: mice 4 weeks of age are in a similar stage of development as humans between 6 months and 10 years old while mice at 8 weeks correspond to a human at around 20 years [217,218]. Although as mentioned before, the age of onset of diabetes has been decreasing in the past years [15].  As for common diabetic complications, such as nephropathy, retinopathy and neuropathy, db/db mice can partially model them. They are able to develop renal lesions (such as albuminuria, glomerular basement thickening, loss of podocytes and moderate mesangial matrix expansion) but fail to reproduce mesangiolysis, nodular mesangial sclerosis and severe tubulointerstitial fibrosis, not developing progressive renal insufficiency [219–224]. Regarding retinopathy, some characteristics such as pericyte loss, degeneration of the blood-retinal barrier (BRB), apoptosis of neuro-retinal cells, thickening of capillary basement membranes, glial reactivation and vascular proliferation are present while it cannot model retinal neovascularization [225–230]. Likewise, db/db mice can partially develop neurological complications, such as decreased motor nerve conduction velocity (MNCV), the absence of large myelinated fibres, axonal atrophy and dystrophy (myelinated and unmyelinated fibres); and degeneration of myelin sheath, but do not develop neurotic dystrophy [231–235]. | 1 |
| 2.2.3 Duration of symptoms; | Score |
| Yes, completely.  Remarks: like humans, once insulin resistance and glucose intolerance are set, β-cell apoptosis follows, worsening the diabetic condition progressively and leading to overt diabetes and diabetic complications [201,206,208]. | 2 |

| 2.2.4 Severity. | Score |
| --- | --- |
| Yes, partially.  Remarks: the biochemical parameters in db/db mice are similar to humans in severity, is considerably increased. However, the complications that follow after the onset of overt diabetes are somewhat milder in db/db mice (e.g. lack of mesangiolysis and tubulointerstitial fibrosis in nephropathy; neovascularisation in retinopathy or neurotic dystrophy in neuropathy). | 1 |

| **3. GENETIC VALIDATION** | |
| --- | --- |
| 3.1 Does this species also have orthologous genes and/or proteins involved in the human disease? If so, which? | Score |
| Yes, completely.  Although is widely accepted that diabetes type 2 has genetic factors involved, reliable tracking and identification of such genes remain a challenge [41–48]. This is often referred to as the ‘missing heritability’ [42]. In some cases, T2D can be caused by a single gene (such as mutations on HNF4A for Maturity-Onset-Diabetes of the Young type 1 – MODY1 – or PTF1A for neonatal diabetes) [41,43]. Nonetheless, in most cases it is a polygenic and heterogeneous disease, meaning multiple genes might be involved and different combinations of polymorphisms can lead to the pathological phenotype, which makes the genetic validation of animal models at best extremely challenging [41,43,45–48].  With the advent of Genome-Wide Association Studies (GWAS), many genes have been associated with an increased risk of developing type 2 diabetes, shedding light on the complex genetic architecture of the disease [41,49,50]. However, three of them are better established as likely genetic factors: TCF7L2, KCNJ11 and PPARG [41,44,51]. Therefore, these were the only ones included in this section.  All three genes are present in db/db mice [236–238]. | 8 |
| 3.1.1 TCF7L2 |  |
| TCF7L2 is a gene (also known as also known as TCF-4 or β-catenin interacting protein) located at 10q25.2-q25.3 in humans and at 1q55 in rats that codes a transcription factor involved in the WNT signalling pathway [52,55–58]. This is the strongest and most well-replicated genetic factor associated to T2D [41,44,49,50,59,60]. It acts as a nuclear receptor for β-catenin, which is involved in the secretion of GLP-1 in gut endocrine cells and various other genes [56,57,61]. There are also reports that indicate a possible role in the incretin axis, adipocyte function and glucose production by the liver, despite some of the results being conflicting [58,62–66]. |  |
| 3.1.2 KCNJ11 |  |
| KCNJ11 is a gene (also known as KIR6.2) located at 11p15.1 in humans and at 1q22 in rats that has been associated with an increased risk of developing T2D [49–51,54,67]. It codes for a major subunit of the ATP-sensitive K^+^ channel, an inward-rectifier potassium ion channel present in the pancreatic islets with direct influence over insulin secretion [68,69]. Mutations in this gene are connected to neonatal diabetes [41]. |  |
| 3.1.3 PPARG |  |
| PPARG is a gene that codes for the transcription factor peroxisome proliferator-activated receptor-γ (PPARγ) located at 3p25.2 and at 4q42 in rats in humans [53,70]. The PPARG is involved in adipocyte regulation, fat accumulation and glucose metabolism and it is stimulated by insulin in a ligand-dependent manner [71–74]. Two isoforms exist in humans: PPARγ1 and PPARγ2, the first being expressed in most tissues and the second one mostly in the liver and adipose tissue [75]. The isoform 2 is also the target of thiazolidinediones, such as rosiglitazone. |  |

| 3.2 If so, are the relevant genetic mutations or alterations also present in the orthologous genes/proteins? |  |
| --- | --- |
| Unclear. | 0.5 |
| 3.2.1 TCF7L2 |  |
| Unclear.  In humans, the rs12255372 and rs7903146 alleles are the most strongly associated variants with carriers of such type having reduced insulin secretion but not increased insulin resistance [76]. Being homozygous for the high-risk allele doubles the chance of developing T2D [41]. No studies investigating specific genetic alterations in this gene could be found in PubMed in db/db mice using the string ("db/db mouse" OR " db/db mice") AND TCF7L2 on 05/07/2017. |  |
| 3.2.2 KCNJ11 |  |
| Unclear.  In humans, the commonest polymorphism is a glutamate to lysine substitution at position 23 (E23K or rs5219) [77–80]. This mutation is linked to a reduction in channel sensitivity, increasing the signal threshold for the release of insulin and impairing serum insulin response [68,77,78,81]. Homozygous carriers of this mutation (KK) have an almost double risk of developing T2D when compared to non-carriers [79].  No studies investigating specific genetic alterations in this gene could be found in PubMed in db/db mice using the string ("db/db mouse" OR " db/db mice") AND (KCNJ11 OR KIR6.2 OR “KIR 6.2”) on 05/07/2017. |  |
| 3.2.3 PPARG |  |
| Unclear.  In humans, the SNP rs1801282 consists of a proline substitution for alanine in position 12 (Pro12Ala or P12A) of PPARγ2 (the other isoform is not affected), the proline allele is associated with an increased risk of developing T2D, higher BMI and decreased insulin sensitivity while the alanine one confers resistance [49–51,79,80,82–84]. Even though the mechanism is still unclear, the Ala variant has a lower transactivation  Efficiency, reducing stimulation of PPARG target genes and therefore also reducing the levels of adipose tissue mass accumulation [82]. Nonetheless, there are also reports of increased risk of cardiovascular disease in carriers of this polymorphism [85,86].  No studies investigating specific genetic alterations in this gene could be found in db/db mice in PubMed using the string ("db/db mouse" OR " db/db mice") AND PPARg on 05/07/2017. |  |
| 3.3 If so, is the expression of such orthologous genes and/or proteins similar to the human condition? | Score |
| Unclear. | 0.2 |
| 3.3.1 TCF7L2 |  |
| Unclear.  Two studies found the TCF7L2 gene is overexpressed in carriers of the risk genotype in pancreatic islets of T2D patients, but no changes in TCF7L2 expression were found by study of the Diabetes Genome Anatomy Project (DGAP) [41,62,87]. One study by Shu and colleagues showed a possible protective effect on the islets against glucose- and cytokine-induced apoptosis and function impairment. This study was later retracted due to image duplication and concerns over data reliability [88]. Two studies analysed expression of TCF7L2 in db/db mice in two different tissues: fat tissue and islets. TCF7L2 expression was increased in fat tissue of db/db mice, similarly to what is reported in humans while in islets, it is decreased [63,239,240]. |  |
| 3.3.2 KCNJ11 |  |
| Unclear.  KCNJ11 is underexpressed in human islets, which is consistent with impaired β-cell insulin release [92,93]. Nevertheless, no studies investigating expression levels of KCNJ11 could be found in db/db mice using the string ("db/db mouse" OR " db/db mice") AND (KCNJ11 OR KIR6.2 OR “KIR 6.2”) on 07/07/2017. |  |

| 3.3.3 PPARG |  |
| --- | --- |
| Unclear.  PPARG is overexpressed in adipose tissue and underexpressed in the liver of diabetic humans [41]. In db/db mice, during endotoxemia PPARG is underexpressed in White Adipose Tissue (WAT) [241]. A knockout mouse of Wdr13 with C57BL6 or db/db background significantly increases expression of PPARG in fat tissue [242]. A study of gene expression variation in db/db mice during the day showed significantly increased PPARG expression levels in the aorta during certain periods but did not analyse adipose tissue, islets or liver [243]. No studies specifically assessing PPARG expression in adipose tissue and liver could be found using the string ("db/db mouse" OR " db/db mice") AND PPARG on 07/07/2017. |  |

| **4. BIOCHEMICAL VALIDATION** | |
| --- | --- |
| 4.1 If there are known pharmacodynamic (PD) biomarkers related to the pathophysiology of the disease, are they also present in the model? | Score |
| Yes, completely.  Remarks: Glycaemic markers (blood glucose and HbA1c), cholesterol and triglycerides levels are directly related to the development of diabetes and diabetic complications, being used as PD biomarkers. | 3 |
| 4.1.1 Glycaemic markers (blood glucose, HbA1c) |  |
| All glycaemic markers can be measured in db/db mice. |  |
| 4.1.2 Cholesterol |  |
| Cholesterol levels can be measured in db/db mice. |  |
| 4.1.3 Triglycerides |  |
| Triglycerides levels can be measured in db/db mice. |  |
| 4.2 Do these PD biomarkers behave similarly to humans’? | Score |
| Yes, completely. | 2 |
| 4.2.1 Glycaemic markers (blood glucose, HbA1c) |  |
| All glycaemic markers are increased in db/db mice (see section 2.1.1). |  |
| 4.2.2 Cholesterol |  |
| Cholesterol levels are increased in db/db mice (see section 2.1.1). |  |
| 4.2.3 Triglycerides |  |
| Triglycerides levels are increased in db/db mice (see section 2.1.1). |  |
| 4.3 If there are known prognostic biomarkers related to the pathophysiology of the disease, are they also present in the model? | Score |
| Yes, completely.  Remarks: Glycaemic markers are also prognostic markers as higher glycaemic levels can potentially lead to faster worsening of the diabetic condition [94–96]. The same for cholesterol and triglycerides, which are directly involved in cardiovascular damage and diabetic complications [97,98]. | 3 |
| 4.3.1 Glycaemic markers (blood glucose, HbA1c) |  |
| All glycaemic markers can be measured in db/db mice. |  |
| 4.3.2 Cholesterol |  |
| Cholesterol levels can be measured in db/db mice. |  |
| 4.3.3 Triglycerides |  |
| Triglycerides levels can be measured in db/db mice. |  |

| 4.4 Do these prognostic biomarkers behave similarly to humans’? | Score |
| --- | --- |
| Yes, completely.  Remarks: Like humans, db/db mice have increased levels of blood glucose, cholesterol and triglycerides. | 2 |
| 4.4.1 Glycaemic markers (blood glucose, HbA1c) |  |
| All glycaemic markers are increased in db/db mice (see section 2.1.1). |  |
| 4.4.2 Cholesterol |  |
| Cholesterol levels are increased in db/db mice (see section 2.1.1). |  |
| 4.4.3 Triglycerides |  |
| Triglycerides levels are increased in db/db mice (see question 2.1.1). |  |

| **5. AETIOLOGICAL VALIDATION** | |
| --- | --- |
| 5.1 Is the aetiology of the disease similar to humans’? | Score |
| Yes, partially.  Remarks: diabetes type 2 is strongly associated with obesity, high-fat/carbs diet and sedentary lifestyle [15,99]. Lifestyle changes such as a healthful diet, BMI control and reduction of smoking/alcohol intake could prevent as much as 90% of type diabetes cases [99,100].  Diabetes is caused in the db/db mouse by a point mutation (Gly → Thr) in the OB-R (leptin receptor) sequence located at chromosome 4 [244,245]. The lack of signalling promotes the hypersecretion of circulating leptin, but the defective receptor prevents it from properly regulating the size of the body fat depot [40]. This was first suggested in several parabiosis experiments in which controls and diabetic mice (ob and db) had their circulatory systems connected. Ob/ob mice were able to lose weight and reduce food intake, indicating the mutation interfered with the action of a circulating factor (leptin) while db/db mice did not have any improvement, indicating its mutation affected the signal reception (leptin receptor) [213,246–248]. This hypothesis is further supported by the lack of response of db/db mouse to recombinant leptin [249–252].  In humans, although leptin has a similar function, it seems to be less critical for the regulation of energy expenditure as inactivating mutations in its receptor have milder effects than in rodents [103]. Several genome-wide association studies (GWAS) have conflicting results regarding the association of polymorphisms in the Lepr gene and risk of diabetes [104–106]. Nevertheless, the existence of genetic factors is widely accepted, although they probably result from the product of several small to moderate gene effects [41–48].  For overt diabetes to develop in humans, it is necessary to have a genetic predisposition, β-cell dysfunction and insulin resistance (usually acquired due to obesity). In that sense, db/db mice can only partially reproduce this dysfunction as diet restriction only slightly improves their condition [214]. Male db/db mice also have a somewhat more severe manifestation of diabetes than female mice. This fact is also in line with the specific female resistance to the development of diabetes seen in humans, due to a possible protective effect of oestrogen on pancreatic beta-cells [13,14]. | 7 |

| **6. HISTOLOGICAL VALIDATION** | |
| --- | --- |
| 6.1 Do the histopathological structures in relevant tissues resemble the ones found in humans? | Score |
| Yes, partially.  There is some evidence of β-cell mass reduction and pathogenic role of amyloid deposits in human patients, even though conflicting results to these notions are also present [107–110]. Nonetheless, they were included to better characterise the models in this sheet. The pancreas was the only tissue selected for the histological validation because it primarily affects the pancreatic islets, whose long-term damage then leads to diabetic complications. | 5.5 |

| 6.1.1 Histopathological features modelled |  |
| --- | --- |
| Β-cell mass: as seen in diabetic patients, the β-cell mass in db/db mice islets is severely diminished with the development of overt diabetes [201,209–211]. |  |
| 6.1.2 Histopathological features partially modelled |  |
| Islet morphology: islets become irregularly delineated with the advancement of diabetes, showing signs of hypertrophy, mixtures of acinar and islet cells and partial or total degranulation and necrosis of β-cells [201,204,209–211]. |  |
| 6.1.3 Histopathological features not modelled |  |
| Amyloidosis: islet amyloid polypeptide (IAPP) or amylin start increasing at 8 weeks but decrease significantly at 24 weeks. Nonetheless, there is no report of formation of the characteristic amyloid plaques as seen in humans [253]. |  |

| **7. PHARMACOLOGICAL VALIDATION** | |
| --- | --- |
| 7.1 Are effective drugs in humans also effective in this model? | Score |
| Yes, partially.  Remarks: one drug from each class approved by FDA and/or EMA for the treatment of type 2 diabetes was included. Studies that used db/db mice with C57BL/6 background (also known as B6.BKS(D)-Lepr db/J, #00697 at Jackson Laboratories) were excluded. In consultation with the main provider of this model, Jackson Laboratories, the following assumptions/decisions were made: studies that used db/db mice with C57BLK6/J or C57BLK/6 background were considered to have used the C57BL/6 background and therefore were also excluded. Studies with mice of C57BL/KsOlaHsd-Lepr and C57BLKS/J lar m+/Lepr^db^ were also excluded as it is likely that a mutation was engineered into the strain. A C57BL/KFJ background was assumed to be a C57BL/KsJ background (likely typo) as the references cited referred to this strain. Additionally, studies that did not disclose which background strain was used (i.e. just mentioned db/db mice) were included but were flagged with a ‘missing background’ note. If a study disclosed the control’s strain but not specifically the db/db mouse’s, it was assumed the diabetic mice had the same background as the control. | 3.88 |
| 7.1.1 Amylin Agonist: Pramlintide |  |
| Methodology: studies were searched on PubMed with the string ‘("db/db mouse” OR "db/db mice") AND pramlintide’ on 10/07/2017, yielding no results. |  |

| 7.1.2 Biguanide: Metformin [254–287] |  |
| --- | --- |
| Methodology: studies were searched on PubMed with the string ‘("db/db mouse" OR "db/db mice") AND metformin’ on 10/07/2017, yielding 68 results which were screened by title and abstract. A study retrieved through the search string for rosiglitazone with a metformin monotherapy arm was also screened. Of these, thirty-four (34) articles were included in this section.  Results: most studies show a reduction of glycaemia measures, such as plasma glucose, HbA1c, OGTT upon administration of metformin. Frequently, metformin has been shown to reduce plasma insulin levels during the hyperinsulinemic phase, although such benefit was not reported consistently. Some studies also report an improvement on the lipidic profile (reduction of LDL and FFA; and increase of HDL) and reduction of body weight and food intake. However, these results are often conflicting with other studies showing no effect at all on these parameters. A reduction of HOMA-IR is also often reported, including improvement on islet histopathology (e.g. reduced loss of islet boundaries and vacuolar degeneration). In the few studies which investigated metformin’s effect on diabetic retino- and nephropathy, it failed to show any significant improvement. Two (2) articles were classified as category I and thirty-two (32) as category II. Of the latter, twenty-eight (28) studies are in line with clinical findings.  Quality assessment: according to the pre-specified criteria and n = 34, 29% have information on housing, 79% on husbandry, 94% on sample size, none on sample size calculation nor blinding, 44% on randomisation, 41% on acclimatisation, 88% on sex (31 used only male animals and 3 only female animals); and 76% on background strain. |  |

| 7.1.3 Bile Acid Sequestrant: Colesevelam |  |
| --- | --- |
| Methodology: studies were searched on PubMed with the string ‘("db/db mouse" OR "db/db mice") AND cholestyramine’ on 10/07/2017, yielding 2 results which were screened by title and abstract. None of these articles was included in this section. |  |
| 7.1.4 Dopamine-2 Receptor Agonist: Bromocriptine |  |
| Methodology: studies were searched on PubMed with the string ‘("db/db mouse" OR "db/db mice") AND bromocriptine’ on 10/07/2017, yielding 1 result. This article was not included in this section. |  |
| 7.1.5 DPP-IV inhibitor: Sitagliptin [288] |  |
| Methodology: studies were searched on PubMed with the string ‘("db/db mouse" OR "db/db mice") AND sitagliptin’ on 10/07/2017, yielding 5 results which were screened by title and abstract. Of these, one (1) article was included in this section.  Results: the only study included reports a significant reduction of blood glucose, triglycerides and LDL while an increase in HDL. Both glucose tolerance (OGTT) and HOMA index were improved after treatment with sitagliptin. This article was classified as category II. This study is in line with clinical findings.  Quality assessment: according to the pre-specified criteria and n = 1, this article has no information on housing, husbandry, sample size, sample size calculation, blinding, randomisation, acclimatisation nor sex; while it has information on background strain. |  |
| 7.1.6 GLP-1 agonist: Exenatide [289–308] |  |
| Methodology: studies were searched on PubMed with the string ‘("db/db mouse" OR "db/db mice") AND exenatide’ on 10/07/2017, yielding 60 results which were screened by title and abstract. Of these, twenty (20) articles were included in this section.  Results: most studies report a positive effect of exenatide on glycaemic parameters (blood glucose, HbA1c) and glucose tolerance. Administration of exenatide has also been shown to increase plasma insulin levels, improve the HOMA-IR and islet histology, increase β-cell mass and the count of insulin-positive cells. Additionally, it improved diabetic nephropathy (e.g. by reducing glomerular hypertrophy and mesangial matrix expansion) despite no evident effect on blood glucose. Some studies have also shown an improvement in the overall lipidic profile by reducing in free-fatty acids (FFA) and LDL levels while increasing HDL. Three (3) articles were classified as category I and seventeen (17) as category II. Of the latter, twelve (12) studies are in line with clinical findings.  Quality assessment: according to the pre-specified criteria and n = 20, 30% have information on housing, 65% on husbandry, 100% on sample size, none on sample size calculation nor blinding, 20% on randomisation, 10% on acclimatisation, 80% on sex (12 used only male animals and 4 only female animals); and 85% on background strain. |  |
| 7.1.7 Metiglinide: Nateglinide [309] |  |
| Methodology: studies were searched on PubMed with the string ‘("db/db mouse" OR "db/db mice") AND repaglinide’ on 10/07/2017, yielding no results. The same string was used to search for studies replacing repaglinide by nateglinide, ‘("db/db mouse" OR "db/db mice") AND nateglinide’), yielding 1 result. This article was included in this section.  Results: the only study did not show any significant effect on blood glucose, plasma nor pancreatic insulin. This article was classified as category II. This study is not in line with clinical findings.  Quality assessment: according to the pre-specified criteria and n = 1, this article has no information on housing nor husbandry, has information on sample size, none on sample size calculation, blinding, randomisation nor acclimatisation, has information on sex (all animal were male) and none on background strain was. |  |

| 7.1.8 PPAR-γ agonist: Rosiglitazone [163,179,264,268,281,283,310–398] |  |
| --- | --- |
| Methodology: studies were searched on PubMed with the string ‘("db/db mouse" OR "db/db mice") AND rosiglitazone’ on 10/07/2017, yielding 128 results which were screened by title and abstract. Of these, ninety-six (96) articles were included in this section. One likely relevant article was not included because it was not available via university’s library:  Nanayakkara et al, Current Pharmaceutical Design. 2013, 19(27):4839-4847.  Results: most studies show a significant reduction in glycaemic (plasma glucose, HbA1c, OGTT) parameters. Although the majority of studies report an improvement in lipidemic parameters (LDL, free fatty acids (FFA), total cholesterol and triglycerides), some show conflicting results with an increase in total cholesterol and LDL and a decrease in HDL. Rosiglitazone reduced and increased insulin levels during the hyperinsulinemic and hypoinsulinemic phases, respectively. It also improved HOMA-IR and insulin sensitivity indexes, including islet morphology (increased β-cell area and number of insulin-positive cells). Many studies showed a considerable increase in body weight and body weight gain. Rosiglitazone treatment has been shown to improve fibrosis and inflammatory infiltration in the liver. Additionally, some studies show a deleterious effect on cardiac gene expression, mostly by activation of pro-apoptotic genes, which is likely related to rosiglitazone’s cardiotoxicity. Sixteen (16) articles were classified as category I and eighty (80) as category II. Of the latter, seventy-eight (78) studies are in line with clinical findings.  Quality assessment: according to the pre-specified criteria and n = 96, 36% have information on housing, 82% on husbandry, 92% on sample size, none on sample size calculation, 1% on blinding, 35% on randomisation, 38% on acclimatisation, 91% on sex (75 used only male animals, 10 only female animals and 2 both sexes); and 75% on background strain. |  |
| 7.1.9 SGLT-2 inhibitor: Canagliflozin [190] |  |
| Methodology: studies were searched on PubMed with the string ‘("db/db mouse" OR "db/db mice") AND canagliflozin’ on 10/07/2017, yielding 1 result which was screened by title and abstract. This article was included in this section.  Results: the only study included shows a significant decrease in blood glucose of db/db mice treated with canagliflozin. No other parameters were analysed in this study in db/db mice. This article was classified as category II. This study in line with clinical findings.  Quality assessment: according to the pre-specified criteria and n = 1, the article has information on sex (all animals were male) and background strain while no information on housing, husbandry, sample size, sample size calculation, blinding, randomisation nor acclimatisation was present. |  |
| 7.1.10 Sulphonylurea: Glibenclamide [254,255,309,399–401] |  |
| Methodology: studies were searched on PubMed with the string ‘("db/db mouse" OR "db/db mice") AND (glibenclamide OR glybenclamide)’ on 10/07/2017, yielding nine (9) results which were screened by title and abstract. A study retrieved through the search string for acarbose with a glibenclamide monotherapy arm was also screened. Of these, seven (7) articles were included in this section.  Results: With a thin margin, most studies show a positive effect of glibenclamide treatment on plasma glucose and insulin. One study also reports benefits for the integrity of the blood-brain barrier including a reduction in influx and activation of apoptosis-related biomarkers such as caspase-3 while another reports an increase in irisin release and reduction of triglycerides levels. Three (3) studies do not report any effect of glibenclamide on blood glucose, HbA1C and/(n)or plasma insulin levels. All articles were classified as category II. Four (4) studies are in line with clinical findings.  Quality assessment: according to the pre-specified criteria and n = 7, 43% have information on housing, 57% on husbandry, 100% on sample size, none on sample size calculation nor blinding, 14% on randomisation, none on acclimatisation, 100% on sex (6 used only male animals and 1 used only female animals); and 57% on background strain. |  |

| 7.1.11 α-glucosidase Inhibitor: Acarbose [266,360,402–408] |  |
| --- | --- |
| Methodology: studies were searched on PubMed with the string ‘("db/db mouse" OR "db/db mice") AND acarbose’ on 10/07/2017, yielding 15 results which were screened by title and abstract. Of these, nine (9) articles were included in this section.  Results: most studies show a positive effect of acarbose in reducing blood glucose and/or HbA1c. One study reported no effect on blood glucose despite reductions in HbA1c and urinary glucose excretion; and amelioration of diabetic nephropathy. Conflicting results on body weight and food intake reduction are reported. Acarbose has been shown to improve wound healing and angiogenesis. Moreover, it significantly decreases sucrase-isomaltase (SI) complex expression. All articles were classified as category II. All studies but one are in line with clinical findings.  Quality assessment: according to the pre-specified criteria and n = 9, 67% have information on housing, 89% on husbandry, 100% on sample size, none on sample size calculation nor blinding, 44% on randomisation, 56% on acclimatisation, 100% on sex (8 used only male animals and one both sexes); and 100% on background strain. |  |
| 7.2 Are ineffective drugs in humans also ineffective in this model? | Score |
| The efficacy of the six identified drug classes in this model remains unclear. | 0.1 |
| 7.2.1 11-Beta hydroxysteroid dehydrogenase inhibitors |  |
| Methodology: studies were searched on PubMed with the string ‘("db/db mouse" OR "db/db mice") AND ("11-beta hydroxysteroid dehydrogenase inhibitor" OR "11-beta hydroxysteroid dehydrogenase inhibitors" OR "11 beta-HSD" OR "11βHSD")’ on 10/07/2017, yielding no results. |  |
| 7.2.2 Adenosine A1 receptor agonists: GS-9667/CVT-3619 |  |
| Methodology: studies were searched on PubMed with the string ‘("db/db mouse" OR "db/db mice") AND ("Adenosine A1 receptor agonist" OR "Adenosine A1 receptor agonists" OR AA1RA)’ on 10/07/2017, yielding no results. |  |
| 7.2.3 Nicotinic α-7 receptor agonists |  |
| Methodology: studies were searched on PubMed with the string ‘("db/db mouse" OR "db/db mice") AND ("nicotinic α-7 receptor" OR "Nicotinic α-7 receptor agonist" OR "α7NR")’ on 10/07/2017, yielding no results. |  |
| 7.2.4 TGR5 receptor agonists |  |
| Methodology: studies were searched on PubMed with the string ‘("db/db mouse" OR "db/db mice") AND ("TGR5 receptor" OR Gpbar1 OR M-BAR OR GPR131 OR BG37 OR Axor109)’ on 10/07/2017, yielding 5 results which were screened by title and abstract. No articles were included in this section. |  |
| 7.2.5 Protein tyrosine phosphatase 1B inhibitor |  |
| Methodology: studies were searched on PubMed with the string ‘("db/db mouse" OR "db/db mice") AND ("PTP 112" OR PTP112 OR PTP-112 OR ertiprotafib)’ on 10/07/2017, yielding 19 results which were screened by title and abstract. No articles were included in this section. |  |
| 7.2.6 Fructose bisphosphatase inhibitor/Gluconeogenesis inhibitor |  |
| Methodology: studies were searched on PubMed with the string ‘("db/db mouse" OR "db/db mice") AND ("fructose-1,6-bisphosphatase inhibitor" OR "fructose-1,6-bisphosphatase inhibitors" OR "FBPase inhibitor" OR "FBPase inhibitors")’ on 10/07/2017, yielding 3 results which were screened by title and abstract. No articles were included in this section. |  |
| 7.3 Have drugs with different mechanisms of action and acting on different pathways been tested in this model? If so, which? | Score |
| Yes, partially.  Remarks: out of the 17 identified drug classes tested and/or used to treat type 2 diabetes, 9 were tested in db/db mice. | 1.18 |

| **8. ENDPOINT VALIDATION** | |
| --- | --- |
| 8.1 Are the endpoints used in preclinical studies the same or translatable to the clinical endpoints? | Score |
| Yes.  Remarks: most studies performed in db/db mice with agents to treat type 2 diabetes have used glycaemic parameters (e.g. glycaemia, HbA1c, OGTT) or other measures of insulin sensitivity. These measurements also often represent the primary outcomes of trials testing new drugs for the treatment of type 2 diabetes, which aim to control glycaemia. | 8 |
| 8.2 Are the methods used to assess preclinical endpoints comparable to the ones used to assess related clinical endpoints? | Score |
| Yes.  Remarks: the biochemical methods used in the preclinical studies are the same or similar to the ones used to measure glycaemic parameters in humans. | 3 |

References

1. Gourgari E, Wilhelm EE, Hassanzadeh H, Aroda VR, Shoulson I. A comprehensive review of the FDA-approved labels of diabetes drugs: Indications, safety, and emerging cardiovascular safety data. J Diabetes Complications. 2017;31(12):1719–27.

2. van der Worp HB, Howells DW, Sena ES, Porritt MJ, Rewell S, O’Collins V, et al. Can Animal Models of Disease Reliably Inform Human Studies? PLoS Med [Internet]. 2010 Mar 30;7(3):1–8. Available from: https://doi.org/10.1371/journal.pmed.1000245

3. Pound P, Ebrahim S, Sandercock P, Bracken MB, Roberts I. Where is the evidence that animal research benefits humans? BMJ Br Med J. 2004;328(7438):514–7.

4. Perel P, Roberts I, Sena E, Wheble P, Briscoe C, Sandercock P, et al. Comparison of treatment effects between animal experiments and clinical trials: systematic review. BMJ Br Med J [Internet]. 2007;334(7586):197–197. Available from: http://www.bmj.com/cgi/doi/10.1136/bmj.39048.407928.BE

5. Bebarta V, Luyten D, Heard K. Emergency medicine animal research: Does use of randomization and blinding affect the results? Acad Emerg Med. 2003;10(6):684–7.

6. Peers IS, South MC, Ceuppens PR, Bright JD, Pilling E. Can you trust your animal study data? Nat Rev Drug Discov [Internet]. 2014;13(7):560. Available from: http://dx.doi.org/10.1038/nrd4090-c1

7. Zucker LM, Zucker TF. Fatty, a new mutation in the rat. J Hered. 1961;52(6):275–8.

8. King AJF. The use of animal models in diabetes research. Br J Pharmacol. 2012;166(3):877–94.

9. Charles River Laboratory. ZDF Rat: ZDF-Leprfa/Crl [Internet]. [cited 2016 Nov 14]. Available from: http://www.criver.com/products-services/basic-research/find-a-model/zucker-diabetic-fatty-(zdf)-rat

10. Srinivasan K, Ramarao P. Animal models in type 2 diabetes research: an overview. Indian J Med Res. 2007;125(3):451–72.

11. Mulder GB, Luo S, Gramlich P. The Zucker Diabetic Fatty (ZDF) Rat: Diet Evaluation Study for the Induction of Type 2 Diabetes in Obese Female ZDF Rats [Internet]. Frontiers: A Journal of Women Studies. 2010 [cited 2016 Nov 21]. p. 1–4. Available from: http://www.criver.com/files/pdfs/rms/zdf/rm_rm_r_zdf_diet_eval_tech_sheet.aspx

12. Corsetti JP, Sparks JD, Peterson RG, Smith RL, Sparks CE. Effect of dietary fat on the development of non-insulin dependent diabetes mellitus in obese Zucker diabetic fatty male and female rats. Atherosclerosis [Internet]. 2000;148(2):231–41. Available from: http://www.ncbi.nlm.nih.gov/pubmed/10657558

13. Liu S, Mauvais-Jarvis F. Minireview: Estrogenic protection of β-cell failure in metabolic diseases. Endocrinology. 2010;151(3):859–64.

14. Sowers JR, C A, RF G, DJ B, JR S, E B-C, et al. Diabetes Mellitus and Cardiovascular Disease in Women. Arch Intern Med [Internet]. 1998 Mar 23 [cited 2016 Nov 21];158(6):617. Available from: http://archinte.jamanetwork.com/article.aspx?doi=10.1001/archinte.158.6.617

15. King A, Bowe J. Animal models for diabetes: Understanding the pathogenesis and finding new treatments. Biochem Pharmacol [Internet]. 2016;99:1–10. Available from: http://dx.doi.org/10.1016/j.bcp.2015.08.108

16. Katsuda Y, Ohta T, Miyajima K. Diabetic complications in obese type 2 diabetic rat models. Exp … [Internet]. 2014;63(October 2013):121–32. Available from: http://jlc.jst.go.jp/DN/JALC/10033742310?from=Google

17. Torres TP, Catlin RL, Chan R, Fujimoto Y, Sasaki N, Printz RL, et al. Restoration of hepatic glucokinase expression corrects hepatic glucose flux and normalizes plasma glucose in zucker diabetic fatty rats. Diabetes. 2009;58(1):78–86.

18. Paulsen SJ, Vrang N, Larsen LK, Larsen PJ, Jelsing J. Stereological assessment of pancreatic beta-cell mass development in male Zucker Diabetic Fatty (ZDF) rats: Correlation with pancreatic beta-cell function. J Anat. 2010;217(5):624–30.

19. Hayek A, Woodside W. Correlation between morphology and function in isolated islets of the Zucker rat. Diabetes. 1979;28(6):565–9.

20. Etgen GJ, Oldham B a. Profiling of Zucker diabetic fatty rats in their progression to the overt diabetic state. Metabolism. 2000;49(5):684–8.

21. Vijan S. Type 2 diabetes. Ann Intern Med [Internet]. 2015;162(5):ITC1-16. Available from: http://dx.doi.org/10.7326/AITC201503030

22. Pick A, Clark J, Kubstrup C, Levisetti M, Pugh W, Bonner-Weir S, et al. Role of apoptosis in failure of beta-cell mass compensation for insulin resistance and beta-cell defects in the male Zucker diabetic fatty rat. Diabetes [Internet]. 1998;47(3):358–64. Available from: http://diabetes.diabetesjournals.org/content/47/3/358.long%5Cnpapers3://publication/uuid/F2D2F7AD-C326-444D-B06B-26963F436447

23. Shibata T, Takeuchi S, Yokota S, Kakimoto K, Yonemori F, Wakitani K. Effects of peroxisome proliferator-activated receptor-alpha and -gamma agonist, JTT-501, on diabetic complications in Zucker diabetic fatty rats. Br J Pharmacol [Internet]. 2000;130(3):495–504. Available from: http://www.pubmedcentral.nih.gov/articlerender.fcgi?artid=1572094&tool=pmcentrez&rendertype=abstract

24. Belin De Chantemèle EJ, Vessières E, Guihot AL, Toutain B, Maquignau M, Loufrani L, et al. Type 2 diabetes severely impairs structural and functional adaptation of rat resistance arteries to chronic changes in blood flow. Cardiovasc Res. 2009;81(4):788–96.

25. Leonard BL, Watson RN, Loomes KM, Phillips ARJ, Cooper GJ. Insulin resistance in the Zucker diabetic fatty rat: A metabolic characterisation of obese and lean phenotypes. Acta Diabetol. 2005;42(4):162–70.

26. Howarth FC, Al Kitbi MK, Hameed RS, Adeghate E. Pancreatic peptides in young and elderly Zucker type 2 diabetic fatty rats. J Pancreas. 2011;12(6):567–73.

27. Peterson RG, Shaw WN, Neel M, Little L a, Eichberg J. Zucker Diabetic Fatty Rat as a Model for Non-insulin-dependent Diabetes Mellitus. Ilar News. 1990;32(3):16–9.

28. Clark JB, Palmer CJ, Shaw WN. The diabetic Zucker fatty rat. Proc Soc Exp Biol Med [Internet]. 1983;173(1):68–75. Available from: http://www.ncbi.nlm.nih.gov/entrez/query.fcgi?cmd=Retrieve&db=PubMed&dopt=Citation&list_uids=6344096

29. Tokuyama Y, Sturis J, DePaoli a M, Takeda J, Stoffel M, Tang J, et al. Evolution of beta-cell dysfunction in the male Zucker diabetic fatty rat. Diabetes. 1995;44(12):1447–57.

30. Schmidt RE, Dorsey DA, Beaudet LN, Peterson RG. Analysis of the Zucker Diabetic Fatty (ZDF) type 2 diabetic rat model suggests a neurotrophic role for insulin/IGF-I in diabetic autonomic neuropathy. Am J Pathol [Internet]. 2003;163(1):21–8. Available from: http://www.ncbi.nlm.nih.gov/pubmed/12819007%5Cnhttp://www.ncbi.nlm.nih.gov/pmc/articles/PMC1868158/pdf/3676.pdf

31. Adami ANDREOLLO N, Freitas dos SANTOS E, Rachel ARAÚJO M, Roberto LOPES L, Adami Andreollo N. Rat’s age versus human’s age: what is the relationship? ABCD Arq Bras Cir Dig. 2012;25(1):49–51.

32. Vora JP, Zimsen SM, Houghton DC, Anderson S. Evolution of Metabolic and Renal Changes in the ZDF/Drt-fa Rat Model of Type II Diabetes. J Am Soc Nephrol. 1996;7(1):113–7.

33. Hoshi S, Shu Y, Yoshida F, Inagaki T, Sonoda J, Watanabe T, et al. Podocyte injury promotes progressive nephropathy in zucker diabetic fatty rats. Lab Invest. 2002;82(1):25–35.

34. Chander PN. Nephropathy in Zucker Diabetic Fat Rat Is Associated with Oxidative and Nitrosative Stress: Prevention by Chronic Therapy with a Peroxynitrite Scavenger Ebselen. J Am Soc Nephrol [Internet]. 2004;15(9):2391–403. Available from: http://www.jasn.org/cgi/doi/10.1097/01.ASN.0000135971.88164.2C

35. Danis RP, Yang Y. Microvascular retionopathy in the Zucker diabetic fatty rat. Invest Ophthalmol Vis Sci. 1993;34(7):2367–71.

36. Kim J, Kim CS, Sohn E, Kim H, Jeong IH, Kim JS. KIOM-79 Prevents Lens Epithelial Cell Apoptosis and Lens Opacification in Zucker Diabetic Fatty Rats. Evid Based Complement Altern Med [Internet]. 2011;2011:1–10. Available from: http://www.ncbi.nlm.nih.gov/pubmed/20953387%5Cnhttp://downloads.hindawi.com/journals/ecam/2011/717921.pdf

37. Yang YS, Danis RP, Peterson RG, Dolan PL, Wu YQ. Acarbose partially inhibits microvascular retinopathy in the Zucker Diabetic Fatty rat (ZDF/Gmi-fa). J Ocul Pharmacol Ther [Internet]. 2000;16(5):471–9. Available from: http://www.ncbi.nlm.nih.gov/pubmed/11110039

38. Oltman CL, Coppey LJ, Gellett JS, Davidson EP, Lund DD, Yorek M a. Progression of vascular and neural dysfunction in sciatic nerves of Zucker diabetic fatty and Zucker rats. Am J Physiol Endocrinol Metab. 2005;289(1):E113–22.

39. Shimoshige Y, Ikuma K, Yamamoto T, Takakura S, Kawamura I, Seki J, et al. The effects of zenarestat, an aldose reductase inhibitor, on peripheral neuropathy in Zucker diabetic fatty rats. Metabolism. 2000;49(11):1395–9.

40. Wang B, Chandrasekera P, Pippin J. Leptin- and Leptin Receptor-Deficient Rodent Models: Relevance for Human Type 2 Diabetes. Curr Diabetes Rev [Internet]. 2014;10(2):131–45. Available from: http://www.pubmedcentral.nih.gov/articlerender.fcgi?artid=4082168&tool=pmcentrez&rendertype=abstract%5Cnhttp://www.eurekaselect.com/openurl/content.php?genre=article&issn=1573-3998&volume=10&issue=2&spage=131

41. Doria A, Patti M-E, Kahn CR. The emerging genetic architecture of type 2 diabetes. Cell Metab [Internet]. 2008;8(3):186–200. Available from: http://www.sciencedirect.com/science/article/pii/S1550413108002477

42. Manolio TA, Collins FS, Cox NJ, Goldstein DB, Hindorff LA, Hunter DJ, et al. Finding the missing heritability of complex diseases. Nature [Internet]. 2009;461(7265):747–53. Available from: http://dx.doi.org/10.1038/nature08494

43. Groop L, Pociot F. Genetics of diabetes - Are we missing the genes or the disease? Mol Cell Endocrinol [Internet]. 2014;382(1):726–39. Available from: http://dx.doi.org/10.1016/j.mce.2013.04.002

44. Watanabe RM. The genetics of insulin resistance: Where’s Waldo? Curr Diab Rep. 2010;10(6):476–84.

45. Das SK, Elbein SC. The Genetic Basis of Type 2 Diabetes. Cellscience [Internet]. 2006;2(4):100–31. Available from: http://www.pubmedcentral.nih.gov/articlerender.fcgi?artid=1526773&tool=pmcentrez&rendertype=abstract

46. Rich SS. Mapping genes in diabetes: Genetic epidemiological perspective. Diabetes. 1990;39(11):1315–9.

47. Kahn CR, Vicent D, Doria A. Non-Insulin-Dependent (Type-II) Diabetes Mellitus. Annu Rev Med. 1996;47:509–31.

48. Poulsen P, Ohm Kyvik K, Vaag A, Beck-Nielsen H. Heritability of type II (non-insulin-dependent) diabetes mellitus and abnormal glucose tolerance - A population-based twin study. Diabetologia. 1999;42(2):139–45.

49. Zeggini E, Scott LJ, Saxena R, Voight BF, Marchini JL, Hu T, et al. Meta-analysis of genome-wide association data and large-scale replication identifies additional susceptibility loci for type 2 diabetes. Nat Genet [Internet]. 2008;40(5):638–45. Available from: http://www.pubmedcentral.nih.gov/articlerender.fcgi?artid=2672416&tool=pmcentrez&rendertype=abstract

50. Zeggini E, Weedon MN, Lindgren CM, Frayling TM, Elliott KS, Lango H, et al. Replication of genome-wide association signals in UK samples reveals risk loci for type 2 diabetes. Science (80- ) [Internet]. 2007;316(5829):1336–41. Available from: http://www.pubmedcentral.nih.gov/articlerender.fcgi?artid=3772310&tool=pmcentrez&rendertype=abstract

51. Saxena R, Voight BF, Lyssenko V, Burtt NP, Bakker PIW de, Chen H, et al. Genome-Wide Association Analysis Identifies Loci for Type 2 Diabetes and Triglyceride Levels. Science (80- ). 2007;316(5829):1331–6.

52. PubMed. TCF7L2 transcription factor 7 like 2 [Rattus norvegicus (Norway rat)] [Internet]. [cited 2016 Nov 21]. Available from: https://www.ncbi.nlm.nih.gov/gene/679869

53. PubMed. Pparg peroxisome proliferator-activated receptor gamma [Rattus norvegicus (Norway rat)] [Internet]. [cited 2016 Nov 21]. Available from: https://www.ncbi.nlm.nih.gov/gene/25664

54. PubMed. Kcnj11 potassium voltage-gated channel subfamily J member 11 [Rattus norvegicus (Norway rat)] [Internet]. [cited 2016 Nov 21]. Available from: https://www.ncbi.nlm.nih.gov/gene/83535

55. PubMed. TCF7L2 transcription factor 7 like 2 [Homo sapiens (human)] [Internet]. [cited 2016 Nov 21]. Available from: https://www.ncbi.nlm.nih.gov/gene/6934

56. Prunier C. HBAHPH. Wnt signaling: Physiology and pathology. Growth Factors. 2004;22(3):141–50.

57. Yi F, Brubaker PL, Jin T. TCF-4 mediates cell type-specific regulation of proglucagon gene expression by β-catenin and glycogen synthase kinase-3β*. J Biol Chem. 2005;280(2):1457–64.

58. Boj SF, Van Es JH, Huch M, Li VSW, José A, Hatzis P, et al. Diabetes risk gene and wnt effector Tcf7l2/TCF4 controls hepatic response to perinatal and adult metabolic demand. Cell. 2012;151(7):1595–607.

59. Tong Y, Lin Y, Zhang YY, Yang J, Zhang YY, Liu H, et al. Association between TCF7L2gene polymorphisms and susceptibility to Type 2 Diabetes Mellitus: a large Human Genome Epidemiology (HuGE) review and meta-analysis. BMC Med Genet [Internet]. 2009;10(1):15. Available from: http://dx.doi.org/10.1186/1471-2350-10-15

60. Grant SF a, Thorleifsson G, Reynisdottir I, Benediktsson R, Manolescu A, Sainz J, et al. Variant of transcription factor 7-like 2 (TCF7L2) gene confers risk of type 2 diabetes. Nat Genet. 2006;38(3):320–3.

61. Fehmann H-C, Göke R, Göke B. Cell and Molecular Biology of the Incretin Hormones Glucagon-Like Peptide-I and Glucose-Dependent Insulin Releasing Polypeptide. Endocr Rev [Internet]. 1995;16(3):390. Available from: ]

62. Lyssenko V, Lupi R, Marchetti P, Del Guerra S, Orho-Melander M, Almgren P, et al. Mechanisms by which common variants in the TCF7L2 gene increase risk of type 2 diabetes. JClinInvest. 2007;117(8):2155–63.

63. Cauchi S, Meyre D, Dina C, Choquet H, Samson C, Gallina S, et al. Transcription factor TCF7L2 genetic study in the French population: expression in human beta-cells and adipose tissue and strong association with type 2 diabetes. Diabetes. 2006;55(October):2903–8.

64. Grant SFA. Understanding the elusive mechanism of action of TCF7L2 in metabolism. Diabetes. 2012;61(11):2657–8.

65. Kaminska D, Kuulasmaa T, Venesmaa S, Käkelä P, Vaittinen M, Pulkkinen L, et al. Adipose tissue TCF7L2 Splicing is regulated by weight loss and associates with glucose and fatty acid metabolism. Diabetes. 2012;61(11):2807–13.

66. McCarthy MI, Rorsman P, Gloyn AL. TCF7L2 and diabetes: A tale of two tissues, and of two species. Cell Metab [Internet]. 2013;17(2):157–9. Available from: http://dx.doi.org/10.1016/j.cmet.2013.01.011

67. PubMed. KCNJ11 potassium voltage-gated channel subfamily J member 11 [Homo sapiens (human)] [Internet]. [cited 2016 Nov 21]. Available from: https://www.ncbi.nlm.nih.gov/gene/3767

68. Hani EH, Boutin P, Durand E, Inoue H, Permutt MA, Velho G, et al. Missense mutations in the pancreatic islet beta cell inwardly rectifying K+ channel gene (KIR6.2/BIR): A meta-analysis suggests a role in the polygenic basis of Type II diabetes mellitus in Caucasians. Diabetologia. 1998;41(12):1511–5.

69. Koster JC, Marshall BA, Ensor N, Corbett JA, Nichols CG. Targeted overactivity of beta cell K(ATP) channels induces profound neonatal diabetes. Cell [Internet]. 2000;100(6):645–54. Available from: http://www.ncbi.nlm.nih.gov/pubmed/10761930

70. PubMed. PPARG peroxisome proliferator activated receptor gamma [Homo sapiens (human)] [Internet]. [cited 2016 Nov 21]. Available from: https://www.ncbi.nlm.nih.gov/gene/5468

71. Tontonoz P, Hu E, Spiegelman BM. Stimulation of adipogenesis in fibroblasts by PPARγ2, a lipid-activated transcription factor. Cell. 1994;79(7):1147–56.

72. Tontonoz P, Hu E, Graves RA, Budavari AI, Spiegelman BM. mPPARγ2: tissue specific regulator of an adipocyte enhancer. Genes Dev. 1994;8(10):1224–34.

73. Tontonoz P, Hu E, Spiegelman BM. Regulation of adipocyte gene expression and differentiation by peroxisome proliferator activated receptor γ. Curr Opin Genet Dev [Internet]. 1995;5(5):571–6. Available from: http://www.sciencedirect.com/science/article/pii/0959437X95800255

74. Werman A, Hollenberg A, Solanes G, Bjorbaek C, Vidal-Puig AJ, Flier JS. Ligand-independent Activation Domain in the N Terminus of Peroxisome Proliferator-activated Receptor (PPARγ): Differential Activity Of PPPARγ1 And -2 Isoforms And Influence Of Insulin. J Biol Chem [Internet]. 1997;272(32):20230–5. Available from: http://www.jbc.org/content/272/32/20230

75. Fajas L, Auboeuf D, Raspé E, Schoonjans K, Lefebvre AM, Saladin R, et al. The organization, promoter analysis, and expression of the human PPARgamma gene. J Biol Chem [Internet]. 1997;272(30):18779–89. Available from: http://www.ncbi.nlm.nih.gov/pubmed/9228052

76. Florez JC, Jablonski KA, Bayley N, Pollin TI, Bakker PIW de, Shuldiner AR, et al. TCF7L2 Polymorphisms and Progression to Diabetes in the Diabetes Prevention Program. N Engl J Med. 2006;355(3):241–50.

77. Schwanstecher C, Meyer U, Schwanstecher M. KIR6.2 Polymorphism Predisposes to T2D by Inducing Overactivity of β-Cell ATP-Sensitive K+ Channels. Diabetes. 2002;51(3):875–9.

78. Nielsen ED, Hansen L, Carstensen B, Echwald SM, Drivsholm T, Glümer C, et al. The E23K Varian of Kir6.2 Associates With Impaired Post-OGTT Serum Insulin Response and Increased Risk of Type 2 Diabetes. Diabetes. 2003;52(2):573–7.

79. Florez JC, Sjögren M, Burtt N, Orho-Melander M, Schayer S, Sun M, et al. Association Testing in 9,000 People Fails to Confirm the Association of the Insulin Receptor Substrate-1 G972R Polymorphism With Type 2 Diabetes. Diabetes. 2004;53(12):3313–8.

80. Scott LJ, Mohlke KL, Bonnycastle LL, Willer CJ, Li Y, Duren WL, et al. A genome-wide association study of type 2 diabetes in Finns detects multiple susceptibility variants. Science (80- ) [Internet]. 2007;316(June):1341–5. Available from: http://www.pubmedcentral.nih.gov/articlerender.fcgi?artid=3214617&tool=pmcentrez&rendertype=abstract

81. Parton LE, Ye CP, Coppari R, Enriori PJ, Choi B, Zhang C-Y, et al. Glucose sensing by POMC neurons regulates glucose homeostasis and is impaired in obesity. Nature. 2007;449(7159):228–32.

82. Deeb SS, Fajas L, Nemoto M, Pihlajamaki J, Mykkanen L, Kuusisto J, et al. A Pro12Ala substitution in PPAR gamma 2 associated with decreased receptor activity, lower body mass index and improved insulin sensitivity. Nat Genet. 1998;20(3):284–7.

83. Beamer BA, Yen CJ, Andersen RE, Muller D, Elahi D, Cheskin LJ, et al. Association of the Pro12Ala variant in the peroxisome proliferator- activated receptor-??2 gene with obesity in two Caucasian populations. Diabetes. 1998;47(11):1806–8.

84. Altshuler D, Hirschhorn JN, Klannemark M, Lindgren CM, Vohl MC, Nemesh J, et al. The common PPARgamma Pro12Ala polymorphism is associated with decreased risk of type 2 diabetes. Nat Genet [Internet]. 2000;26(september):76–80. Available from: http://www.ncbi.nlm.nih.gov/pubmed/10973253

85. Ruiz-Narvaez EA, Kraft P, Campos H. Ala 2 variant of the peroxisome proliferator-activated receptor-gamma gene (PPARG) is associated with higher polyunsaturated fat in adipose tissue and attenuates the protective effect of polyunsaturated fat intake on the risk of myocardial infarction. Am J Clin Nutr. 2007;86(4):1238–42.

86. Li Y, Zhu J, Ding JQ. Association of the PPARγ2 Pro12Ala polymorphism with increased risk of cardiovascular diseases. Genet Mol Res. 2015;14(4):18662–74.

87. Taneera J, Lang S, Sharma A, Fadista J, Zhou Y, Ahlqvist E, et al. A Systems Genetics Approach Identifies Genes and Pathways for Type 2 Diabetes in Human Islets. Cell Metab [Internet]. 2012 [cited 2017 Apr 20];16(1):122–34. Available from: http://www.sciencedirect.com/science/article/pii/S1550413112002434

88. Shu L, Sauter NS, Schulthess FT, Matveyenko A V. Transcription Factor 7-Like 2 Regulates B-cell Survival and Function in Human Pancreatic Islets. Diabetes. 2008;57(March):645–53.

89. Parton LE, McMillen PJ, Shen Y, Docherty E, Sharpe E, Diraison F, et al. Limited role for SREBP-1c in defective glucose-induced insulin secretion from Zucker diabetic fatty rat islets: a functional and gene profiling analysis. Am J Physiol Endocrinol Metab [Internet]. 2006;291(5):E982-94. Available from: http://www.ncbi.nlm.nih.gov/pubmed/16772326

90. Tokuyama Y, Fan Z, Furuta H, Makielski JC, Polonsky KS, Bell GI, et al. Rat inwardly rectifying potassium channel Kir6.2: cloning electrophysiological characterization, and decreased expression in pancreatic islets of male Zucker diabetic fatty rats. Biochem Biophys Res Commun [Internet]. 1996;220(3):532–8. Available from: file://i/Chercheurs/Brochiero_Emmanuelle/Brochiero/Ref Manager/100/175.pdf

91. Gyte A, Pritchard LE, Jones HB, Brennand JC, White A. Reduced expression of the KATP Channel Subunit, Kir6.2, is associated with decreased expression of neuropeptide Y and agouti-related protein in the hypothalami of zucker diabetic fatty rats. J Neuroendocrinol. 2007;19(12):941–51.

92. Rosengren AH, Braun M, Mahdi T, Andersson SA, Travers ME, Shigeto M, et al. Reduced insulin exocytosis in human pancreatic β-cells with gene variants linked to type 2 diabetes. Diabetes. 2012;61(7):1726–33.

93. Parikh H, Lyssenko V, Groop LC. Prioritizing genes for follow-up from genome wide association studies using information on gene expression in tissues relevant for type 2 diabetes mellitus. BMC Med Genomics [Internet]. 2009;2(1):72. Available from: http://dx.doi.org/10.1186/1755-8794-2-72

94. Stratton IM, Adler AI, Neil HAW, Matthews DR, Manley SE, Cull CA, et al. Association of glycaemia with macrovascular and microvascular complications of type 2 diabetes (UKPDS 35): prospective observational study. Br Med J [Internet]. 2000;321(7258):405–12. Available from: http://www.bmj.com/content/321/7258/405?linkType=FULL&ck=nck&resid=321/7258/405&journalCode=bmj

95. UKPDS. Intensive blood-glucose control with sulphonylureas or insulin compared with conventional treatment and risk of complications in patients with type 2 diabetes (UKPDS 33). Lancet. 1998;352(9131):837–53.

96. Inzucchi SE, Bergenstal RM, Buse JB, Diamant M, Ferrannini E, Nauck M, et al. Management of hyperglycaemia in type 2 diabetes, 2015: a patient-centred approach. Update to a Position Statement of the American Diabetes Association and the European Association for the Study of Diabetes. Diabetologia. 2015;58(3):429–42.

97. American Diabetes Association. Standards of Medical care in diabetes - 2017. J Clin Appl Res Educ. 2017;40(January):1–142.

98. Ford ES, Zhao G, Li C. Pre-Diabetes and the Risk for Cardiovascular Disease. A Systematic Review of the Evidence. J Am Coll Cardiol [Internet]. 2010;55(13):1310–7. Available from: http://dx.doi.org/10.1016/j.jacc.2009.10.060

99. Ley SH, Korat AVA, Sun Q, Tobias DK, Zhang C, Qi L, et al. Contribution of the nurses’ health studies to uncovering risk factors for type 2 diabetes: diet, lifestyle, biomarkers, and genetics. Am J Public Health. 2016;106(9):1624–30.

100. Frank B. Hu, Joann E Manson, Meir J Stampfer, Graham Colditz, Simin Liu CGS. Diet, lifestyle, and the risk of type 2 diabetes melitus in women. N Engl J Med. 2001;345(11):790–7.

101. Phillips MS, Liu Q, Hammond HA, Dugan V, Hey PJ, Caskey CJ, et al. Leptin receptor missense mutation in the fatty Zucker rat. Nat Genet [Internet]. 1996;13(1):18–9. Available from: http://www.ncbi.nlm.nih.gov/pubmed/8673096

102. Griffen SC, Wang J, German MS. A genetic defect in beta-cell gene expression segregates independently from the fa locus in the ZDF rat.[In Process Citation]. Diabetes [Internet]. 2001;50(1):63–8. Available from: http://www.ncbi.nlm.nih.gov/htbin-post/Entrez/query?db=m&form=6&dopt=r&uid=0011147796

103. Sone M, Osamura RY. Leptin and the pituitary. Pituitary. 2001;4(1–2):15–23.

104. Paracchini V, Pedotti P, Taioli E. Genetics of leptin and obesity: A HuGE review. Am J Epidemiol. 2005;162(2):101–14.

105. Murugesan D, Arunachalam T, Ramamurthy V, Subramanian S. Association of polymorphisms in leptin receptor gene with obesity and type 2 diabetes in the local population of Coimbatore. Indian J Hum Genet. 2010;16(2):72–7.

106. Wang Z, Zhuo Q, Fu P, Piao J, Tian Y, Xu J, et al. Are the associations of plasma leptin and adiponectin with type 2 diabetes independent of obesity in older Chinese adults? Diabetes Metab Res Rev. 2010;26(2):109–14.

107. Sempoux C, Guiot Y, Dubois D, Moulin P, Rahier J. Human type 2 diabetes: morphological evidence for abnormal beta-cell function. Diabetes. 2001;50(Suppl 1):S172–7.

108. Bonner-Weir S, O’Brien TD. Islets in type 2 diabetes: In honor of Dr. Robert C. Turner. Diabetes. 2008;57(11):2899–904.

109. Tomita T. Islet amyloid polypeptide in pancreatic islets from type 2 diabetic subjects. Islets. 2012;4(3):223–32.

110. Yagihashi S. Advances in pathology of diabetes from pancreatic islets to neuropathy - a tribute to Paul Langerhans. Pathol Int [Internet]. 2015 Apr 1 [cited 2017 Jun 13];65(4):157–69. Available from: http://doi.wiley.com/10.1111/pin.12263

111. Finegood DT, Mcarthur MD, Kojwang D, Thomas MJ, Topp BG, Leonard T, et al. Beta-cell mass Dynamics in Zucker Diabetic Fatty Rats. Rosiglitazone Prevents the Rise in Net Cell Death. Diabetes. 2001;50(6):1021–9.

112. Kakimoto T, Kimata H, Iwasaki S, Fukunari A, Utsumi H. Automated recognition and quantification of pancreatic islets in Zucker diabetic fatty rats treated with exendin-4. J Endocrinol. 2013;216(1):13–20.

113. Jones HB, Bigley AL, Pemberton J, Randall KJ. Quantitative histopathological assessment of retardation of islets of langerhans degeneration in rosiglitazone-dosed obese ZDF rats using combined insulin and collagens (I and III) immunohistochemistry with automated image analysis and statistical modelin. Toxicol Pathol [Internet]. 2013;41(3):425–44. Available from: http://www.ncbi.nlm.nih.gov/pubmed/23047688

114. Janssen SW, Hermus AR, Lange WP, Knijnenburg Q, van der Laak JA, Sweep CG, et al. Progressive histopathological changes in pancreatic islets of Zucker Diabetic Fatty rats. Exp Clin Endocrinol Diabetes [Internet]. 2001;109(5):273–82. Available from: http://www.ncbi.nlm.nih.gov/pubmed/11507651

115. Inoue K, Hiramatsu S, Hisatomi A, Umeda F, Nawata H. Hypersecretion of amylin from the perfused pancreas of genetically obese (fa/fa) rats and its alteration with aging. Metabolism [Internet]. 1993 May [cited 2017 Jun 13];42(5):654–8. Available from: http://linkinghub.elsevier.com/retrieve/pii/002604959390227F

116. Nistala R, Raja A, Pulakat L. mTORC1 inhibitors rapamycin and metformin affect cardiovascular markers differentially in ZDF rats. Can J Physiol Pharmacol. 2017;95(3):281–7.

117. Tsumura Y, Tsushima Y, Tamura A, Hasebe M, Kanou M, Kato H, et al. TMG-123, a novel glucokinase activator, exerts durable effects on hyperglycemia without increasing triglyceride in diabetic animal models. PLoS One [Internet]. 2017;12(2):1–16. Available from: http://dx.plos.org/10.1371/journal.pone.0172252%0Ahttp://www.ncbi.nlm.nih.gov/pubmed/28207836

118. Ito R, Tsujihata Y, Matsuda-Nagasumi K, Mori I, Negoro N, Takeuchi K. TAK-875, a GPR40/FFAR1 agonist, in combination with metformin prevents progression of diabetes and β-cell dysfunction in Zucker diabetic fatty rats. Br J Pharmacol. 2013;170(3):568–80.

119. Schmid PM, Resch M, Schach C, Birner C, Riegger G a, Luchner A, et al. Antidiabetic treatment restores adiponectin serum levels and APPL1 expression, but does not improve adiponectin-induced vasodilation and endothelial dysfunction in Zucker diabetic fatty rats. Cardiovasc Diabetol [Internet]. 2013;12(1):46. Available from: http://www.pubmedcentral.nih.gov/articlerender.fcgi?artid=3606629&tool=pmcentrez&rendertype=abstract

120. Han SJ, Choi SE, Kang Y, Jung JG, Yi SA, Kim HJ, et al. Effect of sitagliptin plus metformin on β-cell function, islet integrity and islet gene expression in Zucker diabetic fatty rats. Diabetes Res Clin Pract [Internet]. 2011;92(2):213–22. Available from: http://dx.doi.org/10.1016/j.diabres.2011.01.016

121. Takiyama Y, Harumi T, Watanabe J, Fujita Y, Honjo J, Shimizu N, et al. Tubular injury in a rat model of type 2 diabetes is prevented by metformin: A possible role of HIF-1?? expression and oxygen metabolism. Diabetes. 2011;60(3):981–92.

122. Hwang IK, Kim IY, Joo EJ, Shin JH, Choi JW, Won MH, et al. Metformin normalizes type 2 diabetes-induced decrease in cell proliferation and neuroblast differentiation in the rat dentate gyrus. Neurochem Res. 2010;35(4):645–50.

123. Forcheron F, Basset A, Carmine P Del, Beylot M. Lipase maturation factor 1: Its expression in Zucker diabetic rats, and effects of metformin and fenofibrate. Diabetes Metab. 2009;35(35):452–7.

124. Shoghi KI, Finck BN, Schechtman KB, Herrero TSP, Gropler RJ, Welch MJ. In vivo metabolic phenotyping of myocardial substrate metabolism in rodents: Differential efficacy of metformin and rosiglitazone monotherapy. Circ Cardiovasc Imaging. 2009;2(5):373–81.

125. Forcheron F, Abdallah P, Basset A, del Carmine P, Haffar G, Beylot M. Nonalcoholic hepatic steatosis in Zucker diabetic rats: spontaneous evolution and effects of metformin and fenofibrate. Obesity (Silver Spring) [Internet]. 2009;17(7):1381–9. Available from: http://www.ncbi.nlm.nih.gov/pubmed/19553925

126. Metais C, Forcheron F, Abdallah P, Basset A, Del Carmine P, Bricca G, et al. Adiponectin receptors: expression in Zucker diabetic rats and effects of fenofibrate and metformin. Metabolism. 2008;57(7):946–53.

127. Smith AC, Mullen KL, Junkin KA, Nickerson J, Chabowski A, Bonen A, et al. Metformin and exercise reduce muscle FAT/CD36 and lipid accumulation and blunt the progression of high-fat diet-induced hyperglycemia. Am J Physiol Endocrinol Metab [Internet]. 2007;293(1):E172-81. Available from: http://www.ncbi.nlm.nih.gov/pubmed/17374701

128. Othman EM, Oli RG, Arias-Loza PA, Kreissl MC, Stopper H. Metformin Protects Kidney Cells From Insulin-Mediated Genotoxicity In Vitro and in Male Zucker Diabetic Fatty Rats. Endocrinology. 2016;157(2):548–59.

129. Sreenan S, Sturis J, Pugh W, Burant CF, Polonsky KS. Prevention of hyperglycemia in the Zucker diabetic fatty rat by treatment with metformin or troglitazone. Am J Physiol. 1996;271:E742–7.

130. Forcheron F, Basset A, Abdallah P, Del Carmine P, Gadot N, Beylot M. Diabetic cardiomyopathy: effects of fenofibrate and metformin in an experimental model--the Zucker diabetic rat. Cardiovasc Diabetol. 2009;8(16):1–13.

131. Dong Y, Chen Y-T, Yang Y-X, Shou D, Li C-Y. Urinary Metabolomic Profiling in Zucker Diabetic Fatty Rats with Type 2 Diabetes Mellitus Treated with Glimepiride, Metformin, and Their Combination. Molecules [Internet]. 2016;21(12):1446. Available from: http://www.mdpi.com/1420-3049/21/11/1446

132. Chen W-P, Wang Y-D, Ma Y, Zhang Z-Y, Hu L-Y, Lin J-L, et al. Danhong Huayu Koufuye combined with metformin attenuated diabetic retinopathy in Zucker diabetic fatty. Int J Opthalmology. 2015;8(6):1094–100.

133. Forest T, Holder D, Smith A, Cunningham C, Yao X, Dey M, et al. Characterization of the exocrine pancreas in the male Zucker diabetic fatty rat model of type 2 diabetes mellitus following 3 months of treatment with sitagliptin. Endocrinology. 2014;155(3):783–92.

134. Reimer RA, Grover GJ, Koetzner L, Gahler RJ, Lyon MR, Wood S. Combining sitagliptin/metformin with a functional fiber delays diabetes progression in zucker rats. J Endocrinol. 2014;220(3):361–73.

135. Wessels B, Ciapaite J, Van Den Broek NMA, Nicolay K, Prompers JJ. Metformin impairs mitochondrial function in skeletal muscle of both lean and diabetic rats in a Dose-dependent manner. PLoS One. 2014;9(6):1–10.

136. Konda VR, Desai A, Darland G, Grayson N, Bland JS. KDT501, a derivative from hops, normalizes glucose metabolism and body weight in rodent models of diabetes. PLoS One. 2014;9(1):1–11.

137. Kim YN, Kim S, Kim IY, Shin JH, Cho S, Yi SS, et al. Transcriptomic Analysis of Insulin-Sensitive Tissues from Anti-Diabetic Drug Treated ZDF Rats, a T2DM Animal Model. PLoS One. 2013;8(7):1–9.

138. Shang Q, Liu MK, Saumoy M, Holst JJ, Salen G, Xu G. The combination of colesevelam with sitagliptin enhances glycemic control in diabetic ZDF rat model. AJP Gastrointest Liver Physiol [Internet]. 2012;302(8):G815–23. Available from: http://www.ncbi.nlm.nih.gov/pubmed/22281473%5Cnhttp://ajpgi.physiology.org/content/ajpgi/302/8/G815.full.pdf

139. Oguma T, Nakayama K, Kuriyama C, Matsushita Y, Yoshida K, Hikida K, et al. Intestinal Sodium Glucose Cotransporter 1 Inhibition Enhances Glucagon-Like Peptide-1 Secretion in Normal and Diabetic Rodents. J Pharmacol Exp Ther [Internet]. 2015;354(3):279–89. Available from: http://jpet.aspetjournals.org/content/354/3/279.long

140. Takai S, Sakonjo H, Jin D. Significance of vascular dipeptidyl peptidase-4 inhibition on vascular protection in zucker diabetic Fatty rats. J Pharmacol Sci [Internet]. 2014;125(4):386–93. Available from: http://www.ncbi.nlm.nih.gov/pubmed/25030743

141. Marques C, Mega C, Gonçalves A, Rodrigues-Santos P, Teixeira-Lemos E, Teixeira F, et al. Sitagliptin prevents inflammation and apoptotic cell death in the kidney of type 2 diabetic animals. Mediators Inflamm. 2014;2014:1–15.

142. Mega C, Vala H, Rodrigues-Santos P, Oliveira J, Teixeira F, Fernandes R, et al. Sitagliptin prevents aggravation of endocrine and exocrine pancreatic damage in the Zucker Diabetic Fatty rat - focus on amelioration of metabolic profile and tissue cytoprotective properties. Diabetol Metab Syndr. 2014;6:42.

143. Mega C, Teixeira De Lemos E, Vala H, Fernandes R, Oliveira J, Mascarenhas-Melo F, et al. Diabetic nephropathy amelioration by a low-dose sitagliptin in an animal model of type 2 diabetes (Zucker diabetic fatty rat). Exp Diabetes Res. 2011;2011:1–12.

144. Gonçalves A, Leal E, Paiva A, Teixeira Lemos E, Teixeira F, Ribeiro CF, et al. Protective effects of the dipeptidyl peptidase IV inhibitor sitagliptin in the blood-retinal barrier in a type 2 diabetes animal model. Diabetes, Obes Metab. 2012;14(5):454–63.

145. Zhou W, Liu Z, Yao J, Chi F, Dong K, Yue X, et al. The Effects of Exenatide Microsphere on Serum BGP and ALP Levels in ZDF Rats after Implantation. Clin Implant Dent Relat Res. 2013;(Dm):765–70.

146. Vrang N, Jelsing J, Simonsen L, Jensen AE, Thorup I, Søeborg H, et al. The effects of 13 wk of liraglutide treatment on endocrine and exocrine pancreas in male and female ZDF rats: a quantitative and qualitative analysis revealing no evidence of drug-induced pancreatitis. Am J Physiol Endocrinol Metab [Internet]. 2012;303(2):E253-64. Available from: http://www.ncbi.nlm.nih.gov/pubmed/22589391

147. Kwak HH, Shim WS, Hwang S, Son MK, Kim YJ, Kim TH, et al. Pharmacokinetics and efficacy of a biweekly dosage formulation of exenatide in zucker diabetic fatty (ZDF) rats. Pharm Res. 2009;26(11):2504–12.

148. Kwak HH, Shim WS, Son MK, Kim YJ, Kim TH, Youn HJ, et al. Efficacy of a new sustained-release microsphere formulation of exenatide, DA-3091, in Zucker diabetic fatty (ZDF) rats. Eur J Pharm Sci [Internet]. 2010;40(2):103–9. Available from: http://dx.doi.org/10.1016/j.ejps.2010.03.006

149. Gedulin BR, Smith P, Prickett KS, Tryon M, Barnhill S, Reynolds J, et al. Dose-response for glycaemic and metabolic changes 28 days after single injection of long-acting release exenatide in diabetic fatty Zucker rats. Diabetologia. 2005;48(7):1380–5.

150. Castillo GM, Reichstetter S, Bolotin EM. Extending residence time and stability of peptides by Protected Graft Copolymer (PGC) excipient: GLP-1 example. Pharm Res. 2012;29(1):306–18.

151. Tatarkiewicz K, Belanger P, Gu G, Parkes D, Roy D. No evidence of drug-induced pancreatitis in rats treated with exenatide for 13 weeks. Diabetes Obes Metab. 2013;15(5):417–26.

152. Wang X, Liu X, Zhan Y, Lavallie ER, Diblasio-Smith L, Collins-Racie L, et al. Pharmacogenomic, physiological, and biochemical investigations on safety and efficacy biomarkers associated with the peroxisome proliferator-activated receptor-gamma activator rosiglitazone in rodents: a translational medicine investigation. J Pharmacol Exp Ther [Internet]. 2010;334(3):820–9. Available from: http://www.ncbi.nlm.nih.gov/pubmed/20519551

153. Cosson E, Cohen-Boulakia F, Tarhzaoui K, Dabiré H, Léger G, Charnaux N, et al. Capillary endothelial but not lymphatic function is restored under rosiglitazone in Zucker Diabetic Fatty rats. Microvasc Res [Internet]. 2009;77(2):220–5. Available from: http://dx.doi.org/10.1016/j.mvr.2008.11.005

154. Mansour M, Coleman E, Dennis J, Akingbemi B, Schwartz D, Braden T, et al. Activation of PPAR by rosiglitazone does not negatively impact male sex steroid mormones in diabetic rats. PPAR Res. 2009;2009:1–8.

155. Iqbal MJ, Higginbotham A, Chickris N, Bollaert M, Rockway S, Banz WJ. A combination of CLA-DAG oil modifies the diabetic phenotype in male zucker diabetic fatty rats. Horm Metab Res. 2008;40(4):262–8.

156. Oltman CL, Davidson EP, Coppey LJ, Kleinschmidt TL, Lund DD, Adebara ET, et al. Vascular and neural dysfunction in Zucker diabetic fatty rats: A difficult condition to reverse. Diabetes, Obes Metab. 2008;10(1):64–74.

157. Banz WJ, Iqbal MJ, Bollaert M, Chickris N, James B, Higginbotham DA, et al. Ginseng modifies the diabetic phenotype and genes associated with diabetes in the male ZDF rat. Phytomedicine. 2007;14(10):681–9.

158. Gonzalez IC, Lamar J, Iradier F, Xu Y, Winneroski LL, York J, et al. Design and synthesis of a novel class of dual PPARgamma/delta agonists. Bioorg Med Chem Lett [Internet]. 2007;17(4):1052–5. Available from: http://www.ncbi.nlm.nih.gov/pubmed/17129725

159. Nadeau KJ, Ehlers LB, Aguirre LE, Reusch JEB, Draznin B. Discordance between intramuscular triglyceride and insulin sensitivity in skeletal muscle of Zucker diabetic rats after treatment with fenofibrate and rosiglitazone. Diabetes, Obes Metab. 2007;9(5):714–23.

160. Shi Q, Canada EJ, Xu Y, Warshawsky AM, Etgen GJ, Broderick CL, et al. Design and synthesis of novel and potent amide linked PPARgamma/delta dual agonists. Bioorg Med Chem Lett [Internet]. 2007;17(24):6744–9. Available from: http://www.ncbi.nlm.nih.gov/pubmed/18029178

161. Banz WWJ, Davis J, Steinle JJ, Adler S, Oitker J, Winters TA, et al. (+)-Z-Bisdehydrodoisynolic Acid Ameliorates Obesity and the Metabolic Syndrome in Female ZDF Rats. Obesity [Internet]. 2005;13(11):1915–24. Available from: http://www.nature.com/oby/journal/v13/n11/abs/oby2005236a.html%5Cnhttp://doi.wiley.com/10.1038/oby.2005.236%5Cnhttp://www.ncbi.nlm.nih.gov/pubmed/16339123

162. Johns DG, Ao Z, Eybye M, Olzinski A, Costell M, Gruver S, et al. Rosiglitazone Protects against Ischemia / Reperfusion-Induced Leukocyte Adhesion in the Zucker Diabetic Fatty Rat. Pharmacology. 2005;315(3):1020–7.

163. Reifel-Miller A, Otto K, Hawkins E, Barr R, Bensch WR, Bull C, et al. A peroxisome proliferator-activated receptor alpha/gamma dual agonist with a unique in vitro profile and potent glucose and lipid effects in rodent models of type 2 diabetes and dyslipidemia. Mol Endocrinol [Internet]. 2005;19(6):1593–605. Available from: http://www.ncbi.nlm.nih.gov/pubmed/15831517

164. Winter CL, Lange JS, Davis MG, Gerwe GS, Downs TR, Peters KG, et al. A Nonspecific Phosphotyrosine Phosphatase Inhibitor , Bis (maltolato) oxovanadium (IV), Improves Glucose Tolerance and Prevents Diabetes in Zucker Diabetic Fatty Rats. Exp Biol Med. 2005;230(3):207–16.

165. Yue T, Bao W, Gu J, Cui J, Tao L, Ma X, et al. Rosiglitazone treatment in Zucker diabetic Fatty rats is associated with ameliorated cardiac insulin resistance and protection from ischemia/reperfusion-induced myocardial injury. Diabetes [Internet]. 2005;54(2):554–62. Available from: http://www.ncbi.nlm.nih.gov/pubmed/15677515

166. Banz WJ, Davis J, Peterson R, Iqbal MJ. Gene expression and adiposity are modified by soy protein in male Zucker diabetic fatty rats. Obes Res [Internet]. 2004;12(12):1907–13. Available from: http://www.ncbi.nlm.nih.gov/pubmed/15687389

167. Kuhlmann J, Neumann-Haefelin C, Belz U, Kalisch J, Juretschke HP, Stein M, et al. Intramyocellular lipid and insulin resistance: A longitudinal in vivo 1H-spectroscopic study in Zucker Diabetic Fatty rats. Diabetes. 2003;52(1):138–44.

168. Yuen VG, Bhanot S, Battell ML, Orvig C, McNeill JH. Chronic glucose-lowering effects of rosiglitazone and bis(ethylmaltolato)oxovanadium(IV) in ZDF rats. Can J Physiol Pharmacol [Internet]. 2003;81(11):1049–55. Available from: http://eutils.ncbi.nlm.nih.gov/entrez/eutils/elink.fcgi?dbfrom=pubmed&amp;id=14719040&amp;retmode=ref&amp;cmd=prlinks

169. Kim YY, Kang KM, Chung SH. Long-term administration of Sopungsungi-won (SP) prevents diabetic nephropathy in Zucker diabetic fatty rats. Arch Pharm Res [Internet]. 2002;25(6):917–22. Available from: http://www.ncbi.nlm.nih.gov/pubmed/12510848

170. Kim YY, Kang HJ, Ko SK, Chung SH. Sopungsungi-won (SP) prevents the onset of hyperglycemia and hyperlipidemia in Zucker diabetic fatty rats. Arch Pharm Res [Internet]. 2002;25(6):923–31. Available from: http://link.springer.com/content/pdf/10.1007/BF02977015.pdf

171. Singh Ahuja H, Liu SHA, Crombie DL, Boehm M, Leibowitz MD, Heyman R a, et al. Differential effects of rexinoids and thiazolidinediones on metabolic gene expression in diabetic rodents. Mol Pharmacol [Internet]. 2001;59(4):765–73. Available from: http://www.ncbi.nlm.nih.gov/pubmed/11259621

172. Dana SL, Hoener PA, Bilakovics JM, Crombie DL, Ogilvie KM, Kauffman RF, et al. Peroxisome proliferator-activated receptor subtype-specific regulation of hepatic and peripheral gene expression in the Zucker diabetic fatty rat. Metabolism. 2001;50(8):963–71.

173. Way JM, Görgün CZ, Tong Q, Uysal KT, Brown KK, Harrington WW, et al. Adipose Tissue Resistin Expression is Severely Suppressed in Obesity and Stimulated by Peroxisome Proliferator-activated Receptor ?? Agonists. J Biol Chem. 2001;276(28):25651–3.

174. Cai XJ, Lister CA, Buckingham RE, Pickavance L, Wilding J, Arch JRS, et al. Down-regulation of orexin gene expression by severe obesity in the rats: Studies in Zucker fatty and Zucker diabetic fatty rats and effects of rosiglitazone. Mol Brain Res. 2000;77(1):131–7.

175. Maier VH, Melvin DR, Lister CA, Chapman H, Gould GW, Murphy GJ. v- and t-SNARE protein expression in models of insulin resistance: Normalization of glycemia by rosiglitazone treatment corrects overexpression of cellubrevin, vesicle-associated membrane protein-2, and syntaxin 4 in skeletal muscle of Zucker diabetic fat. Diabetes. 2000;49(4):618–25.

176. Smith SA, Lister CA, Toseland CDN, Buckingham RE. Rosiglitazone prevents the onset of hyperglycaemia and proteinuria in the Zucker diabetic fatty rat. Diabetes, Obes Metab. 2000;2(6):363–72.

177. Piercy V, Banner SE, Bhattacharyya A, Parsons AA, Sanger GJ, Smith SA, et al. Thermal, but not mechanical, nociceptive behavior is altered in the Zucker diabetic fatty rat and is independent of glycemic status. J Diabetes Complications. 1999;13(3):163–9.

178. Brand CL, Sturis J, Gotfredsen CF, Fleckner J, Fledelius C, Hansen BF, et al. Dual PPARα/γ activation provides enhanced improvement of insulin sensitivity and glycemic control in ZDF rats. Am J Physiol - Endocrinol Metab [Internet]. 2003 Apr 1;284(4):E841 LP-E854. Available from: http://ajpendo.physiology.org/content/284/4/E841.abstract

179. Chen X, Osborne MC, Rybczynski PJ, Zeck R, Yang M, Xu J, et al. Pharmacological profile of a novel, non-TZD PPARg agonist. Diabetes, Obes Metab [Internet]. 2005 Sep [cited 2017 Jun 8];7(5):536–46. Available from: http://doi.wiley.com/10.1111/j.1463-1326.2004.00425.x

180. Kanda S, Nakashima R, Takahashi K, Tanaka J, Ogawa J, Ogata T, et al. Potent antidiabetic effects of rivoglitazone, a novel peroxisome proliferator-activated receptor-gamma agonist, in obese diabetic rodent models. J Pharmacol Sci [Internet]. 2009;111(2):155–66. Available from: papers2://publication/uuid/394F75D2-8B90-47A1-89C1-884FA65FCEDF

181. Liu L, Wan J, Lang H, Si M, Zhu J, Zhou Y, et al. Dihydromyricetin delays the onset of hyperglycemia and ameliorates insulin resistance without excessive weight gain in Zucker diabetic fatty rats. Mol Cell Endocrinol [Internet]. 2017;439:105–15. Available from: http://dx.doi.org/10.1016/j.mce.2016.10.028

182. Beaudoin MS, Snook LA, Arkell AM, Stefanson A, Wan Z, Simpson JA, et al. Novel effects of rosiglitazone on SMAD2 and SMAD3 signaling in white adipose tissue of diabetic rats. Obesity. 2014;22(7):1632–42.

183. Nemanich S, Rani S, Shoghi K. In vivo multi-tissue efficacy of peroxisome proliferator-activated receptor-?? therapy on glucose and fatty acid metabolism in obese type 2 diabetic rats. Obesity. 2013;21(12):2522–9.

184. Schäfer HL, Linz W, Falk E, Glien M, Glombik H, Korn M, et al. AVE8134, a novel potent PPARα agonist, improves lipid profile and glucose metabolism in dyslipidemic mice and type 2 diabetic rats. Acta Pharmacol Sin [Internet]. 2012;33(1):82–90. Available from: http://dx.doi.org/10.1038/aps.2011.165

185. Rohrbach K, Thomas MA, Glick S, Fung EN, Wang V, Watson L, et al. Ibipinabant attenuates β-cell loss in male Zucker diabetic fatty rats independently of its effects on body weight. Diabetes, Obes Metab. 2012;14(6):555–64.

186. Lu X, Guo X, Karathanasis SK, Zimmerman KM, Onyia JE, Peterson RG, et al. Rosiglitazone reverses endothelial dysfunction but not remodeling of femoral artery in Zucker diabetic fatty rats. Cardiovasc Diabetol [Internet]. 2010;9:19. Available from: http://www.pubmedcentral.nih.gov/articlerender.fcgi?artid=2891691&tool=pmcentrez&rendertype=abstract

187. Oguma T, Kuriyama C, Nakayama K, Matsushita Y, Yoshida K, Kiuchi S, et al. The effect of combined treatment with canagliflozin and teneligliptin on glucose intolerance in Zucker diabetic fatty rats. J Pharmacol Sci [Internet]. 2015;127(4):456–61. Available from: http://dx.doi.org/10.1016/j.jphs.2015.03.006

188. Watanabe Y, Nakayama K, Taniuchi N, Horai Y, Kuriyama C, Ueta K, et al. Beneficial effects of canagliflozin in combination with pioglitazone on insulin sensitivity in rodent models of obese type 2 diabetes. PLoS One. 2015;10(1):1–15.

189. Kuriyama C, Xu JZ, Lee SP, Qi J, Kimata H, Kakimoto T, et al. Analysis of the effect of canagliflozin on renal glucose reabsorption and progression of hyperglycemia in zucker diabetic Fatty rats. J Pharmacol Exp Ther. 2014;351(2):423–31.

190. Liang Y, Arakawa K, Ueta K, Matsushita Y, Kuriyama C, Martin T, et al. Effect of canagliflozin on renal threshold for glucose, glycemia, and body weight in normal and diabetic animal models. PLoS One. 2012;7(2):2–8.

191. Ueta K, O’Brien TP, McCoy G a, Kim K, Healey EC, Farmer TD, et al. Glucotoxicity targets hepatic glucokinase in Zucker diabetic fatty rats, a model of type 2 diabetes associated with obesity. Am J Physiol Endocrinol Metab [Internet]. 2014;306(11):E1225-38. Available from: http://www.pubmedcentral.nih.gov/articlerender.fcgi?artid=4042096&tool=pmcentrez&rendertype=abstract

192. Ferreira L, Teixeira-De-Lemos E, Pinto F, Parada B, Mega C, Vala H, et al. Effects of sitagliptin treatment on dysmetabolism, inflammation, and oxidative stress in an animal model of type 2 diabetes (ZDF rat). Mediators Inflamm. 2010;2010:1–11.

193. Hansen HH, Jelsing J, Hansen CF, Hansen G, Vrang N, Mark M, et al. The sodium glucose cotransporter type 2 inhibitor empagliflozin preserves β-cell mass and restores glucose homeostasis in the male zucker diabetic fatty rat. J Pharmacol Exp Ther [Internet]. 2014;350(3):657–64. Available from: http://www.ncbi.nlm.nih.gov/pubmed/24993361

194. Yuen VG, Coleman J, Withers SG, Andersen RJ, Brayer GD, Mustafa S, et al. Glucose lowering effect of montbretin A in Zucker Diabetic Fatty rats. Mol Cell Biochem. 2016;411(1–2):373–81.

195. Sturis J, Pugh WL, Tang J, Polonsky KS. Prevention of diabetes does not completely prevent insulin secretory defects in the ZDF rat. Am J Physiol. 1995;269(4 Pt 1):E786-92.

196. Friedman JE, de Venté JE, Peterson RG, Dohm GL. Altered expression of muscle glucose transporter GLUT-4 in diabetic fatty Zucker rats (ZDF/Drt-fa). Am J Physiol [Internet]. 1991;261(6 Pt 1):E782-8. Available from: http://www.ncbi.nlm.nih.gov/pubmed/1767839

197. Dhalla a K, Santikul M, Chisholm JW, Belardinelli L, Reaven GM. Comparison of the antilipolytic effects of an A1 adenosine receptor partial agonist in normal and diabetic rats. Diabetes Obes Metab [Internet]. 2009;11(2):95–101. Available from: http://www.ncbi.nlm.nih.gov/pubmed/18494808

198. Dang Q, Kasibhatla SR, Reddy KR, Jiang T, Reddy MR, Potter SC, et al. Discovery of Potent and Specific Fructose-1,6-Bisphosphatase Inhibitors and a Series of Orally Bioavailable Phosphoramidase Sensitive Prodrugs for the Treatment of Type 2 Diabetes. J Am Chem Soc. 2007;.(1):1047–53.

199. Erion MD, van Poelje PD, Dang Q, Kasibhatla SR, Potter SC, Reddy MR, et al. MB06322 (CS-917): A potent and selective inhibitor of fructose 1,6-bisphosphatase for controlling gluconeogenesis in type 2 diabetes. Proc Natl Acad Sci U S A [Internet]. 2005;102(22):7970–5. Available from: http://www.pnas.org/content/102/22/7970.short

200. Van Poelje PD, Potter SC, Chandramouli VC, Landau BR, Dang Q, Erion MD. Inhibition of fructose 1,6-bisphosphatase reduces excessive endogenous glucose production and attenuates hyperglycemia in Zucker diabetic fatty rats. Diabetes. 2006;55(6):1747–54.

201. Hummel KP, Dickie MM, Coleman DL. Diabetes, a New Mutation in the Mouse. Science (80- ) [Internet]. 1966;153(3740):1127–8. Available from: http://www.sciencemag.org/content/153/3740/1127.abstract

202. The Jackson Laboratory. Mouse Strain Datasheet 000642 [Internet]. [cited 2017 Jun 22]. Available from: https://www.jax.org/strain/000642

203. Hummel KP, Coleman DL, Lane PW. The influence of genetic background on expression of mutations at the diabetes locus in the mouse. I. C57BL/KsJ and C57BL/6J strains. Biochem Genet. 1972;7(1):1–13.

204. Boquist L, Hellman B, Lernmark A, Täljedal IB. Influence of the mutation “diabetes” on insulin release and islet morphology in mice of different genetic backgrounds. J Cell Biol. 1974;62(1):77–89.

205. Coleman DL, Jackson T. Hyperinsulinemia in Pre-Weaning Diabetes (db) Mice. Diabetologia. 1974;10(Suppl 1):607–10.

206. Coleman DL. Diabetes-obesity syndromes in mice. Diabetes. 1982;31(Suppl. 1):1–6.

207. Coleman DL, Hummel KP. Studies with the mutation, diabetes, in the mouse. Diabetologia. 1967;3(2):238–48.

208. Berglund O, Frankel BJ, Hellman B. Development of the insulin secretory defect in genetically diabetic (db/db) mouse. Acta Endocrinol (Copenh). 1978;87(3):543–51.

209. Like AA, Chick WL. Studies in the diabetic mutant mouse: I. Light microscopy and radioautography of pancreatic islets. Diabetologia. 1970;6(3):207–15.

210. Like AA, Chick WL. Studies in the diabetic mutant mouse: II. Electron Microscopy of Pancreatic Islets. Diabetologia. 1970;6(3):216–42.

211. Chick WL, Like AA. Studies in the diabetic mutant mouse: III. Physiological factors associated with alterations in beta cell proliferation. Diabetologia. 1970;6(3):243–51.

212. Tuman RW, Doisy RJ. The Influence of Age on the Development of Hypertriglyceridaemia and Hypercholesterolaemia in Genetically Diabetic Mice. Diabetologia [Internet]. 1977 Jan;13(1):7–11. Available from: http://dx.doi.org/10.1007/BF00996320

213. Coleman DL, Hummel KP. Effects of parabiosis of normal with genetically diabetic mice. Am J Physiol. 1969;217(5):1298–304.

214. Wyse BM, Dulin WE. The influence of age and dietary conditions on diabetes in the db mouse. Diabetologia. 1970;6(3):268–73.

215. Malaisse WJ, Malaisse-Lagae F, Coleman DL. Insulin Secretion in Mice with an Hereditary Diabetes. Exp Biol Med. 1968;129(1):65–9.

216. Nishina PM, Lowe S, Wang J, Paigen B. Characterization of plasma lipids in genetically obese mice: The mutants obese, diabetes, fat, tubby, and lethal yellow. Metabolism. 1994;43(5):549–53.

217. Grounds MD, Radley HG, Lynch GS, Nagaraju K, De Luca A. Towards developing standard operating procedures for pre-clinical testing in the mdx mouse model of Duchenne muscular dystrophy. Neurobiol Dis. 2008;31(1):1–19.

218. Dutta S, Sengupta P. Men and mice: Relating their ages. Life Sci [Internet]. 2016;152:244–8. Available from: http://dx.doi.org/10.1016/j.lfs.2015.10.025

219. Like AA, Lavine RL, Poffenbarger PL, Chick WL. Studies in the Diabetic Mutant Mouse: VI. Evolution of Glomerular Lesions and Associated Proteinuria. Am J Pathol [Internet]. 1972;66(2):193–224. Available from: http://www.ncbi.nlm.nih.gov/pmc/articles/PMC2032492/pdf/amjpathol00550-0005.pdf

220. Gaertner K. Glomerular hyperfiltration during the onset of diabetes mellitus in two strains of diabetic mice (c57bl/6j db/db and c57bl/ksj db/db). Diabetologia [Internet]. 1978;15(1):59–63. Available from: http://www.ncbi.nlm.nih.gov/entrez/query.fcgi?cmd=Retrieve&db=PubMed&dopt=Citation&list_uids=98379

221. Meade CJ, Brandon DR, Smith W, Simmonds RG, Harris S, Sowter C. The relationship between hyperglycaemia and renal immune complex deposition in mice with inherited diabetes. Clin Exp Immunol [Internet]. 1981;43(1):109–20. Available from: http://www.ncbi.nlm.nih.gov/pubmed/7018755%5Cnhttp://www.ncbi.nlm.nih.gov/pmc/articles/PMC1537122/pdf/clinexpimmunol00184-0117.pdf

222. Kitada M, Kume S, Imaizumi N, Koya D. Resveratrol improves oxidative stress and protects against diabetic nephropathy through normalization of Mn-SOD dysfunction in AMPK/SIRT1- independent pathway. Diabetes. 2011;60(2):634–43.

223. Susztak K, Raff AC, Schiffer M, Böttinger EP. Glucose-Induced Reactive Oxygen Species Cause Apoptosis of Podocytes and Podocyte Depletion at the Onset of Diabetic Nephropathy. Diabetes [Internet]. 2006;55(1):225–33. Available from: http://diabetes.diabetesjournals.org.

224. Koya D, Haneda M, Nakagawa H, Isshiki K, Sato H, Maeda S, et al. Amelioration of accelerated diabetic mesangial expansion by treatment with a PKC beta inhibitor in diabetic db/db mice, a rodent model for type 2 diabetes. FASEB. 2000;14(3):439–47.

225. Midena E, Segato T, Radin S, Di Giorgio G, Meneghini F, Piermarocchi S, et al. Studies on the retina of the diabetic db/db mouse: I. endothelial cell-pericyte ratio. Ophthalmic Res. 1989;21(2):106–11.

226. Clements RS, Robison WG, Cohen MP. Anti-glycated albumin therapy ameliorates early retinal microvascular pathology in db/db mice. J Diabetes Complications. 1998;12(1):28–33.

227. Cheung AKH, Fung MKL, Lo ACY, Lam TTL, So KF, Chung SSM, et al. Aldose Reductase Deficiency Prevents Diabetes-Induced Blood-Retinal Barrier Breakdown, Apoptosis, and Glial Reactivation in the Retina of db/db Mice. Diabetes. 2005;54(11):3119–25.

228. Tadayoni R, Paques M, Gaudric A, Vicaut E. Erythrocyte and leukocyte dynamics in the retinal capillaries of diabetic mice. Exp Eye Res. 2003;77(4):497–504.

229. Li J, Wang JJ, Yu Q, Chen K, Mahadev K, Zhang SX. Inhibition of Reactive Oxygen Species by Lovastatin Downregulates Vascular Endothelial Growth Factor Expression and Ameliorates Blood Retinal Barrier Breakdown in db/db Mice. Diabetes. 2010;59(June):1528–38.

230. Cohen MP, Hud E, Shea E, Shearman CW. Vitreous Fluid of db/db Mice Exhibits Alterations in Angiogenic and Metabolic Factors Consistent with Early Diabetic Retinopathy. Ophthalmic Res [Internet]. 2008;40(1):5–9. Available from: http://www.karger.com/DOI/10.1159/000111151

231. Sima AAF, Robertson DM. Peripheral neuropathy in mutant diabetic mouse [C57BL/Ks(db/db)]. Acta Neuropathol [Internet]. 1978;41(2):85–9. Available from: http://www.embase.com/search/results?subaction=viewrecord&from=export&id=L9011209%5Cnhttp://dx.doi.org/10.1007/BF00689757%5Cnhttp://sfx.library.uu.nl/utrecht?sid=EMBASE&issn=00016322&id=doi:10.1007/BF00689757&atitle=Peripheral+neuropathy+in+mutant+diabeti

232. Robertson DM, Sima AAF. Diabetic neuropathy in the mutant mouse [C57BL/ks9(db/db)] - A morphometric study. Diabetes. 1980;29(1):60–7.

233. Hanker JS, Ambrose WW, Yates PE, Koch G, Carson KA. Peripheral Neuropathy in Mouse Hereditary Diabetes Mellitus *. Acta Neuropathol. 1980;51:145–53.

234. Sullivan KA, Hayes JM, Wiggin TD, Backus C, Su Oh S, Lentz SI, et al. Mouse models of diabetic neuropathy. Neurobiol Dis. 2007;28(3):276–85.

235. Moore SA, Peterson RG, Felten DL, Cartwright TR, O’Connor BL. Reduced sensory and motor conduction velocity in 25-week-old diabetic [ C57BL Ks ( db db)] mice. Exp Neurol. 1980;70(3):548–55.

236. PubMed. Tcf7l2 transcription factor 7 like 2, T cell specific, HMG box [Mus musculus (house mouse)] [Internet]. [cited 2017 Jul 5]. Available from: https://www.ncbi.nlm.nih.gov/gene/21416

237. PubMed. Kcnj11 potassium inwardly rectifying channel, subfamily J, member 11 [Mus musculus (house mouse)] [Internet]. [cited 2017 Jul 5]. Available from: https://www.ncbi.nlm.nih.gov/gene/16514

238. PubMed. Pparg peroxisome proliferator activated receptor gamma [Mus musculus (house mouse)] [Internet]. [cited 2017 Jul 5]. Available from: https://www.ncbi.nlm.nih.gov/gene/19016

239. Chen ZL, Shao WJ, Xu F, Liu L, Lin BS, Wei XH, et al. Acute Wnt pathway activation positively regulates leptin gene expression in mature adipocytes. Cell Signal [Internet]. 2015;27(3):587–97. Available from: http://dx.doi.org/10.1016/j.cellsig.2014.12.012

240. Shu L, Matveyenko A V., Kerr-Conte J, Cho JH, McIntosh CHS, Maedler K. Decreased TCF7L2 protein levels in type 2 diabetes mellitus correlate with downregulation of GIP- and GLP-1 receptors and impaired beta-cell function. Hum Mol Genet. 2009;18(13):2388–99.

241. Sharma M, Mohapatra J, Malik U, Nagar J, Chatterjee A, Ramachandran B, et al. Effect of pioglitazone on metabolic features in endotoxemia model in obese diabetic db/db mice. J Diabetes. 2017;9(6):613–21.

242. Singh VP, Gurunathan C, Singh S, Singh B, Lakshmi BJ, Mishra AP, et al. Genetic deletion of Wdr13 improves the metabolic phenotype of Lepr db/db mice by modulating AP1 and PPARγ target genes. Diabetologia. 2015;58(2):384–92.

243. Su W, Xie Z, Guo Z, Duncan MJ, Lutshumba J, Gong MC. Altered clock gene expression and vascular smooth muscle diurnal contractile variations in type 2 diabetic db/db mice. Am J Physiol Hear Circ Physiol [Internet]. 2012;302(3):H621-33. Available from: http://www.pubmedcentral.nih.gov/articlerender.fcgi?artid=3353796&tool=pmcentrez&rendertype=abstract

244. Chen H, Charlat O, Tartaglia LA, Woolf EA, Weng X, Ellis SJ, et al. Evidence that the diabetes gene encodes the leptin receptor: Identification of a mutation in the leptin receptor gene in db/db mice. Cell. 1996;84(3):491–5.

245. Tartaglia LA, Dembski M, Weng X, Deng N, Culpepper J, Devos R, et al. Identification and expression cloning of a leptin receptor, OB-R. Cell. 1995;83(7):1263–71.

246. Coleman DL. Effects of parabiosis of obese with diabetes and normal mice. Diabetologia [Internet]. 1973;9(4):294–8. Available from: http://link.springer.com/10.1007/BF01221857

247. Chlouverakis C. Insulin resistance of parabiotic obese-hyperglycemic mice. Horm Metab Res. 1972;1972(4):143–8.

248. Haessler HA, Crawford JD. Alterations in the Fatty Acid Composition of Depot Fat Associated With Obesity. Ann N Y Acad Sci. 1965;131(1):476–84.

249. Campfield LA, Smith FJ, Guisez Y, Devos R, Campfield LA, Smith FJ, et al. Recombinant Mouse OB Protein: Evidence for a Peripheral Signal Linking Adiposity and Central Neural Networks. Science (80- ). 1995;269(5223):546–9.

250. Halaas J, Gajiwala K, Maffei M, Cohen S, Chait B, Rabinowitz D, et al. Weight-reducing effects of the plasma protein encoded by the obese gene. Science (80- ) [Internet]. 1995;269(5223):543–6. Available from: http://www.sciencemag.org/cgi/doi/10.1126/science.7624777

251. Pelleymounter MA, Cullen MJ, Baker MB, Hecht R, Pelleymounter MA, Cullen MJ, et al. Effects of the Obese Gene Product on Body Weight Regulation in Ob/Ob Mice. Science (80- ). 1995;269(5223):540–3.

252. Stephens TW, Basinski M, Bristow PK, Bue-Valleskey JM, Burgett SG, Craft L, et al. The role of neuropeptide Y in the antiobesity action of the obese gene product. Vol. 377, Nature. 1995. p. 530–2.

253. Takada K, Kanatsuka A, Tokuyama Y, Yagui K, Nishimura M, Saito Y, et al. Islet amyloid polypeptide/amylin contents in pancreas change with increasing age in genetically obese and diabetic mice. Diabetes Res Clin Pract. 1996;33(3):153–8.

254. Chen F, Dong RR, Zhong KL, Ghosh A, Tang SS, Long Y, et al. Antidiabetic drugs restore abnormal transport of amyloid-β across the blood-brain barrier and memory impairment in db/db mice. Neuropharmacology [Internet]. 2016;101:123–36. Available from: http://dx.doi.org/10.1016/j.neuropharm.2015.07.023

255. Li DJ, Huang F, Lu WJ, Jiang GJ, Deng YP, Shen FM. Metformin promotes irisin release from murine skeletal muscle independently of AMP-activated protein kinase activation. Acta Physiol. 2015;213(3):711–21.

256. Bao Q, Shen X, Qian L, Gong C, Nie M, Dong Y. Anti-diabetic activities of catalpol in db/db mice. Korean J Physiol Pharmacol. 2016;20(2):153–60.

257. Asare GA, Adjei S, Afriyie D, Appiah-Danquawh AB, Asia J, Asiedu B, et al. Croton membranaceus improves some biomarkers of cardiovascular disease and diabetes in genetic animal models. J Clin Diagnostic Res. 2015;9(12):OF01–5.

258. Solini A, Rossi C, Duranti E, Taddei S, Natali A, Virdis A. Saxagliptin prevents vascular remodeling and oxidative stress in db/db mice. Role of endothelial nitric oxide synthase uncoupling and cyclooxygenase. Vascul Pharmacol [Internet]. 2016;76:62–71. Available from: http://dx.doi.org/10.1016/j.vph.2015.10.002

259. Kim D Il, Park MJ, Heo YR, Park SH. Metformin ameliorates lipotoxicity-induced mesangial cell apoptosis partly via upregulation of glucagon like peptide-1 receptor (GLP-1R). Arch Biochem Biophys [Internet]. 2015;584:90–7. Available from: http://dx.doi.org/10.1016/j.abb.2015.08.009

260. Wang X, Chen Y, Abdelkader D, Hassan W, Sun H, Liu J. Combination therapy with oleanolic acid and metformin as a synergistic treatment for diabetes. J Diabetes Res. 2015;2015:1–12.

261. Cheng L, Meng XB, Lu S, Wang TT, Liu Y, Sun GB, et al. Evaluation of Hypoglycemic Efficacy of Tangningtongluo Formula, a Traditional Chinese Miao Medicine, in Two Rodent Animal Models. J Diabetes Res. 2014;2014:1–12.

262. Somineni HK, Boivin GP, Elased KM. Daily exercise training protects against albuminuria and angiotensin converting enzyme 2 shedding in db/db diabetic mice. J Endocrinol. 2014;221(2):235–51.

263. Kim MH, Jee JH, Park S, Lee MS, Kim KW, Lee MK. Metformin enhances glucagon-like peptide 1 via cooperation between insulin and Wnt signaling. J Endocrinol. 2014;220(2):117–28.

264. Anthony J, Kelkar A, Wilankar C, Ranjith V, Bhumra SK, Mutt S, et al. Discovery of P1736, a Novel Antidiabetic Compound That Improves Peripheral Insulin Sensitivity in Mice Models. PLoS One. 2013;8(10):1–10.

265. Ly A, Scheerer MF, Zukunft S, Muschet C, Merl J, Adamski J, et al. Retinal proteome alterations in a mouse model of type 2 diabetes. Diabetologia. 2014;57(1):192–203.

266. Tang T, Reed MJ. Exercise adds to metformin and acarbose efficacy in db/db mice. Metabolism. 2001;50(9):1049–53.

267. Bruckbauer A, Zemel MB. Effects of metformin, resveratrol, and hydroxymethylbutyrate on insulin sensitivity. Diabetes, Metab Syndr Obes Targets Ther [Internet]. 2013;6:93–102. Available from: http://ovidsp.ovid.com/ovidweb.cgi?T=JS&PAGE=reference&D=medl&NEWS=N&AN=23704674

268. Tripp ML, Darland G, Konda VR, Pacioretty LM, Chang JL, Bland JS, et al. Optimized mixture of hops rho iso-alpha acids-rich extract and acacia proanthocyanidins- rich extract reduces insulin resistance in 3t3-l1 adipocytes and improves glucose and insulin control in db/db mice. Nutr Res Pract. 2012;6(5):405–13.

269. Li J, Deng J, Sheng W, Zuo Z. Metformin attenuates Alzheimer’s disease-like neuropathology in obese, leptin-resistant mice. Pharmacol Biochem Behav [Internet]. 2012;101(4):564–74. Available from: http://dx.doi.org/10.1016/j.pbb.2012.03.002

270. Caton PW, Kieswich J, Yaqoob MM, Holness MJ, Sugden MC. Metformin opposes impaired AMPK and SIRT1 function and deleterious changes in core clock protein expression in white adipose tissue of genetically-obese db/db mice. Diabetes Obes Metab. 2011;13(12):1097–104.

271. Akanksha, Srivastava AK, Maurya R. Antihyperglycemic activity of compounds isolated from indian medicinal plants. Indian J Exp Biol. 2010;48(3):294–8.

272. Caton PW, Nayuni NK, Kieswich J, Khan NQ, Yaqoob MM, Corder R. Metformin suppresses hepatic gluconeogenesis through induction of SIRT1 and GCN5. J Endocrinol. 2010;205(1):97–106.

273. Maurya R, Akanksha, Jayendra, Singh AB, Srivastava AK. Coagulanolide, a withanolide from Withania coagulans fruits and antihyperglycemic activity. Bioorganic Med Chem Lett [Internet]. 2008;18(24):6534–7. Available from: http://dx.doi.org/10.1016/j.bmcl.2008.10.050

274. Heishi M, Hayashi K, Ichihara J, Ishikawa H, Kawamura T, Kanaoka M, et al. Comparison of gene expression changes induced by biguanides in db/db mice liver. J Toxicol Sci [Internet]. 2008;33(3):339–47. Available from: http://eutils.ncbi.nlm.nih.gov/entrez/eutils/elink.fcgi?dbfrom=pubmed&id=18670165&retmode=ref&cmd=prlinks%5Cnpapers2://publication/uuid/3164A282-A794-4DCE-B8B0-C833C3E29D24

275. Yoon SH, Han EJ, Sung JH, Chung SH. Anti-diabetic effects of compound K versus metformin versus compound K-metformin combination therapy in diabetic db/db mice. Biol Pharm Bull [Internet]. 2007;30(11):2196–200. Available from: http://www.ncbi.nlm.nih.gov/pubmed/17978500

276. Heishi M, Ichihara J, Teramoto R, Itakura Y, Hayashi K, Ishikawa H, et al. Global gene expression analysis in liver of obese diabetic db/db mice treated with metformin. Diabetologia. 2006;49(7):1647–55.

277. Cheng L, Song J, Li G, Liu Y, Wang Y, Meng X, et al. Effects of the Tangningtongluo formula as an alternative strategy for diabetics via upregulation of insulin receptor substrate-1. Mol Med Rep. 2017;16(1):703–9.

278. Fujita H, Fujishima H, Koshimura J, Hosoba M, Yoshioka N, Shimotomai T, et al. Effects of antidiabetic treatment with metformin and insulin on serum and adipose tissue adiponectin levels in db/db mice. Endocr J. 2005;52(4):427–33.

279. Fujita H, Fujishima H, Morii T, Koshimura J, Narita T, Kakei M, et al. Effect of metformin on adipose tissue resistin expression in db / db mice. Biochem Biophys Res Commun. 2002;298(3):345–9.

280. Ono M, Itakura Y, Nonomura T, Nakagawa T, Nakayama C, Taiji M, et al. Intermittent administration of brain-derived neurotrophic factor ameliorates glucose metabolism in obese diabetic mice. Metabolism [Internet]. 2000;49(1):129–33. Available from: http://www.sciencedirect.com/science/article/pii/S0026049500909880

281. Raza S, Srivastava SP, Srivastava DS, Srivastava AK, Haq W, Katti SB. Thiazolidin-4-one and thiazinan-4-one derivatives analogous to rosiglitazone as potential antihyperglycemic and antidyslipidemic agents. Eur J Med Chem [Internet]. 2013;63:611–20. Available from: http://dx.doi.org/10.1016/j.ejmech.2013.01.054

282. Dludla P V., Muller CJF, Joubert E, Louw J, Essop MF, Gabuza KB, et al. Aspalathin protects the heart against hyperglycemia-induced oxidative damage by up-regulating Nrf2 expression. Molecules. 2017;22(1):1–16.

283. Huang MQ, Zhou CJ, Zhang YP, Zhang XQ, Xu W, Lin J, et al. Salvianolic Acid B Ameliorates Hyperglycemia and Dyslipidemia in db/db Mice through the AMPK Pathway. Cell Physiol Biochem. 2016;40(5):933–43.

284. Bae U-J, Choi E-K, Oh M-R, Jung S-J, Park J, Jung T-S, et al. Angelica gigas Ameliorates Hyperglycemia and Hepatic Steatosis in C57BL/KsJ-db/db Mice via Activation of AMP-Activated Protein Kinase Signaling Pathway. Am J Chin Med. 2016;44(8):1627–38.

285. Adam J, Brandmaier S, Leonhardt J, Scheerer MF, Mohney RP, Xu T, et al. Metformin effect on nontargeted metabolite profiles in patients with type 2 diabetes and in multiple murine tissues. Diabetes. 2016;65(12):3776–85.

286. Mitchell PL, Nachbar R, Lachance D, St-Pierre P, Trottier J, Barbier O, et al. Treatment with a novel agent combining docosahexaenoate and metformin increases protectin DX and IL-6 production in skeletal muscle and reduces insulin resistance in obese diabetic db/db mice. Diabetes, Obes Metab. 2017;19(3):313–9.

287. Gallo LA, Ward MS, Fotheringham AK, Zhuang A, Borg DJ, Flemming NB, et al. Erratum: Once daily administration of the SGLT2 inhibitor, empagliflozin, attenuates markers of renal fibrosis without improving albuminuria in diabetic db/db mice. Sci Rep [Internet]. 2016;6(January):26428. Available from: http://www.ncbi.nlm.nih.gov/pubmed/27389496%5Cnhttp://www.pubmedcentral.nih.gov/articlerender.fcgi?artid=PMC4936511

288. Kushwaha RN, Srivastava R, Mishra A, Rawat AK, Srivastava AK, Haq W, et al. Design, Synthesis, Biological Screening, and Molecular Docking Studies of Piperazine-Derived Constrained Inhibitors of DPP-IV for the Treatment of Type 2 Diabetes. Chem Biol Drug Des [Internet]. 2015;85(4):439–46. Available from: http://www.ncbi.nlm.nih.gov/pubmed/25216392

289. Zhao SJ, Wang DH, Li YW, Han L, Xiao X, Ma M, et al. A novel selective VPACAC2 agonist peptide-conjugated chitosan modified selenium nanoparticles with enhanced anti-type 2 diabetes synergy effects. Int J Nanomedicine. 2017;12:2143–60.

290. Hernández C, Bogdanov P, Corraliza L, García-Ramírez M, Solà-Adell C, Arranz JA, et al. Topical administration of GLP-1 receptor agonists prevents retinal neurodegeneration in experimental diabetes. Diabetes. 2016;65(1):172–87.

291. Rieg T, Gerasimova M, Murray F, Masuda T, Tang T, Rose M, et al. Natriuretic effect by exendin-4, but not the DPP-4 inhibitor alogliptin, is mediated via the GLP-1 receptor and preserved in obese type 2 diabetic mice. Am J Physiol Ren Physiol [Internet]. 2012;303(7):F963-71. Available from: http://www.ncbi.nlm.nih.gov/pubmed/22832924%5Cnhttp://ajprenal.physiology.org/content/ajprenal/303/7/F963.full.pdf

292. Patel V, Joharapurkar A, Gandhi T, Patel K, Dhanesha N, Kshirsagar S, et al. Omeprazole improves anti-obesity and antidiabetic effects of exendin-4 in db/db mice. J Diabetes. 2013;5(2):163–71.

293. Kan M, Guo G, Singh B, Singh V, Zochodne DW. Glucagon-Like Peptide 1, Insulin, Sensory Neurons, and Diabetic Neuropathy. J Neuropathol Exp Neurol. 2012;71(6):494–510.

294. Dhanesha N, Joharapurkar A, Shah G, Dhote V, Kshirsagar S, Bahekar R, et al. Exendin-4 reduces glycemia by increasing liver glucokinase activity: An insulin independent effect. Pharmacol Reports. 2012;64(1):140–9.

295. Liu BY, Jiang Y, Liu Z, Li S, Debing LU, Chen B. Down-regulation of zinc transporter 8 in the pancreas of db/db mice is rescued by Exendin-4 administration. Mol Med Rep. 2011;4(1):47–52.

296. Hamilton A, Patterson S, Porter D, Gault VA, Holscher C. Novel GLP-1 mimetics developed to treat type 2 diabetes promote progenitor cell proliferation in the brain. J Neurosci Res. 2011;89(4):481–9.

297. Gedulin BR, Smith PA, Jodka CM, Chen K, Bhavsar S, Nielsen LL, et al. Pharmacokinetics and pharmacodynamics of exenatide following alternate routes of administration. Int J Pharm. 2008;356(1–2):231–8.

298. Park CW, Kim HW, Ko SH, Lim JH, Ryu GR, Chung HW, et al. Long-Term Treatment of Glucagon-Like Peptide-1 Analog Exendin-4 Ameliorates Diabetic Nephropathy through Improving Metabolic Anomalies in db/db Mice. J Am Soc Nephrol [Internet]. 2007;18(4):1227–38. Available from: http://www.jasn.org/cgi/doi/10.1681/ASN.2006070778

299. Wang Q, Brubaker P. Glucagon-like peptide-1 treatment delays the onset of diabetes in 8 week-old db/db mice. Diabetologia. 2002;45(9):1263–73.

300. Rolin B, Larsen MO, Gotfredsen CF, Deacon CF, Carr RD, Wilken M, et al. The long-acting GLP-1 derivative NN2211 ameliorates glycemia and increases beta-cell mass in diabetic mice. Am J Physiol Endocrinol Metab [Internet]. 2002;283(4):E745-52. Available from: http://www.ncbi.nlm.nih.gov/pubmed/12217892

301. Hou S, Li C, Huan Y, Liu S, Liu Q, Sun S, et al. Effects of E2HSA, a long-acting glucagon like peptide-1 receptor agonist, on glycemic control and beta cell function in spontaneous diabetic db/db mice. J Diabetes Res. 2015;2015:1–17.

302. Nakashima R, Yano T, Ogawa J, Tanaka N, Toda N, Yoshida M, et al. Potentiation of insulin secretion and improvement of glucose intolerance by combining a novel G protein-coupled receptor 40 agonist DS-1558 with glucagon-like peptide-1 receptor agonists. Eur J Pharmacol [Internet]. 2014;737:194–201. Available from: http://dx.doi.org/10.1016/j.ejphar.2014.05.014

303. Liang R, Li X, Zhang R, Shi Y, Wang A, Chen D, et al. Acylation of exenatide by glycolic acid and its anti-diabetic activities in db/db mice. Pharm Res. 2014;31(8):1958–66.

304. Zhang B, He D, Fan Y, Liu N, Chen Y. Oral delivery of exenatide via microspheres prepared by cross-linking of alginate and hyaluronate. PLoS One. 2014;9(1):1–7.

305. Yu L, Li K, Liu X, Chen C, Bao Y, Ci T, et al. In vitro and in vivo evaluation of a once-weekly formulation of an antidiabetic peptide drug exenatide in an injectable thermogel. J Pharm Sci [Internet]. 2013;102(11):4140–9. Available from: http://dx.doi.org/10.1002/jps.23735

306. Dhanesha N, Joharapurkar A, Shah G, Kshirsagar S, Patel V, Patel K, et al. Treatment with exendin-4 improves the antidiabetic efficacy and reverses hepatic steatosis in glucokinase activator treated db/db mice. Eur J Pharmacol [Internet]. 2013;714(1–3):188–92. Available from: http://dx.doi.org/10.1016/j.ejphar.2013.06.015

307. Fosgerau K, Jessen L, Lind Tolborg J, Østerlund T, Schæffer Larsen K, Rolsted K, et al. The novel GLP-1-gastrin dual agonist, ZP3022, increases β-cell mass and prevents diabetes in db/db mice. Diabetes, Obes Metab. 2013;15(1):62–71.

308. Liu HY, Chung C, Yang W, Liang C, Wang C, Chang C, et al. Exendin-4 improves resistance to Listeria monocytogenes infection in diabetic db/db mice. J Vet Sci. 2012;13(3):245–52.

309. Yoshida S, Ohishi T, Matsui T, Tanaka H, Oshima H, Yonetoku Y, et al. The role of small molecule GPR119 agonist, AS1535907, in glucose-stimulated insulin secretion and pancreatic β-cell function. Diabetes Obes Metab [Internet]. 2011;13(1):34–41. Available from: http://www.ncbi.nlm.nih.gov/pubmed/21114601

310. Choi KH, Lee HA, Park MH, Han J-S. Mulberry (Morus alba L.) Fruit Extract Containing Anthocyanins Improves Glycemic Control and Insulin Sensitivity via Activation of AMP-Activated Protein Kinase in Diabetic C57BL/Ksj-db/db Mice. J Med Food [Internet]. 2016;19(8):737–45. Available from: http://search.ebscohost.com/login.aspx?direct=true&db=jlh&AN=117697372&site=ehost-live

311. Yuan X, Li H, Bai H, Zhao X, Zhang C, Liu H, et al. The 11β-hydroxysteroid dehydrogenase type 1 inhibitor protects against the insulin resistance and hepatic steatosis in db/db mice. Eur J Pharmacol [Internet]. 2016;788:140–51. Available from: http://dx.doi.org/10.1016/j.ejphar.2016.05.034

312. Feng L, Luo H, Xu Z, Yang Z, Du G, Zhang Y, et al. Bavachinin, as a novel natural pan-PPAR agonist, exhibits unique synergistic effects with synthetic PPAR-γ and PPAR-α agonists on carbohydrate and lipid metabolism in db/db and diet-induced obese mice. Diabetologia. 2016;59(6):1276–86.

313. Saito T, Hasegawa-Moriyama M, Yamada T, Kurimoto T, Inada E, Kanmura Y. Resolution of inflammation by resolvin D1 is essential for peroxisome proliferator-Activated receptor-γ-mediated analgesia during postincisional pain development in type 2 diabetes. Anesthesiology. 2015;123(6):1420–34.

314. Park MH, Nam YH, Han JS. Sargassum coreanum extract alleviates hyperglycemia and improves insulin resistance in db/db diabetic mice. Nutr Res Pract. 2015;9(5):472–9.

315. Park MH, Han J-S. *Padina arborescens* Ameliorates Hyperglycemia and Dyslipidemia in C57BL/KsJ- *db/db* Mice, a Model of Type 2 Diabetes Mellitus. J Med Food [Internet]. 2015;18(10):1088–94. Available from: http://online.liebertpub.com/doi/10.1089/jmf.2014.3375

316. Gim HJ, Li H, Jeong JH, Lee SJ, Sung M-K, Song M-Y, et al. Design, synthesis, and biological evaluation of a series of alkoxy-3-indolylacetic acids as peroxisome proliferator-activated receptor gamma/delta agonists. Bioorg Med Chem [Internet]. 2015;23(13):3322–36. Available from: http://ovidsp.ovid.com/ovidweb.cgi?T=JS&PAGE=reference&D=medl&NEWS=N&AN=25982078

317. Sharma BR, Kim HJ, Rhyu DY. Caulerpa lentillifera extract ameliorates insulin resistance and regulates glucose metabolism in C57BL/KsJ-db/db mice via PI3K/AKT signaling pathway in myocytes. J Transl Med. 2015;13(62):1–10.

318. Kotlinowski J, Grochot-Przeczek A, Taha H, Kozakowska M, Pilecki B, Skrzypek K, et al. PPARγ activation but not PPARγ haplodeficiency affects proangiogenic potential of endothelial cells and bone marrow-derived progenitors. Cardiovasc Diabetol [Internet]. 2014 Nov;13(1):150. Available from: http://www.pubmedcentral.nih.gov/articlerender.fcgi?artid=4233236&tool=pmcentrez&rendertype=abstract

319. Hanf R, Millatt LJ, Cariou B, Noel B, Rigou G, Delataille P, et al. The dual peroxisome proliferator-activated receptor alpha/delta agonist GFT505 exerts anti-diabetic effects in db/db mice without peroxisome proliferator-activated receptor gamma-associated adverse cardiac effects. Diab Vasc Dis Res [Internet]. 2014;11(6):440–7. Available from: http://www.ncbi.nlm.nih.gov/pubmed/25212694

320. Mishra P, Singh SV, Verma AK, Srivastava P, Sultana S, Rath SK. Rosiglitazone induces cardiotoxicity by accelerated apoptosis. Cardiovasc Toxicol. 2014;14(2):99–119.

321. Yang H-I, Kim WS, Kim D-H, Kang JS. Histopathological Evaluation of Heart Toxicity of a Novel Selective PPAR-gamma Agonists CKD-501 in db/db Mice. Biomol Ther (Seoul). 2013;21(1):84–8.

322. Ye H, Zhang HJ, Xu A, Hoo RLC. Resistin Production from Adipose Tissue Is Decreased in db/db Obese Mice, and Is Reversed by Rosiglitazone. PLoS One. 2013;8(6):1–8.

323. Chodavarapu H, Grobe N, Somineni HK, Salem ESB, Madhu M, Elased KM. Rosiglitazone Treatment of Type 2 Diabetic db/db Mice Attenuates Urinary Albumin and Angiotensin Converting Enzyme 2 Excretion. PLoS One. 2013;8(4):1–12.

324. Zhou Y, Jia S, Wang C, Chen Z, Chi Y, Li J, et al. FAM3A is a target gene of peroxisome proliferator-activated receptor gamma. Biochim Biophys Acta [Internet]. 2013;1830(8):4160–70. Available from: http://www.ncbi.nlm.nih.gov/pubmed/23562554

325. Wein S, Schrader E, Rimbach G, Wolffram S. Oral green tea catechins transiently lower plasma glucose concentrations in female db/db mice. J Med Food [Internet]. 2013;16(4):312–7. Available from: http://www.ncbi.nlm.nih.gov/pubmed/23514230

326. Shi D, Guo S, Jiang B, Guo C, Wang T, Zhang L, et al. HPN, a synthetic analogue of bromophenol from red alga Rhodomela confervoides: Synthesis and anti-diabetic effects in C57BL/KsJ-db/db mice. Mar Drugs. 2013;11(2):350–62.

327. He BK, Ning ZQ, Li ZB, Shan S, Pan DS, Ko BCB, et al. In vitro and in vivo characterizations of chiglitazar, a newly identified PPAR pan-agonist. PPAR Res. 2012;2012:1–13.

328. Schrader E, Wein S, Kristiansen K, Christensen LP, Rimbach G, Wolffram S. Plant Extracts of Winter Savory, Purple Coneflower, Buckwheat and Black Elder Activate PPAR-γ in COS-1 Cells but do not Lower Blood Glucose in Db/db Mice In vivo. Plant Foods Hum Nutr. 2012;67(4):377–83.

329. Do GM, Jung UJ, Park HJ, Kwon EY, Jeon SM, Mcgregor RA, et al. Resveratrol ameliorates diabetes-related metabolic changes via activation of AMP-activated protein kinase and its downstream targets in db/db mice. Mol Nutr Food Res. 2012;56(8):1282–91.

330. Park HJ, Jung UJ, Cho SJ, Jung HK, Shim S, Choi MS. Citrus unshiu peel extract ameliorates hyperglycemia and hepatic steatosis by altering inflammation and hepatic glucose- and lipid-regulating enzymes in db/db mice. J Nutr Biochem [Internet]. 2013;24(2):419–27. Available from: http://dx.doi.org/10.1016/j.jnutbio.2011.12.009

331. Tan Y, Muise ES, Dai H, Raubertas R, Wong KK, Thompson GM, et al. Novel Transcriptome Profiling Analyses Demonstrate that Selective PPARgamma Modulators Display Attenuated and Selective Gene Regulatory Activity in Comparison with PPARgamma Full Agonists. MolPharmacol. 2012;82(1):68–79.

332. Park CJ, Lee H-A, Han J-S. Jicama (Pachyrhizus erosus) extract increases insulin sensitivity and regulates hepatic glucose in C57BL/Ksj-db/db mice. J Clin Biochem Nutr. 2016;58(1):56–63.

333. Ortsater H, Grankvist N, Wolfram S, Kuehn N, Sjoholm A. Diet supplementation with green tea extract epigallocatechin gallate prevents progression to glucose intolerance in db/db mice. Nutr Metab [Internet]. 2012;9:11. Available from: https://www.ncbi.nlm.nih.gov/pmc/articles/PMC3298777/pdf/1743-7075-9-11.pdf

334. Sharma a. N, Elased KM, Lucot JB. Rosiglitazone treatment reversed depression- but not psychosis-like behavior of db/db diabetic mice. J Psychopharmacol [Internet]. 2012;26(5):724–32. Available from: http://www.ncbi.nlm.nih.gov/pubmed/22331176

335. Lee S-H, Min K-H, Han J-S, Lee D-H, Park D-B, Jung W-K, et al. Effects of brown alga, Ecklonia cava on glucose and lipid metabolism in C57BL/KsJ-db/db mice, a model of type 2 diabetes mellitus. Food Chem Toxicol [Internet]. 2012;50(3–4):575–82. Available from: http://dx.doi.org/10.1016/j.fct.2011.12.032

336. Tang YH, Sun ZL, Fan MS, Li ZX, Huang CG. Anti-Diabetic Effects of TongGuanWan, a Chinese Traditional Herbal Formula, in C57BL/KsJ-db / db Mice. Planta Med. 2012;78(1):18–23.

337. Oishi K, Tomita T, Itoh N, Ohkura N. PPARγ activation induces acute PAI-1 gene expression in the liver but not in adipose tissues of diabetic model mice. Thromb Res [Internet]. 2011;128(5):e81–5. Available from: http://dx.doi.org/10.1016/j.thromres.2011.06.020

338. Keil S, Matter H, Schönafinger K, Glien M, Mathieu M, Marquette J-P, et al. Sulfonylthiadiazoles with an unusual binding mode as partial dual peroxisome proliferator-activated receptor (PPAR) γ/δ agonists with high potency and in vivo efficacy. ChemMedChem [Internet]. 2011;6(4):633–53. Available from: http://www.ncbi.nlm.nih.gov/pubmed/21400663

339. Le Bouter S, Rodriguez M, Guigal-Stephan N, Courtade-Gaïani S, Xuereb L, De Montrion C, et al. Coordinate transcriptomic and metabolomic effects of the insulin sensitizer rosiglitazone on fundamental metabolic pathways in liver, soleus muscle, and adipose tissue in diabetic db/db mice. PPAR Res. 2010;2010:1–17.

340. Yang KJ, Noh JR, Kim YH, Gang GT, Hwang JH, Yang SJ, et al. Differential modulatory effects of rosiglitazone and pioglitazone on white adipose tissue in db/db mice. Life Sci [Internet]. 2010;87(13–14):405–10. Available from: http://dx.doi.org/10.1016/j.lfs.2010.08.002

341. Seto S, Okada K, Kiyota K, Isogai S, Iwago M, Shinozaki T, et al. Design, synthesis, and structure-activity relationship studies of novel 2,4,6-trisubstituted-5-pyrimidinecarboxylic acids as peroxisome proliferator-activated receptor γ (PPARγ) partial agonists with comparable antidiabetic efficacy to rosiglitazone. J Med Chem. 2010;53(13):5012–24.

342. Jung UJ, Baek N-I, Chung H-G, Jeong T-S, Lee KT, Lee M-K, et al. Antilipogenic and hypolipidemic effects of ethanol extracts from two variants of Artemisia princeps Pampanini in obese diabetic mice. J Med Food. 2009;12(6):1238–44.

343. Bak EJ, Park HG, Kim JM, Yoo Y-J, Cha J-H. Inhibitory effect of evodiamine alone and in combination with rosiglitazone on in vitro adipocyte differentiation and in vivo obesity related to diabetes. Int J Obes (Lond) [Internet]. 2010;34(2):250–60. Available from: http://www.ncbi.nlm.nih.gov/pubmed/19859078

344. Cheng L, Han X, Shi Y. A regulatory role of LPCAT1 in the synthesis of inflammatory lipids, PAF and LPC, in the retina of diabetic mice. Am J Physiol Endocrinol Metab [Internet]. 2009;297(6):E1276–82. Available from: http://eutils.ncbi.nlm.nih.gov/entrez/eutils/elink.fcgi?dbfrom=pubmed&id=19773578&retmode=ref&cmd=prlinks%5Cnpapers3://publication/doi/10.1152/ajpendo.00475.2009

345. Au WS, Lu LW, Tam S, Ko OKH, Chow BKC, He ML, et al. Pluronic L-81 ameliorates diabetic symptoms in db/db mice through transcriptional regulation of microsomal triglyceride transfer protein. World J Gastroenterol. 2009;15(24):2987–94.

346. Yun SI, Park HO, Kang JH. Effect of Lactobacillus gasseri BNR17 on blood glucose levels and body weight in a mouse model of type 2 diabetes. J Appl Microbiol. 2009;107(5):1681–6.

347. Wu HS, Zhu DF, Zhou CX, Feng CR, Lou YJ, Yang B, et al. Insulin sensitizing activity of ethyl acetate fraction of Acorus calamus L. in vitro and in vivo. J Ethnopharmacol. 2009;123(2):288–92.

348. Hui X, Zhu W, Wang Y, Lam KSL, Zhang J, Wu D, et al. Major urinary protein-1 increases energy expenditure and improves glucose intolerance through enhancing mitochondrial function in skeletal muscle of diabetic mice. J Biol Chem. 2009;284(21):14050–7.

349. Park SY, Shin HK, Lee JH, Kim CD, Lee WS, Rhim BY, et al. Cilostazol Ameliorates Metabolic Abnormalities with Suppression of Proinflammatory Markers in a db/db Mouse Model of Type 2 Diabetes via Activation of Peroxisome Proliferator-Activated Receptor ␥ Transcription □. Pharmacology. 2009;329(2):571–9.

350. Yeo J, Kang Y-J, Jeon S-M, Jung UJ, Lee M-K, Song H, et al. Potential Hypoglycemic Effect of an Ethanol Extract of *Gynostemma pentaphyllum* in C57BL/KsJ- *db/db* Mice. J Med Food [Internet]. 2008;11(4):709–16. Available from: http://www.liebertonline.com/doi/abs/10.1089/jmf.2007.0148

351. Wilson KD, Li Z, Wagner R, Yue P, Tsao P, Nestorova G, et al. Transcriptome alteration in the diabetic heart by rosiglitazone: Implications for cardiovascular mortality. PLoS One. 2008;3(7):1–11.

352. Kim D-J, Jeong Y-J, Kwon J-H, Moon K-D, Kim H-J, Jeon S-M, et al. Beneficial Effect of Chungkukjang on Regulating Blood Glucose and Pancreatic β-Cell Functions in C75BL/KsJ-db/db Mice. J Med FOOD J Med Food. 2008;11(2):215–23.

353. Chang CH, McNamara LA, Wu MS, Muise ES, Tan Y, Wood HB, et al. A novel selective peroxisome proliferator-activator receptor-γ modulator-SPPARγM5 improves insulin sensitivity with diminished adverse cardiovascular effects. Eur J Pharmacol. 2008;584(1):192–201.

354. Yeo J, Lee YH, Jeon SM, Jung UJ, Lee MK, Jung YM, et al. Supplementation of a novel microbial biopolymer, PGB1, from new Enterobacter sp. BL-2 delays the deterioration of type 2 diabetic mice. J Microbiol Biotechnol. 2007;17(12):1983–90.

355. Jung UJ, Baek NI, Chung HG, Bang MH, Jeong TS, Tae Lee K, et al. Effects of the ethanol extract of the roots of Brassica rapa on glucose and lipid metabolism in C57BL/KsJ-db/db mice. Clin Nutr. 2008;27(1):158–67.

356. Yamanaka M, Itakura Y, Tsuchida a, Nakagawa T, Noguchi H, Taiji M. Comparison of the antidiabetic effects of brain-derived neurotrophic factor and thiazolidinediones in obese diabetic mice. Diabetes Obes Metab. 2007;9(6):879–88.

357. Koh EH, Park JY, Park HS, Jeon MJ, Ryu JW, Kim M, et al. Essential role of mitochondrial function in adiponectin synthesis in adipocytes. Diabetes. 2007;56(12):2973–81.

358. How O-J, Larsen TS, Hafstad a D, Khalid A, Myhre ESP, Murray a J, et al. Rosiglitazone treatment improves cardiac efficiency in hearts from diabetic mice. Arch Physiol Biochem. 2007;113(4–5):211–20.

359. Jung UJ, Baek NI, Chung HG, Bang MH, Yoo JS, Jeong TS, et al. The anti-diabetic effects of ethanol extract from two variants of Artemisia princeps Pampanini in C57BL/KsJ-db/db mice. Food Chem Toxicol. 2007;45(10):2022–9.

360. Xiong WT, Gu L, Wang C, Sun HX, Liu X. Anti-hyperglycemic and hypolipidemic effects of Cistanche tubulosa in type 2 diabetic db/db mice. J Ethnopharmacol. 2013;150(3):935–45.

361. Roy S, Khanna V, Mittra S, Dhar A, Singh S, Mahajan DC, et al. Combination of dipeptidylpeptidase IV inhibitor and low dose thiazolidinedione: Preclinical efficacy and safety in db/db mice. Life Sci. 2007;81(1):72–9.

362. Okamoto Y, Higashiyama H, Rong JX, McVey MJ, Kinoshita M, Asano S, et al. Comparison of mitochondrial and macrophage content between subcutaneous and visceral fat in db/db mice. Exp Mol Pathol. 2007;83(1):73–83.

363. Mittra S, Sangle G, Tandon R, Sharma S, Roy S, Khanna V, et al. Increase in weight induced by muraglitazar, a dual PPARalpha/gamma agonist, in db/db mice: adipogenesis/or oedema? Br J Pharmacol [Internet]. 2007;150(4):480–7. Available from: http://www.ncbi.nlm.nih.gov/pubmed/17211457%5Cnhttp://www.pubmedcentral.nih.gov/articlerender.fcgi?artid=PMC2189717

364. Han HO, Koh JS, Kim SH, Park OK, Kim KH, Jeon SK, et al. Design and synthesis of Oxime ethers of α-acyl-β-phenylbutanoic acids as PPAR α and -γ dual agonists. Bioorg Med Chem Lett. 2007;17(4):937–41.

365. Wang X, Hu Z, Hu J, Du J, Mitch WE. Insulin resistance accelerates muscle protein degradation: Activation of the ubiquitin-proteasome pathway by defects in muscle cell signaling. Endocrinology. 2006;147(9):4160–8.

366. Choo HJ, Kim JH, Kwon OB, Lee CS, Mun JY, Han SS, et al. Mitochondria are impaired in the adipocytes of type 2 diabetic mice. Diabetologia. 2006;49(4):784–91.

367. Loffler M, Bilban M, Reimers M, Waldhäusl W, Stulnig TM. Blood glucose-lowering nuclear receptor agonists only partially normalize hepatic gene expression in db/db mice. J Pharmacol Exp Ther [Internet]. 2006;316(2):797–804. Available from: http://www.ncbi.nlm.nih.gov/pubmed/16260581

368. Dropinski JF, Akiyama T, Einstein M, Habulihaz B, Doebber T, Berger JP, et al. Synthesis and biological activities of novel aryl indole-2-carboxylic acid analogs as PPARγ partial agonists. Bioorganic Med Chem Lett. 2005;15(22):5035–8.

369. Shi GQ, Dropinski JF, McKeever BM, Xu S, Becker JW, Berger JP, et al. Design and synthesis of alpha-aryloxyphenylacetic acid derivatives: a novel class of PPARalpha/gamma dual agonists with potent antihyperglycemic and lipid modulating activity. J Med Chem [Internet]. 2005;48(13):4457–68. Available from: http://www.ncbi.nlm.nih.gov/pubmed/15974597

370. Li DD, Chen JH, Chen Q, Li GW, Chen J, Yue JM, et al. Swietenia mahagony extract shows agonistic activity to PPARγ and gives ameliorative effects on diabetic db/db mice. Acta Pharmacol Sin. 2005;26(2):220–2.

371. Combs TP, Wagner J JAB, Doebber T, Wang WJ, Zhang BB, Tanen M, et al. Induction of adipocyte complement related protein of 30 kilodaltons by PPAR-gamma agnoists: a potential mechanism of insulin sensitization. Endocrinology. 2002;143(September):998–1007.

372. Minoura H, Takeshita S, Ita M, Hirosumi J, Mabuchi M, Kawamura I, et al. Pharmacological characteristics of a novel nonthiazolidinedione insulin sensitizer, FK614. Eur J Pharmacol. 2004;494(2–3):273–81.

373. Kast-Woelbern HR, Dana SL, Cesario RM, Sun L, De Grandpre LY, Brooks ME, et al. Rosiglitazone induction of Insig-1 in white adipose tissue reveals a novel interplay of peroxisome proliferator-activated receptor γ and sterol regulatory element-binding protein in the regulation of adipogenesis. J Biol Chem. 2004;279(23):23908–15.

374. Arlt W, Neogi P, Gross C, Miller WL. Cinnamic acid based thiazolidinediones inhibit human P450c17 and 3 beta-hydroxysteroid dehydrogenase and improve insulin sensitivity independent of PPAR gamma agonist activity. J Mol Endocrinol. 2004;32:425–36.

375. Shen Q, Cline GW, Shulman GI, Leibowitz MD, Davies PJA. Effects of Rexinoids on Glucose Transport and Insulin-mediated Signaling in Skeletal Muscles of Diabetic (db/db) Mice. J Biol Chem. 2004;279(19):19721–31.

376. Bagi Z, Koller A, Kaley G. PPARgamma activation, by reducing oxidative stress, increases NO bioavailability in coronary arterioles of mice with Type 2 diabetes. Am J Physiol Heart Circ Physiol [Internet]. 2004;286(2):H742–8. Available from: http://www.ncbi.nlm.nih.gov/pubmed/14551045

377. Desai RC, Han W, Metzger EJ, Bergman JP, Gratale DF, MacNaul KL, et al. 5-Aryl thiazolidine-2,4-diones: Discovery of PPAR dual α/γ agonists as antidiabetic agents. Bioorganic Med Chem Lett. 2003;13(16):2795–8.

378. Misra P, Chakrabarti R, Vikramadithyan RK, Bolusu G, Juluri S, Hiriyan J, et al. PAT5A: a partial agonist of peroxisome proliferator-activated receptor gamma is a potent antidiabetic thiazolidinedione yet weakly adipogenic. J Pharmacol Exp Ther. 2003;306(2):763–71.

379. Koyama H, Boueres JK, Han W, Metzger EJ, Bergman JP, Gratale DF, et al. 5-Aryl thiazolidine-2,4-diones as selective PPARγ agonists. Bioorganic Med Chem Lett. 2003;13(10):1801–4.

380. Edwards D. Non-linear normalization and background correction in one-channel cDNA microarray studies. Bioinformatics. 2003;19(7):825–33.

381. Adams AD, Yuen W, Hu Z, Santini C, Jones AB, MacNaul KL, et al. Amphipathic 3-phenyl-7-propylbenzisoxazoles; human PPaR γ, δ and α agonists. Bioorganic Med Chem Lett. 2003;13(5):931–5.

382. Lejnev K, Khomsky L, Bokvist K, Mistriel-Zarbib S, Naveh T, Farb TB, et al. Thioredoxin-mimetic peptides (TXM) inhibit inflammatory pathways associated with high-glucose and oxidative stress. Free Radic Biol Med [Internet]. 2016;99:557–71. Available from: http://dx.doi.org/10.1016/j.freeradbiomed.2016.09.011

383. Vikramadithyan RK, Hiriyan J, Suresh J, Gershome C, Babu RK, Misra P, et al. DRF 2655: a unique molecule that reduces body weight and ameliorates metabolic abnormalities. Obes Res. 2003;11(2):293–303.

384. Chakrabarti R, Vikramadithyan RK, Prem Kumar M, Kumar SKB, Mamidi NVS, Misra P, et al. PMT13, a pyrimidone analogue of thiazolidinedione improves insulin resistance-associated disorders in animal models of type 2 diabetes. Diabetes, Obes Metab. 2002;4(5):319–28.

385. Hori H, Sasaoka T, Ishihara H, Wada T, Murakami S, Ishiki M, et al. Association of SH2-Containing Inositol Phosphatase2 With the Insulin Resistance of Diabetic db/db Mice. Diabetes. 2002;51(18):2387–94.

386. Madhavan GR, Chakrabarti R, Vikramadithyan RK, Mamidi RNVS, Balraju V, Rajesh BM, et al. Synthesis and biological activity of novel pyrimidinone containing thiazolidinedione derivatives. Bioorganic Med Chem. 2002;10(8):2671–80.

387. Gerhold DL, Liu F, Jiang G, Li Z, Xu J, Lu M, et al. Gene expression profile of adipocyte differentiation and its regulation by peroxisome proliferator-activated receptor-gamma agonists. Endocrinology [Internet]. 2002;143(6):2106–18. Available from: d:%5CAkh Literatur%5C00641.PDF

388. Etgen GJ, Oldham BA, Johnson WT, Broderick CL, Montrose CR, Brozinick JT, et al. A tailored therapy for the metabolic syndrome: The dual peroxisome proliferator-activated receptor-alpha/gamma agonist LY465608 ameliorates insulin resistance and diabetic hyperglycemia while improving cardiovascular risk factors in preclinical models. Diabetes. 2002;51(4):1083–7.

389. Clapham JC, Coulthard VH, Moore GB. Concordant mRNA expression of UCP-3, but not UCP-2, with mitochondrial thioesterase-1 in brown adipose tissue and skeletal muscle in db/db diabetic mice. Biochem Biophys Res Commun. 2001;287(5):1058–62.

390. Moore GB, Chapman H, Holder JC, Lister CA, Piercy V, Smith SA, et al. Differential regulation of adipocytokine mRNAs by rosiglitazone in db/db mice. Biochem Biophys Res Commun [Internet]. 2001;286(4):735–41. Available from: http://www.sciencedirect.com/science/article/pii/S0006291X01954609

391. Lohray BB, Lohray VB, Bajji AC, Kalchar S, Poondra RR, Padakanti S, et al. (-)3-[4-[2-(phenoxazin-10-yl)ethoxy]phenyl]-2-ethoxypropanoic acid [(-)DRF 2725]: A dual PPAR agonist with potent antihyperglycemic and lipid modulating activity. J Med Chem. 2001;44(16):2675–8.

392. Vikramadithyan RK, Chakrabarti R, Misra P, Premkumar M, Kumar SKB, Rao CS, et al. Euglycemic and hypolipidemic activity of PAT5A: A unique thiazolidinedione with weak peroxisome proliferator activated receptor gamma activity. Metabolism. 2000;49(11):1417–24.

393. Choi SI, Lee HA, Han JS. Gynura procumbens extract improves insulin sensitivity and suppresses hepatic gluconeogenesis in C57BL/KsJ-db/db mice. Nutr Res Pract. 2016;10(5):507–15.

394. Chaput E, Saladin R, Silvestre M, Edgar AD. Fenofibrate and rosiglitazone lower serum triglycerides with opposing effects on body weight. Biochem Biophys Res Commun [Internet]. 2000;271(2):445–50. Available from: http://www.sciencedirect.com/science/article/pii/S0006291X00926470

395. Lohray BB, Bhushan V, Reddy AS, Rao PB, Reddy NJ, Harikishore P, et al. Novel euglycemic and hypolipidemic agents. 4. Pyridyl- and quinolinyl- containing thiazolidinediones. J Med Chem. 1999;42(14):2569–81.

396. Burris TP, Pelton PD, Zhou L, Osborne MC, Cryan E, Demarest KT. A novel method for analysis of nuclear receptor function at natural promoters: peroxisome proliferator-activated receptor gamma agonist actions on aP2 gene expression detected using branched DNA messenger RNA quantitation. Mol Endocrinol. 1999;13(3):410–7.

397. Lohray BB, Bhushan V, Rao BP, Madhavan GR, Murali N, Rao KN, et al. Novel euglycemic and hypolipidemic agents. J Med Chem. 1998;41(10):1619–30.

398. Connor SC, Hughes MG, Moore G, Lister CA, Smith SA. Antidiabetic efficacy of BRL 49653, a potent orally active insulin sensitizing agent, assessed in the C57BL/KsJ db/db diabetic mouse by non-invasive 1H NMR studies of urine. J Pharm Pharmacol. 1997;49(3):336–44.

399. Yoshida S, Ohishi T, Matsui T, Tanaka H, Oshima H, Yonetoku Y, et al. Novel GPR119 agonist AS1535907 contributes to first-phase insulin secretion in rat perfused pancreas and diabetic db/db mice. Biochem Biophys Res Commun [Internet]. 2010;402(2):280–5. Available from: http://dx.doi.org/10.1016/j.bbrc.2010.10.015

400. Carroll MJ, Lister CA, Sennitt M V., Stewart-Long N, Cawthorne MA. Improved glycemic control in C57Bl/KsJ (db/db) mice after treatment with the thermogenic β-adrenoceptor agonist, BRL 26830. Diabetes. 1985;34(11):1198–204.

401. Chakrabarti R, Damarla RKB, Mullangi R, Sharma VM, Vikramadithyan RK, Rajagopalan R. Insulin sensitizing property of Indigofera mysorensis extract. J Ethnopharmacol. 2006;105(1–2):102–6.

402. Han X, Deng Y, Yu J, Sun Y, Ren G, Cai J, et al. Acarbose Accelerates Wound Healing via Akt/eNOS Signaling in db/db Mice. Oxid Med Cell Longev. 2017;2017:1–11.

403. Lee KH, Ha KS, Jo SH, Lee CM, Kim YC, Chung KH, et al. Effect of long-term dietary Arginyl-Fructose (AF) on hyperglycemia and HbA1c in diabetic db/db mice. Int J Mol Sci. 2014;15(5):8352–9.

404. Kim J-G, Jo S-H, Ha K-S, Kim S-C, Kim Y-C, Apostolidis E, et al. Effect of long-term supplementation of low molecular weight chitosan oligosaccharide (GO2KA1) on fasting blood glucose and HbA1c in db/db mice model and elucidation of mechanism of action. BMC Complement Altern Med [Internet]. 2014;14(1):272. Available from: http://www.pubmedcentral.nih.gov/articlerender.fcgi?artid=4124164&tool=pmcentrez&rendertype=abstract

405. Kim J-H, Kang M-J, Choi H-N, Jeong S-M, Lee Y-M, Kim J-I. Quercetin attenuates fasting and postprandial hyperglycemia in animal models of diabetes mellitus. Nutr Res Pract [Internet]. 2011;5(2):107–11. Available from: http://www.ncbi.nlm.nih.gov/pubmed/21556223%5Cnhttp://www.pubmedcentral.nih.gov/articlerender.fcgi?artid=PMC3085798

406. Kang MJ, Kim JH, Choi HN, Kim MJ, Han JH, Lee JH, et al. Hypoglycemic effects of Welsh onion in an animal model of diabetes mellitus. Nutr Res Pract. 2010;4(6):486–91.

407. Lee SM. The effect of chronic alpha-glycosidase inhibition on diabetic nephropathy in the db/db mouse. Diabetes [Internet]. 1982;31(3):249–54. Available from: http://www.ncbi.nlm.nih.gov/pubmed/6759241

408. Chang MS, Oh MS, Kim DR, Jung KJ, Park S, Choi SB, et al. Effects of Okchun-San, a herbal formulation, on blood glucose levels and body weight in a model of Type 2 diabetes. J Ethnopharmacol. 2006;103(3):491–5.
