# Supplementary material for: A standardised framework to identify optimal animal models for efficacy assessment in drug development
Source: PLoS One. 2019 Jun 13;14(6):e0218014. doi: 10.1371/journal.pone.0218014 (PMC6563989; doi:10.1371/journal.pone.0218014)
Supplement: S5 Supporting Information — (DOCX) [file pone.0218014.s005.docx]

# S5 Supporting Information – Validation Duchenne Muscular Dystrophy (DMD)

A total of 58 articles were included for the mdx mouse and 41 for the GRMD dog. The final scores were 67.78 and 65.42 for the mdx mouse and GRMD dog, respectively. The relative scores per parameter are presented in Fig A. The GRMD dog scores better in the epidemiological, SNH and histological domains while the mdx mouse does so in the pharmacological and endpoints domains. The GRMD dog mimics the natural history of the disease, symptoms (e.g. muscle wasting) and histopathological features (e.g. muscle regeneration) better than the mdx mouse. Especially for drugs which aim to slow down muscle degeneration or delay the disease onset, the GRMD dog is likely to generate more translatable data than the mdx mouse.

**Fig A. DMD models results.** Radar plot with the scores per parameter per model of DMD. The closer a parameter is to the edge, the better the model simulates that aspect of the human disease.

The difference in the pharmacological and endpoints domains stems mostly from the uncertainty factor, which is 18.2% and 6.1% for the GRMD dog and the mdx mouse respectively. Since most drug screening studies are done in mdx mice, there are more studies available for the pharmacological validation. However, there are no published studies in GRMD dogs for most drugs tested in humans. The only published study that assessed a functional outcome did so in sedated dogs, which reduced the score of the Endpoints Validation. Hence, a comparison between these models in these two domains is unlikely to be informative.

A reporting quality and risk of bias assessment was included based on parameters adapted from the ARRIVE guidelines (see S2 Supporting Information, section F). Publications with experiments on two models were counted separately for each model. A total of 35 preclinical studies were included for the mdx mouse (32) and GRMD dog (3). Assessments of reporting quality and risk of bias per publication for the two models are provided in S7 Supporting Information. Table A shows the aggregated data for reporting quality and Table B for the risk of bias.

Most preclinical studies did not report important measures to reduce the risk of bias, such as any mention of randomisation (68.6%) and blinding (57.1%). Even studies which did add these measures to the design did not include information detailed enough on how the blinding and randomisation were executed to allow a proper evaluation of the risk of selection, performance and detection biases. The selective outcome reporting was the only parameter in which most studies were graded with a low risk of bias, indicating all outcomes defined in the methodology were reported in the results. These findings are in line with previous literature, which puts the reliability of these experiments into question [1–5].

Male animals were more than two times more likely to be used than females while studies including both sexes accounted for almost one-third of the total (31.8%). Although in DMD this does not have a significant impact due to most patients with DMD being males, this trend is consistent with our findings for T2D which shown an overrepresentation of males in non-human animal studies (see S4 and S8 Supporting Information files).

Table A: Percentage of studies complying with the reporting quality parameters adapted from the ARRIVE guidelines per parameter per model.

| Parameter | Mdx Mouse  (N =32) | GRMD dog  (N = 3) | Total  (N = 35) | Parameter | Mdx Mouse  (N =32) | GRMD dog  (N = 3) | Total  (N = 35) |
| --- | --- | --- | --- | --- | --- | --- | --- |
|  | Y (%) | | |  | Y (%) | | |
| Type of Facility | 6.3 | 0.0 | 5.7 | Environmental Enrichment | 0.0 | 0.0 | 0.0 |
| Type of Cage or Housing | 12.5 | 0.0 | 11.4 | Any Blinding | 43.8 | 33.3 | 42.9 |
| Bedding Material | 0.0 | 0.0 | 0.0 | Any Randomisation | 34.4 | 0.0 | 31.4 |
| Number of Cage Companions | 12.5 | 0.0 | 11.4 | Sample Size | 84.4 | 100.0 | 85.7 |
| Breeding Programme | 53.1 | 100.0 | 57.1 | Sample Size Calculation | 6.3 | 33.3 | 8.6 |
| Light/Dark Cycle | 25.0 | 0.0 | 22.9 | Acclimatisation | 18.8 | 0.0 | 17.1 |
| Temperature and Humidity | 3.1 | 0.0 | 2.9 | Sex Disclosed | 46.9 | 33.3 | 45.7 |
| Quality of the Water (fish) | - | - | - | Male/Female/Both  (N = 15/1/16 ) | 53.3/20.0/26.7 | 0/0/100 | 50.0/18.8/31.3 |
| Type of Food | 6.3 | 0.0 | 5.7 | Background Control | - | - | - |
| Access to Food and Water | 34.4 | 0.0 | 31.4 | Background Model | - | - | - |

Table B: Percentage of studies with low (Y) and unclear (U) risk of bias per parameter per model.

| Risk of Bias | Mdx Mouse  (N =32) | GRMD dog  (N = 3) | Total  (N = 35) |
| --- | --- | --- | --- |
|  | Yes/Unclear (%) | | |
| Allocation Concealment | 0/100 | 0/100 | 0/100 |
| Blinded Outcome Assessment | 3.1/96.8 | 0/100 | 2.9/97.1 |
| Blinded Operations | 0/100 | 0/100 | 0/100 |
| Random Cage Allocation | 0/100 | 0/100 | 0/100 |
| Random Outcome Assessment | 0/100 | 0/100 | 0/100 |
| Sequence Generation | 0/96.8 | 0/100 | 0/97.1 |
| Baseline Characteristics | 3.1/96.8 | 0/100 | 2.9/97.1 |
| Incomplete Outcome Data | 18.8/67.7 | 0/100 | 17.1/71.4 |
| Selective Outcome Reporting | 96.9/3.2 | 100/0 | 97.1/2.9 |
| Other | 71.9/6.5 | 33.3/0.0 | 68.6/5.7 |

| **MODEL NAME** | C57Bl10scsn-Dmdmdx mouse (mdx mouse) |
| --- | --- |
| **INDICATION** | Duchenne Muscular Dystrophy |
| **VALIDATION DATE** | 19.09.2016 |
| **TOTAL SUBSECTIONS** | 33 |
| **TOTAL SCORE** | 67.78 |
| **UNCERTAINTY FACTOR (%)** | 6.06 |
| **VALIDATION LEVEL (%)** | Highly Validated (93.9) |
| **HISTORICAL BACKGROUND** | |
| The muscular dystrophy X-chromosome linked (mdx) mouse is the most widely used animal model to study Duchenne Muscular Dystrophy (DMD) [6]. It was first described by Bulfield et al in the 80’s, after inbreeding C57BL/10ScSn mice for five generations and noticing increased creatine kinase (CK) and pyruvate kinase (PK). Histopathological muscle damage similar to DMD patients was also observed [7]. Later this spontaneous mutation was characterised by Sicinski et al as being a nonsense point mutation (C-to-T transition) in exon 23, which leads to failure in expressing full-length dystrophin [8]. | |

| **1. EPIDEMIOLOGICAL VALIDATION** | |
| --- | --- |
| 1.1 Is the model able to simulate the disease in the relevant sexes? | Score |
| Yes, completely.  Remarks: most literature published on mdx mice reports the use of male animals. DMD occurs almost exclusively in males with an incidence of 1 every 3,500 to 6,291 births [9,10]. | 6.25 |
| 1.2 Is the model able to simulate the disease in the relevant age groups (juvenile, adult or ageing)? | Score |
| Yes, partially.  Remarks: DMD is a disease caused by a genetic defect in the dystrophin coding gene. Humans are affected during infancy, with clinical presentation at around 2.5 years. Mdx mice present pathological signs at 3 weeks, but clinical symptoms appear only in late adulthood (12 to 15 months). Hence, there is only a limited period the disease overlaps in both species [11–16]. | 3.13 |

| **2. SYMPTOMATOLOGY AND NATURAL HISTORY (SNH) VALIDATION** | |
| --- | --- |
| 2.1 Is the model able to replicate the symptoms and co-morbidities commonly present in this disease? If so, which ones? | Score |
| Yes, partially. | 1.61 |
| 2.1.1 Symptoms modelled |  |
| Reduced grown-up body weight (BW): BW is reduced in mdx mice when compared to control or wild-type mice [14,17]. |  |
| ECG abnormality: similar to humans, ECG changes such as sinus tachycardia, shortened PR interval, prolonged QT interval, deep Q wave, and polyphasic R’ wave are observed in old mdx mice [14]. |  |
| Cardiomyopathy: fibrosis, calcification and enlarged ventricular chamber are also present in mdx mice [7,14]. |  |
| 2.1.2 Symptoms partially modelled |  |
| Reduced lifespan: approximately 18% reduced lifespan when compared to wild-type mice [18]. In humans, the reduction is more significant at around 75% [6,13]. |  |
| Muscle wasting: it is comparable to DMD boys only regarding diaphragm degeneration [7,18,19]. |  |
| Cognitive and CNS defects: mdx mice show a deficit in cognitive deficit flexibility, long-term spatial and recognition memory, although somewhat milder than in boys in with DMD and with no short-term memory involvement [20–26]. |  |
| 2.1.3 Symptoms not modelled |  |
| Loss of ambulation: hardly ever present in mdx mice while for humans it happens between 10 and 15 years – the latter being for patients with less damaging exon skipping and treatment with glucocorticoids [6,12,13,17]. |  |
| 2.2 Is the natural history of the disease similar to humans regarding: |  |
| 2.2.1 Time to onset; | Score |
| No.  Remarks: although histological marks of disease can be detected early (at 3 weeks is the start of limb muscle degeneration), real impairment (muscle wasting, scoliosis and heart failure) do not occur until mice are 15 months or older – equivalent to around 26 years in humans [7,14–17,27–29]. In humans, first symptoms start around 2.5 years, with loss of ambulation around 10 to 15 years [11–13]. | 0 |
| 2.2.2 Disease progression; | Score |
| Yes, partially.  Remarks: mild progression, as most of the limb muscles maintain hypertrophy although with a loss in specific force and normalised power unlike DMD boys, whose muscles’ degenerative processes largely outdistance the regenerative, rapidly causing muscle atrophy [6,18,30]. Although histological marks of disease can be detected early (at 3 weeks is the start of limb muscle degeneration; at 5 weeks is the start of muscle necrosis; at 9 weeks (adult mice): all muscles are affected), real impairment (muscle wasting, scoliosis and heart failure) do not occur until mice are 15 months or older [7,14–17,27,28]. At ~12 to 15 months old mdx mice show abnormal histopathology, echocardiography and ECDG and at 21 months old (eq. to ~53 years in humans) mdx mice show signs of cardiomyopathy [14]. In humans, first symptoms start around 2.5 years, with loss of ambulation around 10 to 15 years [11–13]. | 1.25 |
| 2.2.3 Duration of symptoms; | Score |
| Yes, completely.  Remarks: symptoms in DMD are lifelong due to permanent muscle degeneration. In mdx mice, once symptoms get worse, there is also permanent damage that leads to reduced lifespan [18]. | 2.50 |
| 2.2.4 Severity. | Score |
| Yes, partially.  Remarks: mdx mice do not show the same level of muscle degeneration as seen in humans, except for the diaphragm which shows progressive degeneration evident at 6 months of age with great variation in myofibre size, architecture and continuous necrosis and proliferation of connective tissue [7,18,19,30]. | 1.25 |

| **3. GENETIC VALIDATION** | |
| --- | --- |
| 3.1 Does this species also have orthologous genes and/or proteins involved in the human disease? | Score |
| Yes.  Remarks: DMD is caused by the lack of dystrophin, thus the dystrophin gene alongside with utrophin (a functional and structural analogue) have been included in this section [31]. The mouse has orthologous genes for both of them [32,33]. | 4.17 |
| 3.1.1 Dystrophin |  |
| The human dystrophin gene (Gene ID: 1756) is the largest gene found in humans measuring a total of 2.4 Mb located at Xp21.2-p21.1 [34]. Dystrophin forms parts of the dystrophin-glycoprotein complex (DGC), responsible for connecting the inner cytoskeleton and the extracellular matrix [34]. In mice, the dystrophin gene (Gene ID: 13405) is located at chromosome X C1; X 38.38 cM [32]. |  |

| 3.1.2 Utrophin |  |
| --- | --- |
| The human utrophin gene (Gene ID: 7402) is located in humans at 6q24.2 [35]. Utrophin is a structural and functional analogue to dystrophin, being also present at neuromuscular synapses and myotendinous junctions [35]. Utrophin upregulation is thought to be a compensation mechanism partially responsible for the milder phenotype in mdx mice [35]. In mice, the utrophin gene (Gene ID: 22288) is located at chromosome 10 A1-A2; 10 3.77 cM [33]. |  |
| 3.2 If so, are the relevant genetic mutations or alterations also present in the orthologous genes/proteins? | Score |
| Yes, partially.  Remarks: DMD is caused by deletions, duplications, small mutations or other smaller rearrangements of the gene that codes for dystrophin in chromosome X [31]. In that sense, DMD being an X-linked inherited disorder, the mdx mouse has the same aetiology (spontaneous mutations). Nevertheless, mdx mice have a nonsense point mutation (C-to-T transition) in exon 23 that aborts full-length dystrophin expression [8]. This is not the case for human patients who have other alterations that lead to dystrophin deficiency. | 2.09 |
| 3.3 If so, is the expression of such orthologous genes and/or proteins similar to the human condition? | Score |
| Yes, completely. | 4.16 |
| 3.3.1 Dystrophin |  |
| Yes, completely.  Remarks: like DMD patients, mdx mice are dystrophin-deficient, not expressing full-length dystrophin [8]. |  |
| 3.3.2 Utrophin |  |
| Yes, completely.  Remarks: utrophin upregulation (a compensation mechanism partially responsible for the milder phenotype in mdx mice) is reported in mdx mice and humans [14,36,37]. |  |

| **4. BIOCHEMICAL VALIDATION** | |
| --- | --- |
| 4.1 If there are known pharmacodynamic (PD) biomarkers related to the pathophysiology of the disease, are they also present in the model? | Score |
| Yes, completely.  Remarks: high levels of creatine kinase (CK) – a marker of muscle damage, with the muscle-type variants being predominant in plasma are present in mdx mice [7,38]. Pyruvate kinase (PK) is also increased, although it has secondary importance and it is a less reliable marker of muscle damage than CK, being included here only for information purposes [38]. | 3.13 |
| 4.2 Do these PD biomarkers behave similarly to humans’? | Score |
| Yes, completely.  Remarks: both PK and CK are increased in mdx mice and humans [7,38]. | 3.13 |
| 4.3 If there are known prognostic biomarkers related to the pathophysiology of the disease, are they also present in the model? | Score |
| N/A.  Remarks: currently, DMD has no prognostic biomarker that is validated and widely used in clinical practice. Many biomarkers are still under exploratory phase. A list of biomarkers being explored can be found in the supplementary material from Guiraud et al, 2015 and in the paper from Hathout et al, 2016 [39,40]. | - |
| 4.4 Do these prognostic biomarkers behave similarly to humans’? | Score |
| N/A. | - |

| **5. AETIOLOGICAL VALIDATION** | |
| --- | --- |
| 5.1 Is the aetiology of the disease similar to humans’? | Score |
| Yes, partially.  Remarks: DMD is caused by deletions, duplications, small mutations or other smaller rearrangements of the gene that codes for dystrophin in chromosome X [31]. In that sense, DMD being an X-linked inherited disorder, the mdx mouse has the same aetiology (spontaneous mutations). Nevertheless, mdx mice have a nonsense point mutation (C-to-T transition) in exon 23 that aborts full-length dystrophin expression [8]. This is not the case for human patients who have other alterations that lead to dystrophin deficiency. | 6.25 |

| **6. HISTOLOGICAL VALIDATION** | |
| --- | --- |
| 6.1 Do the histopathological structures in relevant tissues resemble the ones found in humans? | Score |
| Yes, partially. | 4.17 |
| 6.1.1 Histopathological features modelled |  |
| Limb muscle fibrosis: like in DMD boys, it is significantly increased in the mdx mouse muscles [16,17,27]. |  |
| 6.1.2 Histopathological features not modelled |  |
| Muscle regeneration: in mdx mice, muscle regeneration is significantly higher than in humans until old age [17,27,28]. |  |
| Adipose tissue: scarcely developed in mdx muscle, unlike in DMD patients [17,28]. |  |
| Remarks: acute necrosis is present in mdx mice muscles but not in humans [17]. | - |

| **7. PHARMACOLOGICAL VALIDATION** | | | | | | |
| --- | --- | --- | --- | --- | --- | --- |
| 7.1 Are effective drugs in humans also effective in this model? | | | | | Score | |
|  | | | | | 3.61 | |
| 7.1.1 Glucocorticosteroids | | | | |  | |
| 7.1.1.1 Prednisone [41–55] | | | | |  | |
| Methodology: studies were searched on 13/09/2016 on PubMed with the string ‘mdx AND (Prednisone[mesh] OR prednisone OR Apo-Prednisone OR Cortan OR Cortancyl OR Cutason OR Dacortin OR Decortin OR Decortisyl OR Dehydrocortisone OR Deltasone OR Encorton OR Encortone OR Enkortolon OR Kortancyl OR “Liquid Pred” OR Meticorten OR Orasone OR Panafcort OR Panasol OR Predni Tablinen OR Prednidib OR Predniment OR Prednison Acsis OR “Prednison Galen” OR “Prednison Hexal” OR Pronisone OR Rectodelt OR Sone OR Sterapred OR Ultracorten OR Winpred OR delta-Cortisone)’, yielding 27 results; and on Embase with the string ‘("Duchenne muscular dystrophic mouse" OR "Duchenne muscular dystrophy mouse" OR "MDX mouse" OR "mice, inbred mdx" OR "X chromosome-linked muscular dystrophy mouse" OR "X-linked muscular dystrophic mouse" OR "X-linked muscular dystrophy mouse") AND (prednisone OR “1, 2 dehydrocortisone” OR “17, 21 dihydroxypregna 1, 4 diene 3, 11, 20 trione” OR ancortone OR apo-prednisone OR biocortone OR colisone OR cortan OR cortidelt OR cortiprex OR cutason OR dacorten OR "de cortisyl" OR decortancyl OR decortin OR "decortin e merck" OR decortine OR decortisyl OR dehydrocortisone OR dekortin OR delitisone OR "dellacort a" OR “delta 1 dehydrocortisone” OR “delta cortelan” OR “delta cortisone” OR “delta dome” OR “delta e” OR “delta prenovis” OR delta-dome OR deltacorten OR deltacortene OR deltacortisone OR deltacortone OR deltasone OR deltison OR deltisona OR deltra OR "di adreson" OR di-adreson OR diadreson OR drazone OR encorton OR encortone OR enkorton OR fernisone OR hostacortin OR insone OR “liquid pred” OR lodotra OR me-korti OR meprison OR metacortandracin OR meticorten OR meticortine OR nisona OR “nsc 10023” OR nsc10023 OR orasone OR orisane OR panafcort OR paracort OR pehacort OR precort OR precortal OR prednicen-m OR prednicorm OR prednicot OR prednidib OR prednison OR “prednisone alcohol” OR “prednisone intensol” OR “prednisone test” OR prednitone OR “pregna 1, 4 diene 3, 11, 20 trione 17, 21 diol” OR pronison OR pronisone OR pronizone OR pulmison OR rayos OR rectodelt OR servisone OR steerometz OR sterapred OR "sterapred ds" OR ultracorten OR urtilone OR winpred), yielding 34 results; which were screened by title and abstract. Of these, fifteen (15) articles were included in this section.  Results: a significant part of the studies shows a positive effect prednisone in histological (e.g. reduction of the percentage of fibres with centrally located nuclei, inflammatory cells infiltration etc.) and functional outcomes (e.g. fore- and hindlimb grip strengths, respiratory function and motor coordination), although in some studies prednisone did not show any effects on functional outcomes (e.g. total distance in open field tests, fore- and hindlimb grip strengths). However, there are also reports of an increase in heart muscle fibrosis. Seven (7) articles were classified as category I and eight (8) were classified as category II. Of the latter, six (6) studies are in line with clinical findings. | | | | |  | |
| Reporting Quality | | | | Risk of Bias | | |
| Parameter (N = 15) | Y (%) |  | Y (%) | Parameter (N = 15) | | Y/U (%) |
| Type of Facility | 13.3 | Environmental Enrichment | 0.0 | Allocation Concealment | | 0/100 |
| Type of Cage or Housing | 20.0 | Any Blinding | 66.7 | Blinded Outcome Assessment | | 6.7/93.3 |
| Bedding Material | 0.0 | Any Randomisation | 40.0 | Blinded Operations | | 0/100 |
| N Cage Companions | 20.0 | Sample Size | 100 | Random Cage Allocation | | 0/100 |
| Breeding Programme | 53.3 | Sample Size Calculation | 13.3 | Random Outcome Assessment | | 0/100 |
| Light/Dark Cycle | 46.7 | Acclimatisation | 33.3 | Sequence Generation | | 0/93.3 |
| Temperature and Humidity | 0.0 | Sex Disclosed | 53.3 | Baseline Characteristics | | 6.7/93.3 |
| Quality of the Water (fish) | - | Male/Female/Both  (N = 8) | 62.5/25.0/12.5 | Incomplete Outcome Data | | 13.3/60.0 |
| Type of Food | 6.7 | Background Control | - | Selective Outcome Reporting | | 100/0 |
| Access to Food and Water | 53.3 | Background Model | - | Other | | 66.7/0 |

| 7.1.1.2 Deflazacort [45,46,48,49,56–63] | | | | | |  |
| --- | --- | --- | --- | --- | --- | --- |
| Methodology: studies were searched on 13/09/2016 on PubMed with the string ‘mdx AND deflazacort’, yielding 15 results; and on Embase with the string ‘("Duchenne muscular dystrophic mouse" OR "Duchenne muscular dystrophy mouse" OR "MDX mouse" OR "mice, inbred mdx" OR "X chromosome-linked muscular dystrophy mouse" OR "X-linked muscular dystrophic mouse" OR "X-linked muscular dystrophy mouse") AND deflazacort OR “9 defluorofluazacort” OR azacort OR calcort OR deflan OR defluorofluazacort OR “dl 458” OR “dl 458 it” OR “dl 458it” OR “dl 5458” OR dl458 OR dl458it OR dl5458 OR emflaza OR flantadin OR “fluazacort, difluoro” OR rosilar, yielding 25 results; which were screened by title and abstract. Of these, twelve (12) articles were included in this section.  Results: all animal studies show positive effects on histological outcomes (reduction of the percentage of fibres with centrally located nuclei, on the prevalence of dystrophic lesions, fibre size variability, the density of inflammatory cells and area). Of the few which investigated functional outcomes (e.g. increase in total run distance and hindlimb grip strengths and reduction in muscle fatigue), all demonstrated benefits on muscle function although no effect was reported on exploratory behaviour. Nine (9) articles were classified as category I and three (3) were classified as category II. Of the latter, all studies are in line with clinical findings. | | | | | |  |
| Reporting Quality | | | | Risk of Bias | | |
| Parameter (N = 12) | Y (%) |  | Y (%) | Parameter (N = 12) | Y/U (%) | |
| Type of Facility | 0.0 | Environmental Enrichment | 0.0 | Allocation Concealment | 0/100 | |
| Type of Cage or Housing | 0.0 | Any Blinding | 58.3 | Blinded Outcome Assessment | 0/100 | |
| Bedding Material | 0.0 | Any Randomisation | 41.7 | Blinded Operations | 0/100 | |
| N Cage Companions | 0.0 | Sample Size | 91.7 | Random Cage Allocation | 0/100 | |
| Breeding Programme | 41.7 | Sample Size Calculation | 0.0 | Random Outcome Assessment | 0/100 | |
| Light/Dark Cycle | 0.0 | Acclimatisation | 0.0 | Sequence Generation | 0/100 | |
| Temperature and Humidity | 0.0 | Sex Disclosed | 25.0 | Baseline Characteristics | 0/100 | |
| Quality of the Water (fish) | - | Male/Female/Both  (N = 3) | 33.3/0/66.7 | Incomplete Outcome Data | 33.3/58.3 | |
| Type of Food | 0.0 | Background Control | - | Selective Outcome Reporting | 100/0 | |
| Access to Food and Water | 8.3 | Background Model | - | Other | 83.3/0 | |

| 7.1.2 Read-through compound: Ataluren [64] | | | | | |  |
| --- | --- | --- | --- | --- | --- | --- |
| Methodology: studies were searched on 13/09/2016 on PubMed with the string ‘mdx AND (ptc124 OR ataluren)’, yielding 7 results; and on Embase with the string ‘(“duchenne muscular dystrophic mouse” OR “duchenne muscular dystrophy mouse” OR “mdx mouse” OR “mice, inbred mdx” OR “x chromosome-linked muscular dystrophy mouse” OR “x-linked muscular dystrophic mouse” OR “x-linked muscular dystrophy mouse”) AND ("3 [5 (2 fluorophenyl) 1, 2, 4 oxadiazol 3 yl] benzoic acid" OR "3 [5 (2 fluorophenyl) [1, 2, 4] oxadiazol 3 yl] benzoic acid" OR "ataluren sodium" OR "ptc 124" OR ptc124 OR translarna)’, yielding 11 results; which were screened by title and abstract. Of these, one (1) article was included in this section.  Results: the only study show dystrophin detection in all muscles examined, including restoration of the dystrophin-glycoprotein complex (DCG), reduction of CK levels (linked to muscle damage), increase in average force and hanging time (4-limb hanging test) and reduction in functional strength deficit. This article was classified as category II. This study is in line with clinical findings. | | | | | |  |
| Reporting Quality | | | | Risk of Bias | | |
| Parameter (N = 1) | Y (%) |  | Y (%) | Parameter (N = 1) | Y/U (%) | |
| Type of Facility | 0.0 | Environmental Enrichment | 0.0 | Allocation Concealment | 0/100 | |
| Type of Cage or Housing | 0.0 | Any Blinding | 0.0 | Blinded Outcome Assessment | 0/100 | |
| Bedding Material | 0.0 | Any Randomisation | 100 | Blinded Operations | 0/100 | |
| N Cage Companions | 0.0 | Sample Size | 0.0 | Random Cage Allocation | 0/100 | |
| Breeding Programme | 100 | Sample Size Calculation | 0.0 | Random Outcome Assessment | 0/100 | |
| Light/Dark Cycle | 0.0 | Acclimatisation | 0.0 | Sequence Generation | 0/100 | |
| Temperature and Humidity | 0.0 | Sex Disclosed | 100 | Baseline Characteristics | 0/100 | |
| Quality of the Water (fish) | - | Male/Female/Both  (N = 1) | 100/0/0 | Incomplete Outcome Data | 0/100 | |
| Type of Food | 0.0 | Background Control | - | Selective Outcome Reporting | 0/100 | |
| Access to Food and Water | 0.0 | Background Model | - | Other | 100/0 | |

| 7.1.3 Exon skipping: Eteplirsen [65–69] | | | | | |  |
| --- | --- | --- | --- | --- | --- | --- |
| Methodology: As eteplirsen is designed to skip exon 51 in humans, it cannot itself be tested in mdx mice, which carry a mutation in exon 23. Thus, we also searched for AVI 4225, which is the mouse homologous compound of eteplirsen. Studies were searched on 19/09/2016 on PubMed with the string ‘mdx AND ‘(eteplirsen OR avi-4658 OR "avi 4658" OR AVI-4225 OR “AVI 4225”)’, yielding 2 results; and on Embase with the string ‘(“duchenne muscular dystrophic mouse” OR “duchenne muscular dystrophy mouse” OR “mdx mouse” OR “mice, inbred mdx” OR “x chromosome-linked muscular dystrophy mouse” OR “x-linked muscular dystrophic mouse” OR “x-linked muscular dystrophy mouse”) AND (eteplirsen OR “avi 4658” OR avi4658 OR “avi 4225” OR avi-4225 OR etiplersen OR etiplirsen OR exondys OR “exondys 51”)’, yielding 7 results; which were screened by title and abstract. Of these, one article was included in this section. Moreover, when looking at FDA’s Pharmacology Review of eteplirsen, four (4) studies are cited on the mdx mouse with eteplirsen’s mouse homologous compound AVI-4225 [70]. These four (4) studies were also included in this section, leading to a total of five (5) studies.  Results: in general, all studies showed increased dystrophin expression in muscle. Histological measures generally improved, including the diaphragm and cardiac function. Muscle strength was also reported to have improved in functional measures (e.g. grip or tetanic strength). All articles but one were classified as category II. All studies are in line with clinical findings. | | | | | |  |
| Reporting Quality | | | | Risk of Bias |  | |
| Parameter (N = 5) | Y (%) |  | Y (%) | Parameter (N = 5) | Y/U (%) | |
| Type of Facility | 0.0 | Environmental Enrichment | 0.0 | Allocation Concealment | 0/100 | |
| Type of Cage or Housing | 20.0 | Any Blinding | 0.0 | Blinded Outcome Assessment | 0/100 | |
| Bedding Material | 0.0 | Any Randomisation | 20.0 | Blinded Operations | 0/100 | |
| N Cage Companions | 20.0 | Sample Size | 80.0 | Random Cage Allocation | 0/100 | |
| Breeding Programme | 60.0 | Sample Size Calculation | 0.0 | Random Outcome Assessment | 0/100 | |
| Light/Dark Cycle | 20.0 | Acclimatisation | 0.0 | Sequence Generation | 0/100 | |
| Temperature and Humidity | 20.0 | Sex Disclosed | 25.0 | Baseline Characteristics | 0/100 | |
| Quality of the Water (fish) | - | Male/Female/Both  (N = 2) | 50/50/0 | Incomplete Outcome Data | 20.0/80.0 | |
| Type of Food | 0.0 | Background Control | - | Selective Outcome Reporting | 80.0/20.0 | |
| Access to Food and Water | 40.0 | Background Model | - | Other | 80.0/20.0 | |

| 7.2 Are ineffective drugs in humans also ineffective in this model? | | | | | | Score |
| --- | --- | --- | --- | --- | --- | --- |
| Two identified drug classes comprise drugs that were tested in humans and failed due to the lack of efficacy: aminopeptidase and leucine peptidase inhibitors; and myostatin inhibitors. | | | | | | 0.21 |
| 7.2.1 Aminopeptidase and leucine peptidase inhibitors: Bestatin [71] | | | | | |  |
| Methodology: studies were searched on 19/09/2016 on PubMed with the string ‘ mdx AND (Ubenimex[mesh] OR ubenimex OR “uben 3-amino-2-hydroxy-4-phenylbutyryl-L-leucine” OR bestatin OR “bestatin, (D-Leu)-(R-(R*,R*))-isomer” OR “bestatin, (D-Leu)-(R-(R*,S*))-isomer” OR “bestatin, (D-Leu)-(S-(R*,S*))-isomer” OR “bestatin, (L-Leu)-(R-(R*,R*))-isomer OR bestatin, (L-Leu)-(R-(R*,S*))-isomer” OR “bestatin, (L-Leu)-(S-(R*,R*))-isomer” OR “bestatin, (L-Leu)-(S-(R*,S*))-isomer, hydrochloride”)’, yielding 1 result; and on Embase with the string ‘(“duchenne muscular dystrophic mouse” OR “duchenne muscular dystrophy mouse” OR “mdx mouse” OR “mice, inbred mdx” OR “x chromosome-linked muscular dystrophy mouse” OR “x-linked muscular dystrophic mouse” OR “x-linked muscular dystrophy mouse”) AND (bestatin OR “(3 amino 2 hydroxy 4 phenylbutanoyl) leucine” OR “(3 amino 2 hydroxy 4 phenylbutyryl) leucine OR bestatine” OR “n (3 amino 2 hydroxy 1 oxo 4 phenylbutyl) leucine” OR “nk 421” OR ubenimex)’, yielding 11 results; which were screened by title and abstract. Of these, one (1) article was included in this section.  Results: this study found shows an improvement in resting membrane potentials (RMPs), which almost attained normal levels. Additionally, administration of bestatin significantly reduced the occurrence of electrical myotonia. This article was classified as category I. | | | | | |  |
| Reporting Quality | | | | Risk of Bias | | |
| Parameter (N = 1) | Y (%) |  | Y (%) | Parameter (N = 1) | Y/U (%) | |
| Type of Facility | 0.0 | Environmental Enrichment | 0.0 | Allocation Concealment | 0/100 | |
| Type of Cage or Housing | 0.0 | Any Blinding | 0.0 | Blinded Outcome Assessment | 0/100 | |
| Bedding Material | 0.0 | Any Randomisation | 0.0 | Blinded Operations | 0/100 | |
| N Cage Companions | 0.0 | Sample Size | 0.0 | Random Cage Allocation | 0/100 | |
| Breeding Programme | 0.0 | Sample Size Calculation | 0.0 | Random Outcome Assessment | 0/100 | |
| Light/Dark Cycle | 0.0 | Acclimatisation | 0.0 | Sequence Generation | 0/100 | |
| Temperature and Humidity | 0.0 | Sex Disclosed | 0.0 | Baseline Characteristics | 0/100 | |
| Quality of the Water (fish) | - | Male/Female/Both  (N = 1) | -/-/- | Incomplete Outcome Data | 0/100 | |
| Type of Food | 0.0 | Background Control | - | Selective Outcome Reporting | 100/0 | |
| Access to Food and Water | 0.0 | Background Model | - | Other | 0/100 | |

| 7.2.2 Myostatin inhibitors: Stamulumab [72] | | | | | |  |
| --- | --- | --- | --- | --- | --- | --- |
| Methodology: studies were searched on 19/09/2016 on PubMed with the string ‘mdx AND ("myostatin inhibitor" OR "myostatin inhibitors" OR "inhibition of myostatin" OR "myostatin blockade")’, yielding 33 results; and on Embase with the string ‘(“duchenne muscular dystrophic mouse” OR “duchenne muscular dystrophy mouse” OR “mdx mouse” OR “mice, inbred mdx” OR “x chromosome-linked muscular dystrophy mouse” OR “x-linked muscular dystrophic mouse” OR “x-linked muscular dystrophy mouse”) AND ("myostatin inhibitor" OR "myostatin inhibitors" OR "inhibition of myostatin" OR "myostatin blockade")’, yielding 11 results; which were screened by title and abstract. Of these, one (1) articles were included in this section.  Results: this study reported an increase in body weight, muscle mass, size and absolute muscle strength as assessed by the Rota-rod apparat. Moreover, stamulumab decreased muscle degeneration and serum CK levels. This article was classified as category I. | | | | | |  |
| Reporting Quality | | | | Risk of Bias | | |
| Parameter (N = 1) | Y (%) |  | Y (%) | Parameter (N = 1) | Y/U (%) | |
| Type of Facility | 0.0 | Environmental Enrichment | 0.0 | Allocation Concealment | 0/100 | |
| Type of Cage or Housing | 0.0 | Any Blinding | 0.0 | Blinded Outcome Assessment | 0/100 | |
| Bedding Material | 0.0 | Any Randomisation | 0.0 | Blinded Operations | 0/100 | |
| N Cage Companions | 0.0 | Sample Size | 0.0 | Random Cage Allocation | 0/100 | |
| Breeding Programme | 0.0 | Sample Size Calculation | 0.0 | Random Outcome Assessment | 0/100 | |
| Light/Dark Cycle | 0.0 | Acclimatisation | 100 | Sequence Generation | 0/100 | |
| Temperature and Humidity | 0.0 | Sex Disclosed | 100 | Baseline Characteristics | 0/100 | |
| Quality of the Water (fish) | - | Male/Female/Both  (N = 1) | 100/0/0 | Incomplete Outcome Data | 0/100 | |
| Type of Food | 0.0 | Background Control | - | Selective Outcome Reporting | 100/0 | |
| Access to Food and Water | 0.0 | Background Model | - | Other | 100/0 | |

| 7.3 Have drugs with different mechanisms of action and acting on different pathways been tested in this model? If so, which? | Score |
| --- | --- |
| Yes, partially.  Remarks: All the 5 identified mechanisms of action that have been tested in humans have also been tested in mdx mice. | 4.16 |

| **8. ENDPOINT VALIDATION** | |
| --- | --- |
| 8.1 Are the endpoints used in preclinical studies the same or translatable to the clinical endpoints? | Score |
| Yes.  Remarks: most functional measurements of functional pathology in mice, such as grip strength and treadmill exercise are directly correlated to the functional measurements in humans (6-min walking test, ability to stand up without the use of hands etc.). | 6.25 |
| 8.2 Are the methods used to assess preclinical endpoints comparable to the ones used to assess related clinical endpoints? | Score |
| Yes.  Remarks: See question 8.1. | 6.25 |

| **MODEL NAME** | Golden Retriever Muscular Dystrophy (GRMD) dog |
| --- | --- |
| **INDICATION** | Duchenne Muscular Dystrophy |
| **VALIDATION DATE** | 19.09.2016 |
| **TOTAL SUBSECTIONS** | 33 |
| **TOTAL SCORE** | 65.42 |
| **UNCERTAINTY FACTOR (%)** | 18.2 |
| **VALIDATION LEVEL (%)** | Highly Validated (81.8) |
| **HISTORICAL BACKGROUND** | |
| The Golden Retriever Muscular Dystrophy (GRMD) dog was first identified in 1958, but it only started to be thoroughly characterised in the 80’s [73,74]. The mutation in the dog dystrophin gene appeared spontaneously and it was later characterised by Sharp et al, consisting of a point mutation in exon 6, which causes the skipping of exon 7 further leading to the expression of the truncated and non-functional form of dystrophin [75]. Colonies are maintained mostly in academic centres the USA, France, Brazil and Australia [6].  A substantial variation in disease severity, such as the lifespan and ambulation ability of different colonies has been reported in the literature [76]. This should be considered when interpreting the data hereby presented. | |

| **1. EPIDEMIOLOGICAL VALIDATION** | |
| --- | --- |
| 1.1 Is the model able to simulate the disease in the relevant sexes? | Score |
| Yes, completely  Remarks: most literature published on GRMD dogs reports the use of male animals. DMD occurs almost exclusively in males with an incidence of 1 every 3,500 to 6,291 births [9,10] | 6.25 |
| 1.2 Is the model able to simulate the disease in the relevant age groups (juvenile, adult or ageing)? | Score |
| Yes, completely  Remarks: DMD is a disease caused by a genetic defect in the dystrophin coding gene. Like affected boys, GRMD dogs are born with the disease and manifest it in their juvenile phase, often dying in early adulthood [9,77–80]. | 6.25 |

| **2. SYMPTOMATOLOGY AND NATURAL HISTORY (SNH) VALIDATION** | |
| --- | --- |
| 2.1 Is the model able to replicate the symptoms and co-morbidities commonly present in this disease? If so, which ones? | Score |
| Yes, partially. | 2 |
| 2.1.1 Symptoms modelled |  |
| Reduced grown-up body weight: at birth, no difference is seen regarding body weight. Starting at 1 month and further at 6 months, it is possible to notice a reduced body weight when compared to controls of up to 40% [77]. |  |
| Reduced lifespan: affected dogs live up to ~3 years, a 75% reduction, similar to humans [77]. |  |
| Muscle wasting: comparable to humans for most muscles with significant fibre necrosis, fibrosis and progressive degeneration [77,78,81–83]. |  |
| ECG abnormality: characteristic ECG changes include increased Q/R ratios, decreased PR intervals, and frequent ventricular arrhythmias [79]. |  |
| Cardiomyopathy: myocardial degeneration, fibrosis and mineralisation alongside with decreased cardiac function and congestive heart failure have been reported in GRMD dogs [77,84,85]. |  |
| 2.1.2 Symptoms partially modelled |  |
| Loss of ambulation: complete loss of ambulation occurs in about 1/3 of GRMD dogs, although dogs which maintain ambulation lose mobility considerably. The reason for this is probably less related to the disease, but more to the fact that dogs are quadrupeds as was suggested that the main reason for the loss of ambulation in humans is more due to the difficulty in balancing the upper body than lower limbs muscle weakness [77,80,86]. |  |
| 2.1.3 Symptoms not modelled | Score |
| Cognitive and CNS defects: not reported (unclear). |  |
| 2.2 Is the natural history of the disease similar to humans regarding: |  |
| 2.2.1 Time to onset; | Score |
| Yes, completely.  Remarks: although GRMD dogs have a somewhat high rate of neonatal death (~25%) not seen in either mdx mice or humans [77,78], the first clinical signs appear at 6-9 weeks, analogous to humans at 2-4 years [9,79,80]. | 2.50 |
| 2.2.2 Disease progression; | Score |
| Yes, partially.  Remarks: in humans, first symptoms start around 2.5 years, with loss of ambulation around 10 to 15 years [11–13]. In GRMD dogs, muscular atrophy along with joint involvement (including jaw mobility), abnormal gait, decreased respiratory function and cardiomyopathy appears at around 6 months of age [9,77,83]. Similar to DMD, there is a honeymoon phase between 6 and 10 months wherein the disease is relatively stable [9,77,87]. Loss of ambulation is infrequent, with only around 1/3 of GRMD dogs losing ambulation completely, although they do lose mobility considerably [77,80,86]. | 1.25 |
| 2.2.3 Duration of symptoms; | Score |
| Yes, completely.  Remarks: symptoms in DMD are lifelong due to permanent muscle degeneration. In GRMD dogs, muscle wasting and degeneration are progressive, leading to permanent damage and significantly reduced lifespan like in humans (9). | 2.5 |
| 2.2.4 Severity. | Score |
| Yes, partially.  Remarks: muscle regeneration in dogs is less prominent than in mice and on par with humans, leading to significant muscle degeneration. The overall loss of muscle strength and function is strikingly similar to humans, however, the infrequency of loss of ambulation makes the manifestation in dogs somewhat less severe than in humans. | 1.25 |

| **3. GENETIC VALIDATION** | |
| --- | --- |
| 3.1 Does this species also have orthologous genes and/or proteins involved in the human disease? | Score |
| Yes.  Remarks: DMD is caused by the lack of dystrophin, thus the dystrophin gene alongside with utrophin (a functional and structural analogue) have been included in this section [31]. The dog has orthologous genes for both of them [88,89]. | 4.17 |
| 3.1.1 Dystrophin |  |
| The human dystrophin gene (Gene ID: 1756) is the largest gene found in humans measuring a total of 2.4 Mb located at Xp21.2-p21.1 [34]. Dystrophin forms parts of the dystrophin-glycoprotein complex (DGC), responsible for connecting the inner cytoskeleton and the extracellular matrix [34]. In dogs, the dystrophin gene (Gene ID: 606758) is located at chromosome X [88]. |  |

| 3.1.2 Utrophin |  |
| --- | --- |
| The human utrophin gene (Gene ID: 7402) is located in humans at 6q24.2 [35]. Utrophin is a structural and functional analogue to dystrophin, being also present at neuromuscular synapses and myotendinous junctions [35]. Utrophin upregulation is thought to be a compensation mechanism partially responsible for the milder phenotype in mdx mice [35]. In dogs, the utrophin gene (Gene ID: 442965) is located at chromosome 1 [89]. |  |
| 3.2 If so, are the relevant genetic mutations or alterations also present in the orthologous genes/proteins? | Score |
| Yes, partially.  Remarks: DMD is caused by deletions, duplications, small mutations or other smaller rearrangements of the gene that codes for dystrophin in chromosome X [31]. In that sense, DMD being an X-linked inherited disorder, the GRMD dog has the same aetiology (spontaneous mutations) [90]. Nevertheless, GRMD dogs have a point mutation in exon 6, which causes the skipping of exon 7 leading to the truncated form of dystrophin [75]. This is not the case for human patients who have other alterations that lead to dystrophin deficiency. | 2.09 |
| 3.3 If so, is the expression of such orthologous genes and/or proteins similar to the human condition? | Score |
| Yes, completely. | 4.16 |
| 3.3.1 Dystrophin |  |
| Yes, completely.  Remarks: Like DMD patients, GRMD dogs are dystrophin-deficient not expressing full-length dystrophin [75]. |  |
| 3.3.2 Utrophin |  |
| Yes, completely.  Remarks: Utrophin upregulation was reported at the sarcolemma of dystrophic pups and adults, like in humans [36,37,91]. |  |

| **4. BIOCHEMICAL VALIDATION** | |
| --- | --- |
| 4.1 If there are known pharmacodynamic (PD) biomarkers related to the pathophysiology of the disease, are they also present in the model? | Score |
| Yes, completely.  Remarks: high levels of creatine kinase (CK) – a marker of muscle damage, with the muscle-type variants being predominant in plasma are also present in GRMD dogs [38,82,92–94]. Pyruvate Kinase (PK) is also used as a marker of muscle damage and it is present at high levels in DMD boys, however, it has secondary importance (and it is considerably less reliable as a biomarker than CK) as a tracker of disease progression [38]. It is included here for information purposes. It is not clear whether GRMD dogs have high levels of PK. | 3.13 |
| 4.2 Do these PD biomarkers behave similarly to humans’? | Score |
| Yes, completely.  Remarks: CK levels are increased in GRMD dogs, like in humans [38,82,92–94]. There is no information on PK levels in the literature searched. | 3.13 |
| 4.3 If there are known prognostic biomarkers related to the pathophysiology of the disease, are they also present in the model? | Score |
| N/A.  Remarks: Currently, DMD has no prognostic biomarker which is validated and widely used in clinical practice. Many biomarkers are still under exploratory phase. A list of biomarkers being explored can be found in the supplementary material from Guiraud et al, 2015 and in the paper from Hathout et al, 2016 [39,40] | - |
| 4.4 Do these prognostic biomarkers behave similarly to humans’? | Score |
| N/A. |  |

| **5. AETIOLOGICAL VALIDATION** | |
| --- | --- |
| 5.1 Is the aetiology of the disease similar to humans’? | Score |
| Yes, partially.  Remarks: DMD is caused by deletions, duplications, small mutations or other smaller rearrangements of the gene that codes for dystrophin in chromosome X [31]. In that sense, DMD being an X-linked inherited disorder, the GRMD dog has the same aetiology (spontaneous mutations) [90]. Nevertheless, GRMD dogs have a point mutation in exon 6, which causes the skipping of exon 7 leading to the truncated form of dystrophin [75]. This is not the case for human patients who have other alterations that lead to dystrophin deficiency. | 6.25 |

| **6. HISTOLOGICAL VALIDATION** | |
| --- | --- |
| 6.1 Do the histopathological structures in relevant tissues resemble the ones found in humans? | Score |
| Yes, partially. | 8.33 |
| 6.1.1 Histopathological features modelled |  |
| Muscle regeneration: existent but in similar intensity as humans, being unable to outpace the increasing muscle necrosis [78,81,92,95,96]. |  |
| 6.1.2 Histopathological features partially modelled |  |
| Limb muscle fibrosis: progressive as seen in the human disease, but tend to plateau in adult life [77,78,81,82,84,92,95,96] |  |
| Adipose tissue: fatty infiltration of muscle is less profound in GRMD dogs than in man, sometimes not being evident [77,79,81]. |  |

| **7. PHARMACOLOGICAL VALIDATION** | | | | | | |
| --- | --- | --- | --- | --- | --- | --- |
| 7.1 Are effective drugs in humans also effective in this model? | | | | | | Score |
|  | | | | | | 0.58 |
| 7.1.1 Glucocorticosteroids | | | | | |  |
| 7.1.1.1 Prednisone [55,97,98] | | | | | |  |
| Methodology: studies were searched on 15/09/2016 on PubMed with the string ‘(GRMD OR "golden retriever muscular dystrophy") AND (Prednisone[mesh] OR prednisone OR Apo-Prednisone OR Cortan OR Cortancyl OR Cutason OR Dacortin OR Decortin OR Decortisyl OR Dehydrocortisone OR Deltasone OR Encorton OR Encortone OR Enkortolon OR Kortancyl OR “Liquid Pred” OR Meticorten OR Orasone OR Panafcort OR Panasol OR Predni Tablinen OR Prednidib OR Predniment OR Prednison Acsis OR “Prednison Galen” OR “Prednison Hexal” OR Pronisone OR Rectodelt OR Sone OR Sterapred OR Ultracorten OR Winpred OR delta-Cortisone)’, yielding 4 results; and on Embase with the string ‘(GRMD OR "golden retriever muscular dystrophy") AND (prednisone OR “1, 2 dehydrocortisone” OR “17, 21 dihydroxypregna 1, 4 diene 3, 11, 20 trione” OR ancortone OR apo-prednisone OR biocortone OR colisone OR cortan OR cortidelt OR cortiprex OR cutason OR dacorten OR "de cortisyl" OR decortancyl OR decortin OR "decortin e merck" OR decortine OR decortisyl OR dehydrocortisone OR dekortin OR delitisone OR "dellacort a" OR “delta 1 dehydrocortisone” OR “delta cortelan” OR “delta cortisone” OR “delta dome” OR “delta e” OR “delta prenovis” OR delta-dome OR deltacorten OR deltacortene OR deltacortisone OR deltacortone OR deltasone OR deltison OR deltisona OR deltra OR "di adreson" OR di-adreson OR diadreson OR drazone OR encorton OR encortone OR enkorton OR fernisone OR hostacortin OR insone OR “liquid pred” OR lodotra OR me-korti OR meprison OR metacortandracin OR meticorten OR meticortine OR nisona OR “nsc 10023” OR nsc10023 OR orasone OR orisane OR panafcort OR paracort OR pehacort OR precort OR precortal OR prednicen-m OR prednicorm OR prednicot OR prednidib OR prednison OR “prednisone alcohol” OR “prednisone intensol” OR “prednisone test” OR prednitone OR “pregna 1, 4 diene 3, 11, 20 trione 17, 21 diol” OR pronison OR pronisone OR pronizone OR pulmison OR rayos OR rectodelt OR servisone OR steerometz OR sterapred OR "sterapred ds" OR ultracorten OR urtilone OR winpred), yielding 6 results; which were screened by title and abstract. Of these, 3 articles were included in this section.  Results: the only study which investigated functional outcomes showed mixed results. High-dose prednisone increased fibre calcification and necrosis, and maximal isomeric tibiotarsal extension strength while paradoxically decreasing maximal isomeric flexion strength. No statistically significant difference between treatment groups and untreated GRMD dogs was found regarding joint contracture, muscle hypertrophy. In the other studies, prednisone was shown to increase α7-integrin, laminin- α2 and γ_cyto_ actin expression. Two (2) articles were classified as category I and one (1) as category II. The latter is partially in line with clinical findings. | | | | | |  |
| Reporting Quality | | | | Risk of Bias | | |
| Parameter (N = 3) | Y (%) |  | Y (%) | Parameter (N = 3) | Y/U (%) | |
| Type of Facility | 0.0 | Environmental Enrichment | 0.0 | Allocation Concealment | 0/100 | |
| Type of Cage or Housing | 0.0 | Any Blinding | 33.3 | Blinded Outcome Assessment | 0/100 | |
| Bedding Material | 0.0 | Any Randomisation | 0.0 | Blinded Operations | 0/100 | |
| N Cage Companions | 0.0 | Sample Size | 100 | Random Cage Allocation | 0/100 | |
| Breeding Programme | 100 | Sample Size Calculation | 33.3 | Random Outcome Assessment | 0/100 | |
| Light/Dark Cycle | 0.0 | Acclimatisation | 0.0 | Sequence Generation | 0/100 | |
| Temperature and Humidity | 0.0 | Sex Disclosed | 33.3 | Baseline Characteristics | 0/100 | |
| Quality of the Water (fish) | - | Male/Female/Both  (N = 1) | 0/0/100 | Incomplete Outcome Data | 0/100 | |
| Type of Food | 0.0 | Background Control | - | Selective Outcome Reporting | 100/0 | |
| Access to Food and Water | 0.0 | Background Model | - | Other | 33.3/0 | |

| 7.1.1.2 Deflazacort |  |
| --- | --- |
| Methodology: studies were searched on 15/09/2016 on PubMed with the string ‘GRMD OR "golden retriever muscular dystrophy" AND deflazacort’, yielding no results; and on Embase with the string ‘(GRMD OR "golden retriever muscular dystrophy") AND (deflazacort OR “9 defluorofluazacort” OR azacort OR calcort OR deflan OR defluorofluazacort OR “dl 458” OR “dl 458 it” OR “dl 458it” OR “dl 5458” OR dl458 OR dl458it OR dl5458 OR emflaza OR flantadin OR “fluazacort, difluoro” OR rosilar), yielding 1 result; which was screened by title and abstract. This article was not included in this section. |  |
| 7.1.2 Read-through compound: Ataluren |  |
| Methodology: studies were searched on 15/09/2016 on PubMed with the string ‘searched on PubMed with the string ‘GRMD OR "golden retriever muscular dystrophy" AND (ptc124 OR ataluren)’, yielding no results; and on Embase with the string ‘(GRMD OR "golden retriever muscular dystrophy") AND ("3 [5 (2 fluorophenyl) 1, 2, 4 oxadiazol 3 yl] benzoic acid" OR "3 [5 (2 fluorophenyl) [1, 2, 4] oxadiazol 3 yl] benzoic acid" OR "ataluren sodium" OR "ptc 124" OR ptc124 OR translarna), yielding 1 result; which was screened by title and abstract. This article was not included in this section. |  |
| 7.1.3 Exon skipping: Eteplirsen |  |
| Methodology: studies were searched on 19/09/2016 on PubMed with the string ‘GRMD OR "golden retriever muscular dystrophy" AND ‘(eteplirsen OR avi-4658 OR "avi 4658" OR AVI-4225 OR “AVI 4225”)’’, yielding no results; and on Embase with the string ‘(GRMD OR "golden retriever muscular dystrophy") AND ("3 [5 (2 fluorophenyl) 1, 2, 4 oxadiazol 3 yl] benzoic acid" OR "3 [5 (2 fluorophenyl) [1, 2, 4] oxadiazol 3 yl] benzoic acid" OR "ataluren sodium" OR "ptc 124" OR ptc124 OR translarna), yielding no results. |  |
| 7.2 Are ineffective drugs in humans also ineffective in this model? | Score |
| Two identified drug classes comprise drugs that were tested in humans and failed due to the lack of efficacy: aminopeptidase and leucine peptidase inhibitors; and myostatin inhibitors. | 0.42 |
| 7.2.1 Aminopeptidase and leucine peptidase inhibitors: Bestatin |  |
| Methodology: studies were searched on 19/09/2016 on PubMed with the string ‘("golden retriever muscular dystrophy" OR GRMD) AND (Ubenimex[mesh] OR ubenimex OR “uben 3-amino-2-hydroxy-4-phenylbutyryl-L-leucine” OR bestatin OR “bestatin, (D-Leu)-(R-(R*,R*))-isomer” OR “bestatin, (D-Leu)-(R-(R*,S*))-isomer” OR “bestatin, (D-Leu)-(S-(R*,S*))-isomer” OR “bestatin, (L-Leu)-(R-(R*,R*))-isomer OR bestatin, (L-Leu)-(R-(R*,S*))-isomer” OR “bestatin, (L-Leu)-(S-(R*,R*))-isomer” OR “bestatin, (L-Leu)-(S-(R*,S*))-isomer, hydrochloride”)’, yielding no results; and on Embase with the string ‘(GRMD OR "golden retriever muscular dystrophy") AND (bestatin OR “(3 amino 2 hydroxy 4 phenylbutanoyl) leucine” OR “(3 amino 2 hydroxy 4 phenylbutyryl) leucine; bestatine” OR “n (3 amino 2 hydroxy 1 oxo 4 phenylbutyl) leucine” OR “nk 421” OR ubenimex), yielding no results. |  |
| 7.2.2 Myostatin inhibitors: Stamulumab |  |
| Methodology: studies were searched on 19/09/2016 on PubMed with the string ‘("golden retriever muscular dystrophy" OR GRMD) AND (stamulumab OR “myo 029” OR myo029)’, yielding no results; and on Embase with the string ‘(GRMD OR "golden retriever muscular dystrophy") AND (stamulumab OR “myo 029” OR myo029), yielding no results. |  |
| 7.3 Have drugs with different mechanisms of action and acting on different pathways been tested in this model? If so, which? | Score |
| Yes, partially.  Remarks: only one category II study in GRMD dogs with a glucocorticosteroid – prednisone – has been published so far, evidencing the lack of characterisation of this model regarding the other 4 drug classes identified. | 0.83 |

| **8. ENDPOINT VALIDATION** | |
| --- | --- |
| 8.1 Are the endpoints used in preclinical studies the same or translatable to the clinical endpoints? | Score |
| Yes.  Remarks: the endpoints used in the only preclinical study measured the strength of flexion and extension, which can be considered a proxy of muscle strength. | 6.25 |
| 8.2 Are the methods used to assess preclinical endpoints comparable to the ones used to assess related clinical endpoints? | Score |
| No.  Remarks: the measure of strength for both extension and flexion was taken in anesthetised dogs, in which flexion or extension was caused by electrical stimulation of muscles. DMD boys must run a certain distance or perform certain tasks (e.g. get up the floor without using hands) while being awake and fully aware of the tasks. | 0 |

References

1. van der Worp HB, Howells DW, Sena ES, Porritt MJ, Rewell S, O’Collins V, et al. Can Animal Models of Disease Reliably Inform Human Studies? PLoS Med [Internet]. 2010 Mar 30;7(3):1–8. Available from: https://doi.org/10.1371/journal.pmed.1000245

2. Pound P, Ebrahim S, Sandercock P, Bracken MB, Roberts I. Where is the evidence that animal research benefits humans? BMJ Br Med J. 2004;328(7438):514–7.

3. Perel P, Roberts I, Sena E, Wheble P, Briscoe C, Sandercock P, et al. Comparison of treatment effects between animal experiments and clinical trials: systematic review. BMJ Br Med J [Internet]. 2007;334(7586):197–197. Available from: http://www.bmj.com/cgi/doi/10.1136/bmj.39048.407928.BE

4. Bebarta V, Luyten D, Heard K. Emergency medicine animal research: Does use of randomization and blinding affect the results? Acad Emerg Med. 2003;10(6):684–7.

5. Peers IS, South MC, Ceuppens PR, Bright JD, Pilling E. Can you trust your animal study data? Nat Rev Drug Discov [Internet]. 2014;13(7):560. Available from: http://dx.doi.org/10.1038/nrd4090-c1

6. McGreevy JW, Hakim CH, McIntosh MA, Duan D. Animal models of Duchenne muscular dystrophy: from basic mechanisms to gene therapy. Dis Model Mech [Internet]. 2015;8(3):195–213. Available from: http://dmm.biologists.org/content/8/3/195.full.pdf

7. Bulfield G, Siller WG, Wight PA, Moore KJ. X chromosome-linked muscular dystrophy (mdx) in the mouse. Proc Natl Acad Sci U S A [Internet]. 1984;81(4):1189–92. Available from: http://www.pubmedcentral.nih.gov/articlerender.fcgi?artid=344791&tool=pmcentrez&rendertype=abstract

8. Sicinski P, Geng Y, Ryder-Cook A, Barnard E, Darlison M, Barnard P. The molecular basis of muscular dystrophy in the mdx mouse: a point mutation. Science (80- ). 1989;244(4912):1578–80.

9. Emery AEH. Population frequencies of inherited neuromuscular diseases-A world survey. Neuromuscul Disord. 1991;1(1):19–29.

10. MedlinePlus. Duchenne muscular dystrophy [Internet]. [cited 2016 Sep 1]. Available from: https://medlineplus.gov/ency/article/000705.htm

11. Ciafaloni E, Fox DJ, Pandya S, Westfield CP, Puzhankara S, Romitti PA, et al. Delayed Diagnosis in Duchenne Muscular Dystrophy: Data from the Muscular Dystrophy Surveillance, Tracking, and Research Network (MD STARnet). J Pediatr [Internet]. 2009;155(3):380–5. Available from: http://dx.doi.org/10.1016/j.jpeds.2009.02.007

12. Bello L, Morgenroth LP, Gordish-Dressman H, Hoffman EP, Mcdonald CM, Cirak S, et al. DMD genotypes and loss of ambulation in the CINRG Duchenne Natural History Study. Neurology. 2016;87(4):401–9.

13. Eagle M, Baudouin S V., Chandler C, Giddings DR, Bullock R, Bushby K. Survival in Duchenne muscular dystrophy: Improvements in life expectancy since 1967 and the impact of home nocturnal ventilation. Neuromuscul Disord. 2002;12(10):926–9.

14. Bostick B, Yue Y, Long C, Duan D. Prevention of dystrophin-deficient cardiomyopathy in twenty-one-month-old carrier mice by mosaic dystrophin expression or complementary dystrophin/utrophin expression. Circ Res. 2008;102(1):121–30.

15. Bostick B, Yue Y, Long C, Marschalk N, Fine DM, Chen J, et al. Cardiac expression of a mini-dystrophin that normalizes skeletal muscle force only partially restores heart function in aged Mdx mice. Mol Ther. 2009;17(2):253–61.

16. Hakim CH, Grange RW, Duan D. The Passive mechanical properties of the extensor digitorum longus muscle are compromised in 2 to 20-month-old mdx mice. J Appl Physiol [Internet]. 2011;1656–63. Available from: http://jap.physiology.org/content/early/2011/03/10/japplphysiol.01425.2010%5Cnhttp://jap.physiology.org/content/early/2011/03/10/japplphysiol.01425.2010.full.pdf

17. Pastoret C, Sebille A. Mdx Mice Show Progressive Weakness and Muscle Deterioration With Age. J Neurol Sci. 1995;129(2):97–105.

18. Chamberlain JS, Metzger J, Reyes M, Townsend D, Faulkner JA. Dystrophin-deficient mdx mice display a reduced life span and are susceptible to spontaneous rhabdomyosarcoma. Faseb J. 2007;21(9):2195–204.

19. Stedman HH, Sweeney HL, Shrager JB, Maguire HC, Panettieri R a, Petrof B, et al. The mdx mouse diaphragm reproduces the degenerative changes of Duchenne muscular dystrophy. Vol. 352, Nature. 1991. p. 536–9.

20. Vaillend C, Billard JM, Laroche S. Impaired long-term spatial and recognition memory and enhanced CA1 hippocampal LTP in the dystrophin-deficient Dmdmdxmouse. Neurobiol Dis. 2004;17(1):10–20.

21. Xu S, Shi D, Pratt SJP, Zhu W, Marshall A, Lovering RM. Abnormalities in brain structure and biochemistry associated with mdx mice measured by in vivo MRI and high resolution localized1H MRS. Neuromuscul Disord. 2015;25(10):764–72.

22. Remmelink E, Aartsma-Rus A, Smit AB, Verhage M, Loos M, van Putten M. Cognitive flexibility deficits in a mouse model for the absence of full-length dystrophin. Genes, Brain Behav. 2016;15(6):558–67.

23. Karagan NJ, Richman LC, Sorensen JP. Analysis of verbal disability in duchenne muscular dystrophy. J Nerv Ment Dis [Internet]. 1980;168(7):419–23. Available from: https://www.scopus.com/inward/record.uri?eid=2-s2.0-0018831758&partnerID=40&md5=f07de7d8ddd92b1f82b6c67291405403

24. Dorman C, Hurley AD, D’Avignon J. Language and learning disorders of older boys with Duchenne muscular dystrophy. DevMedChild Neurol [Internet]. 1988;30(3):316–27. Available from: http://www.ncbi.nlm.nih.gov/pubmed/3402673

25. Leibowitz D, Dubowitz V. Intellect and Behaviour in Duchenne Muscular Dystrophy. Dev Med Child Neurol. 1981;23(6):577–90.

26. Anderson SW, Routh DK, Ionasescu V V. Serial position memory of boys with Duchenne muscular dystrophy. Dev Med Child Neurol [Internet]. 1988;30(3):328–33. Available from: http://www.ncbi.nlm.nih.gov/pubmed/3402674

27. Lynch GS, Hinkle RT, Chamberlain JS, Brooks S V, Faulkner J a. Force and power output of fast and slow skeletal muscles from mdx mice 6 – 28 months old. J Physiol. 2001;535(2):591–600.

28. Lefaucheur JP, Pastoret C, Sebille A. Phenotype of dystrophinopathy in old mdx mice. Anat Rec [Internet]. 1995;242(1):70–6. Available from: http://www.ncbi.nlm.nih.gov/pubmed/7604983

29. Grounds MD, Radley HG, Lynch GS, Nagaraju K, De Luca A. Towards developing standard operating procedures for pre-clinical testing in the mdx mouse model of Duchenne muscular dystrophy. Neurobiol Dis. 2008;31(1):1–19.

30. McGeachie JK, Grounds MD, Partridge TA, Morgan JE. Age-related changes in replication of myogenic cells in mdx mice: quantitative autoradiographic studies. J Neurol Sci [Internet]. 1993;119(2):169–79. Available from: http://ac.els-cdn.com/0022510X9390130Q/1-s2.0-0022510X9390130Q-main.pdf?_tid=f9e0a294-2943-11e4-b2f4-00000aacb361&acdnat=1408633446_d3f843b7f01109ce941c131e64dd2d45

31. Ferlini A, Neri M, Gualandi F. The medical genetics of dystrophinopathies: Molecular genetic diagnosis and its impact on clinical practice. Neuromuscul Disord [Internet]. 2013;23(1):4–14. Available from: http://dx.doi.org/10.1016/j.nmd.2012.09.002

32. PubMed. Dmd dystrophin, muscular dystrophy [Mus musculus (house mouse)] [Internet]. [cited 2016 Oct 21]. Available from: https://www.ncbi.nlm.nih.gov/gene/13405

33. PubMed. Utrn utrophin [Mus musculus (house mouse)] [Internet]. [cited 2016 Oct 21]. Available from: https://www.ncbi.nlm.nih.gov/gene/22288

34. PubMed. DMD dystrophin [Homo sapiens (human)] [Internet]. [cited 2016 Oct 21]. Available from: https://www.ncbi.nlm.nih.gov/gene/1756

35. PubMed. UTRN utrophin [Homo sapiens (human)] [Internet]. [cited 2016 Oct 21]. Available from: https://www.ncbi.nlm.nih.gov/gene/7402

36. Behr TM, Fischer P, Mudra H, Theisen K, Spes C, Uberfuhr P, et al. Upregulation of utrophin in the myocardium of a carrier of Duchenne muscular dystrophy. Eur Hear J [Internet]. 1997;18(4):699–700. Available from: http://www.ncbi.nlm.nih.gov/entrez/query.fcgi?cmd=Retrieve&db=PubMed&dopt=Citation&list_uids=9129907

37. Fanin M, Melacini P, Angelini C, Danieli GA. Could utrophin rescue the myocardium of patients with dystrophin gene mutations? J Mol Cell Cardiol. 1999;31(0022–2828):1501–8.

38. Zatz M, Rapaport D, Vainzof M, Passos-Bueno MR, Bortolini ER, Pavanello R de CM, et al. Serum creatine-kinase (CK) and pyruvate-kinase (PK) activities in Duchenne (DMD) as compared with Becker (BMD) muscular dystrophy. J Neurol Sci. 1991;102(2):190–6.

39. Guiraud S, Aartsma-Rus A, Vieira NM, Davies KE, van Ommen G-JB, Kunkel LM. The Pathogenesis and Therapy of Muscular Dystrophies. Annu Rev Genomics Hum Genet [Internet]. 2015;(May):1–28. Available from: http://www.ncbi.nlm.nih.gov/pubmed/26048046

40. Hathout Y, Seol H, Han MHJ, Zhang A, Brown KJ, Hoffman EP. Clinical utility of serum biomarkers in Duchenne muscular dystrophy. Clin Proteomics [Internet]. 2016;13:9. Available from: http://www.pubmedcentral.nih.gov/articlerender.fcgi?artid=4820909&tool=pmcentrez&rendertype=abstract

41. Wissing ER, Millay DP, Vuagniaux G, Molkentin JD. Debio-025 is more effective than prednisone in reducing muscular pathology in mdx mice. Neuromuscul Disord [Internet]. 2010;20(11):753–60. Available from: http://dx.doi.org/10.1016/j.nmd.2010.06.016

42. Guerron AD, Rawat R, Sali A, Spurney CF, Pistilli E, Cha HJ, et al. Functional and molecular effects of arginine butyrate and prednisone on muscle and heart in the mdx mouse model of duchenne muscular dystrophy. PLoS One. 2010;5(6):1–12.

43. Mizunoya W, Upadhaya R, Burczynski FJ, Wang G, Anderson JE. Nitric oxide donors improve prednisone effects on muscular dystrophy in the mdx mouse diaphragm. Am J Physiol Cell Physiol [Internet]. 2011;300(5):C1065-77. Available from: http://www.ncbi.nlm.nih.gov/pubmed/21270295

44. Baudy AR, Sali A, Jordan S, Kesari A, Johnston HK, Hoffman EP, et al. Non-invasive optical imaging of muscle pathology in mdx mice using cathepsin caged near-infrared imaging. Mol Imaging Biol. 2011;13(3):462–70.

45. McIntosh L, Granberg KE, Brière KM, Anderson JE. Nuclear magnetic resonance spectroscopy study of muscle growth, mdx dystrophy and glucocorticoid treatments: Correlation with repair. NMR Biomed. 1998;11(1):1–10.

46. Shawn RA, Mantsch HH, Anderson JE. Infrared spectroscopy of dystrophic mdx mouse muscle tissue distinguishes among treatment groups. J Appl Physiol. 1996;81(5):2328–35.

47. Hinkle RT, Lefever FR, Dolan ET, Reichart DL, Dietrich JA, Gropp KE, et al. Corticortophin releasing factor 2 receptor agonist treatment significantly slows disease progression in mdx mice. BMC Med. 2007;5(18):1–11.

48. Skrabek RQ, Anderson JE. Metabolic shifts and myocyte hypertrophy in deflazacort treatment of mdx mouse cardiomyopathy. Muscle and Nerve. 2001;24(2):192–202.

49. Anderson JE, Mcintosh LM, Poettcker R. Deflazacort but not prednisone improves both muscle repair and fiber growth in diaphragm and limb muscle in vivo in the mdx dystrophic mouse. Muscle and Nerve. 1996;19(12):1576–85.

50. Hartel J V, Granchelli J a, Hudecki MS, Pollina CM, Gosselin LE. Impact of prednisone on TGF-beta1 and collagen in diaphragm muscle from mdx mice. Muscle Nerve. 2001;24(March):428–32.

51. Markham BE, Kernodle S, Nemzek J, Wilkinson JE, Sigler R. Chronic dosing with membrane sealant poloxamer 188 NF improves respiratory dysfunction in dystrophic mdx and mdx/utrophin^-/-^ mice. PLoS One. 2015;10(8):1–24.

52. Granchelli JA, Pollina C, Hudecki MS. Pre-clinical screening of drugs using the mdx mouse. Neuromuscul Disord. 2000;10(4–5):235–9.

53. Sali A, Guerron AD, Gordish-Dressman H, Spurney CF, Iantorno M, Hoffman EP, et al. Glucocorticoid-treated mice are an inappropriate positive control for long-term preclinical studies in the mdx mouse. PLoS One. 2012;7(4):1–9.

54. Yoon SH, Chen J, Grynpas MD, Mitchell J. Prophylactic pamidronate partially protects from glucocorticoid-induced bone loss in the mdx mouse model of Duchenne muscular dystrophy. Bone [Internet]. 2016;90:168–80. Available from: http://dx.doi.org/10.1016/j.bone.2016.06.015

55. Wuebbles RD, Sarathy A, Kornegay JN, Burkin DJ. Levels of α7 integrin and laminin-α2 are increased following prednisone treatment in the mdx mouse and GRMD dog models of Duchenne muscular dystrophy. Dis Model Mech [Internet]. 2013;6:1175–84. Available from: http://www.pubmedcentral.nih.gov/articlerender.fcgi?artid=3759337&tool=pmcentrez&rendertype=abstract

56. de Carvalho SC, Apolinário LM, Matheus SMM, Santo Neto H, Marques MJ. EPA protects against muscle damage in the mdx mouse model of Duchenne muscular dystrophy by promoting a shift from the M1 to M2 macrophage phenotype. J Neuroimmunol [Internet]. 2013;264(1–2):41–7. Available from: http://dx.doi.org/10.1016/j.jneuroim.2013.09.007

57. Anderson JE, Weber M, Vargas C. Deflazacort increases laminin expression and myogenic repair, and induces early persistent functional gain in mdx mouse muscular dystrophy. In: Cell Transplantation. 2000. p. 551–64.

58. St-Pierre SJG, Chakkalakal J V, Kolodziejczyk SM, Knudson JC, Jasmin BJ, Megeney L a. Glucocorticoid treatment alleviates dystrophic myofiber pathology by activation of the calcineurin/NF-AT pathway. FASEB J. 2004;18(15):1937–9.

59. Archer JD, Vargas CC, Anderson JE. Persistent and improved functional gain in mdx dystrophic mice after treatment with L-arginine and deflazacort. FASEB J [Internet]. 2006;20(6):738–40. Available from: http://www.ncbi.nlm.nih.gov/pubmed/16464957

60. Marques MJ, Oggiam DS, Barbin ICC, Ferretti R, Santo Neto H. Long-term therapy with deflazacort decreases myocardial fibrosis in mdx mice. Muscle and Nerve. 2009;40(3):466–8.

61. Pereira JA, Marques MJ, Santo Neto H. Co-administration of deflazacort and doxycycline: A potential pharmacotherapy for Duchenne muscular dystrophy. Clin Exp Pharmacol Physiol. 2015;42(7):788–94.

62. Ségalat L, Grisoni K, Archer J, Vargas C, Bertrand A, Anderson JE. CAPON expression in skeletal muscle is regulated by position, repair, NOS activity, and dystrophy. Exp Cell Res. 2005;302(2):170–9.

63. Anderson JE, Vargas C. Correlated NOS-Iμ and myf5 expression by satellite cells in mdx mouse muscle regeneration during NOS manipulation and deflazacort treatment. Neuromuscul Disord. 2003;13(5):388–96.

64. Kayali R, Ku JM, Khitrov G, Jung ME, Prikhodko O, Bertoni C. Read-through compound 13 restores dystrophin expression and improves muscle function in the MDX mouse model for duchenne muscular dystrophy. Hum Mol Genet. 2012;21(18):4007–20.

65. Alter J, Lou F, Rabinowitz A, Yin H, Rosenfeld J, Wilton SD, et al. Systemic delivery of morpholino oligonucleotide restores dystrophin expression bodywide and improves dystrophic pathology. Nat Med [Internet]. 2006;12(2):175–7. Available from: http://www.nature.com/doifinder/10.1038/nm1345

66. Malerba A, Sharp PS, Graham IR, Arechavala-Gomeza V, Foster K, Muntoni F, et al. Chronic systemic therapy with low-dose morpholino oligomers ameliorates the pathology and normalizes locomotor behavior in mdx mice. Mol Ther [Internet]. 2011;19(2):345–54. Available from: http://dx.doi.org/10.1038/mt.2010.261

67. Sharp PS, Bye-a-Jee H, Wells DJ. Physiological characterization of muscle strength with variable levels of dystrophin restoration in mdx mice following local antisense therapy. Mol Ther [Internet]. 2011;19(1):165–71. Available from: http://www.pubmedcentral.nih.gov/articlerender.fcgi?artid=3017444&tool=pmcentrez&rendertype=abstract

68. Wu B, Xiao B, Cloer C, Shaban M, Sali A, Lu P, et al. One-year treatment of morpholino antisense oligomer improves skeletal and cardiac muscle functions in dystrophic mdx mice. Mol Ther [Internet]. 2011;19(3):576–83. Available from: http://www.scopus.com/inward/record.url?eid=2-s2.0-79952189079&partnerID=tZOtx3y1

69. Sazani P, Ness KPV, Weller DL, Poage D, Nelson K, Shrewsbury ASB. Chemical and mechanistic toxicology evaluation of exon skipping phosphorodiamidate morpholino oligomers in mdx mice. Int J Toxicol. 2011;30(3):322–33.

70. FDA. Eteplirsen - Pharmacology Review [Internet]. [cited 2016 Sep 19]. Available from: http://www.accessdata.fda.gov/drugsatfda_docs/nda/2016/206488Orig1s000PharmR.pdf

71. Kishi M, Kurihara T, Hidaka T, Kinoshita M. The stabilizing effect of bestatin on the resting membrane potentials of X-linked muscular dystrophy mice. JpnJPsychiatry Neurol. 1990;44(3):595–600.

72. Bogdanovich S, Krag TOB, Barton ER, Morris LD, Whittemore L-A, Ahima RS, et al. Functional improvement of dystrophic muscle by myostatin blockade. Nature. 2002;420(6914):418–21.

73. Meier H, H. M, Meier. Myopathies in the dog. Cornell Vet [Internet]. 1958;48(3):313–30. Available from: http://ovidsp.ovid.com/ovidweb.cgi?T=JS&PAGE=reference&D=emcl1&NEWS=N&AN=0008055495%5Cnhttp://ovidsp.ovid.com/ovidweb.cgi?T=JS&CSC=Y&NEWS=N&PAGE=fulltext&D=emcl1&AN=0008055495%5Cnhttp://digitaal.uba.uva.nl:9003/uva-linker?sid=OVID:embase&id=pmid:&id=doi:&

74. Valentine BA, Cooper BJ, Cummings JF, deLahunta A. Progressive muscular dystrophy in a golden retriever dog: light microscope and ultrastructural features at 4 and 8 months. Acta Neuropathol [Internet]. 1986;71(3–4):301–10. Available from: http://www.ncbi.nlm.nih.gov/entrez/query.fcgi?cmd=Retrieve&db=PubMed&dopt=Citation&list_uids=3799143

75. Sharp NJH, Kornegay JN, Van Camp SD, Herbstreith MH, Secore SL, Kettle S, et al. An error in dystrophin mRNA processing in golden retriever muscular dystrophy, an animal homologue of Duchenne muscular dystrophy. Genomics. 1992;13(1):115–21.

76. Kornegay JN. The golden retriever model of Duchenne muscular dystrophy. Skelet Muscle. 2017;7(9):1–21.

77. Valentine BA, Cooper BJ, de Lahunta A, O’Quinn R, Blue JT. Canine X-linked muscular dystrophy. An animal model of Duchenne muscular dystrophy: Clinical studies. J Neurol Sci. 1988;88(1–3):69–81.

78. Valentine BA, Cooper BJ. Canine X-linked muscular dystrophy: Selective involvement of muscles in neonatal dogs. Neuromuscul Disord. 1991;1(1):31–8.

79. Valentine B a, Winand NJ, Pradhan D, Moise NS, de Lahunta a, Kornegay JN, et al. Canine X-linked muscular dystrophy as an animal model of Duchenne muscular dystrophy: a review. Am J Med Genet [Internet]. 1992;42(3):352–6. Available from: http://www.ncbi.nlm.nih.gov/pubmed/1536178

80. Barthélémy I, Pinto-Mariz F, Yada E, Desquilbet L, Savino W, Silva-Barbosa SD, et al. Predictive markers of clinical outcome in the GRMD dog model of Duchenne muscular dystrophy. Dis Model Mech [Internet]. 2014;7(11):1253–61. Available from: http://www.ncbi.nlm.nih.gov/pubmed/4213729%5Cnhttp://www.pubmedcentral.nih.gov/articlerender.fcgi?artid=PMC1045041%5Cnhttp://www.ncbi.nlm.nih.gov/pubmed/25261568%5Cnhttp://www.pubmedcentral.nih.gov/articlerender.fcgi?artid=PMC4213729

81. Valentine BA, Cooper BJ, Cummings JF, de Lahunta A. Canine X-linked muscular dystrophy: Morphologic lesions. J Neurol Sci. 1990;97(1):1–23.

82. McCully K, Giger U, Argov Z, Valentine B, Cooper B, Chance B, et al. Canine X-linked muscular dystrophy studied with in vivo phosphorus magnetic resonance spectroscopy. Muscle Nerve. 1991;14(11):1091–8.

83. Valentine BA, Cooper BJ, Gallagher EA. Intracellular calcium in canine muscle biopsies. J Comp Pathol. 1989;100(3):223–30.

84. Cooper BJ, Winand NJ, Stedman H, Valentine BA, Hoffman EP, Kunkel LM, et al. The homologue of the Duchenne locus is defective in X-linked muscular dystrophy of dogs. Nature. 1988;334(6178):154–6.

85. Valentine B a, Cummings JF, Cooper BJ. Development of Duchenne-type cardiomyopathy. Morphologic studies in a canine model. Am J Pathol. 1989;135(4):671–8.

86. Barthelemy I, Barrey E, Aguilar P, Uriarte A, Le Chevoir M, Thibaud JL, et al. Longitudinal ambulatory measurements of gait abnormality in dystrophin-deficient dogs. Bmc Musculoskelet Disord. 2011;12:1–11.

87. Fan Z, Wang J, Ahn M, Shiloh-Malawsky Y, Chahin N, Elmore S, et al. Characteristics of magnetic resonance imaging biomarkers in a natural history study of golden retriever muscular dystrophy. Neuromuscul Disord [Internet]. 2014;24(2):178–91. Available from: http://www.pubmedcentral.nih.gov/articlerender.fcgi?artid=4065593&tool=pmcentrez&rendertype=abstract

88. PubMed. DMD dystrophin [Canis lupus familiaris (dog)] [Internet]. [cited 2016 Oct 21]. Available from: https://www.ncbi.nlm.nih.gov/gene/606758

89. PubMed. UTRN utrophin [Canis lupus familiaris (dog)] [Internet]. [cited 2016 Oct 21]. Available from: https://www.ncbi.nlm.nih.gov/gene/442965

90. Cooper BJ, Valentine BA, Wilson S, Patterson DF, Concannon PW. Canine muscular dystrophy: confirmation of X-linked inheritance. J Hered [Internet]. 1988;79(6):405–8. Available from: http://jhered.oxfordjournals.org/content/79/6/405.full.pdf

91. Lanfossi M, Cozzi F, Bugini D, Colombo S, Scarpa P, Morandi L, et al. Development of muscle pathology in canine X-linked muscular dystrophy. I. Delayed postnatal maturation of affected and normal muscle as revealed by myosin isoform analysis and utrophin expression. Acta Neuropathol. 1999;97(2):127–38.

92. Kornegay JN, Tuler SM, Miller DM, Levesque DC. Muscular dystrophy in a litter of golden retriever dogs. Muscle Nerve. 1988;11(10):1056–64.

93. Valentine BA, Blue JT, Cooper BJ. The effect of exercise on canine dystrophic muscle. Ann Neurol [Internet]. 1989 Oct [cited 2016 Sep 16];26(4):588. Available from: http://www.ncbi.nlm.nih.gov/pubmed/2817833

94. Valentine B, Blue J, Shelley S, Cooper B. Increased Serum Alanine Aminotransf erase Activity Associated With Muscle Necrosis in the Dog. J Vet Iternal Med. 1990;4:140–3.

95. Nguyen F, Cherel Y, Guigand L, Goubault-Leroux I, Wyers M. Muscle lesions associated with dystrophin deficiency in neonatal golden retriever puppies. J Comp Pathol. 2002;126(2–3):100–8.

96. Cozzi F, Cerletti M, Luvoni GC, Lombardo R, Brambilla PG, Faverzani S, et al. Development of muscle pathology in canine X-linked muscular dystrophy. II. Quantitative characterization of histopathological progression during postnatal skeletal muscle development. Acta Neuropathol [Internet]. 2001;101(5):469–78. Available from: http://www.ncbi.nlm.nih.gov/entrez/query.fcgi?cmd=Retrieve&db=PubMed&dopt=Citation&list_uids=11484818

97. Liu JMK, Okamura CS, Bogan DJ, Bogan JR, Childers MK, Kornegay JN. Effects of prednisone in canine muscular dystrophy. Muscle and Nerve. 2004;30(6):767–73.

98. Hanft LM, Bogan DJ, Mayer U, Kaufman SJ, Kornegay JN, Ervasti JM. Cytoplasmic γ-actin expression in diverse animal models of muscular dystrophy. Neuromuscul Disord. 2007;17(7):569–74.
